# Early, very high-titre convalescent plasma therapy in clinically vulnerable individuals with mild COVID-19: an international, randomised, open-label trial

**DOI:** 10.1016/j.ebiom.2025.105613

**Published:** 2025-02-27

**Authors:** Simone Hoffmann, Eva Schrezenmeier, Maxime Desmarets, Fabian Halleck, Antoine Durrbach, Lynn Peters, Anna-Teresa Tremmel, Alina Seidel, Marita Führer, Friederike Bachmann, Jens Schrezenmeier, Jochen Greiner, Sixten Körper, Henrike Hofmann, Carolin Ludwig, Christiane Vieweg, Bernd Jahrsdörfer, Klemens Budde, Michael Schmidt, Jan Münch, Nizar Joher, Etienne Daguindau, Beate Grüner, Gaëlle Brunotte, Charline Vauchy, Erhard Seifried, Daniel Bradshaw, Lise J. Estcourt, David J. Roberts, Eric Toussirot, Bart Rijnders, Pierre Tiberghien, Hubert Schrezenmeier

**Affiliations:** a Institute of Clinical Transfusion Medicine and Immunogenetics, German Red Cross Blood Transfusion Service Baden-Württemberg-Hessen and University Hospital Ulm, and Institute of Transfusion Medicine, University of Ulm, Ulm, Germany; b Department of Nephrology and Medical Intensive Care, Charité-Universitätsmedizin Berlin, Berlin, Germany; c Université Marie et Louis Pasteur, EFS, Inserm, RIGHT (UMR 1098), Besançon, France; d CHU Besançon, Inserm, Centre d'Investigation Clinique (CIC 1431), Besançon, France; e Department of Nephrology, AP-HP Hôpital Henri Mondor, Créteil, Île-de-France, France; f INSERM UMR1186, Universite Paris Saclay, France; g Division of Infectious Diseases, Department of Internal Medicine III, University Hospital of Ulm, Ulm, Germany; h Clinics Ostalb, Stauferklinikum, Mutlangen, Germany; i Institute of Molecular Virology, Ulm University Medical Centre, Ulm, Germany; j Division of Haematology, Oncology, and Cancer Immunology, Medical Department, Charité - Universitätsmedizin Berlin, Corporate Member of Freie Universität Berlin, Humboldt-Universität zu Berlin, and Berlin Institute of Health, Berlin, Germany; k Department of Internal Medicine, Diakonie Hospital Stuttgart, Stuttgart, Germany; l Institute of Transfusion Medicine and Immunohematology, German Red Cross Blood Transfusion Service Baden-Württemberg - Hessen, Frankfurt, Germany; m Haematology Department, CHU Besançon, Besançon, France; n Virus Reference Department, UK Health Security Agency, London, UK; o NHS Blood and Transplant, Oxford, Oxfordshire, UK; p Radcliffe Department of Medicine, University of Oxford, Oxford, Oxfordshire, UK; q Department of Internal Medicine, Section of Infectious Diseases and Department of Medical Microbiology and Infectious Diseases, Erasmus MC, University Medical Center, Rotterdam, The Netherlands; r Etablissement Francais du Sang, La Plaine Saint-Denis, Île-de-France, France

**Keywords:** Convalescent plasma, COVID-19, Randomised trial, Neutralising antibody, SARS-CoV-2

## Abstract

**Background:**

COVID-19 convalescent plasma (CCP) is a treatment option for COVID-19. This study investigated the safety and efficacy of early, very high-titre CCP in immunocompromised individuals with mild COVID-19.

**Methods:**

This randomised, controlled, open-label trial assessed CCP in immunocompromised patients (n = 120) with mild COVID-19 in 10 clinical trial centres across Germany, France, and the Netherlands. Patients were randomised 1:1 to receive either standard of care (SoC) alone (SoC group) or SoC and 2 units of CCP. Most patients (89.7%) had received ≥3 SARS-CoV-2 vaccinations. The primary endpoint was hospitalisation for progressive COVID-19 symptoms or death by day 28 after randomisation, analysed on a modified intention-to-treat basis (117 patients). The safety analysis included the full analysis set. The trial is registered with EudraCT 2021-006621-22, and ClinicalTrials.gov, NCT05271929.

**Findings:**

Between April 11, 2022 and November 27, 2023, 120 patients were enrolled. Patients in the CCP group received a median of 559 ml CCP from convalescent, vaccinated donors with very high levels of SARS-CoV-2 antibodies (median 81,810 IU/ml) at a median 4 days after symptom onset. The primary outcome occurred in 5/58 patients (8.6%) in the SoC group and in 0/59 patients (0%) in the CCP group, difference −8.6% (95% confidence interval of difference −19% to −0.80%; p-value 0.027; Fisher's exact test). The course of SARS-CoV-2 antibodies in the patients demonstrated a passive transfer of antibodies by the CCP, in particular neutralising effects against new SARS-CoV-2 variants. Whole genome sequencing of SARS-CoV-2 in patients during follow-up showed significant intra-host viral evolution, but without differences between groups. CCP was well tolerated.

**Interpretation:**

Early administration of high-titre CCP can prevent hospitalisation or death in immunocompromised patients with mild COVID-19.

**Funding:**

Support-e project (10.13039/501100007601European Union's Horizon 2020 Programme), German Federal Ministry of Education and Research, ZonMw, the 10.13039/501100001826Netherlands Organisation for Health Research and Development.

**Research in context**

**Evidence before this study**

We searched PubMed databases from Jan 01, 2020 to Dec 31, 2024, with no language restrictions, for randomised trials or meta-analyses evaluating the effect of convalescent plasma in immunocompromised patients with mild COVID-19 not hospitalised for COVID-19. We used the terms (“COVID-19”, “COVID”, “SARS-CoV-2” or “Coronavirus”) AND (“convalescent plasma”, “CCP”, “hyperimmune plasma”, “passive immunization”, “passive immunotherapy”, “plasma therapy”) AND (“immune deficiency”, “immunodeficiency”, “immune defect”, “immunocompromised”, “immune suppression”, “immunosuppression”, “transplantation”, “T cell defect”, “B cell defect”, “outpatient”). Four randomised trials that included immunocompromised patients and 4 meta-analyses were identified, two that specifically analysed the treatment of outpatients with COVID-19 and one that specifically analysed the treatment of immunocompromised patients. In the latter, three randomised clinical trials enrolling 214 participants and 5 matched cohorts were included. All controlled trials enrolled patients already hospitalised for COVID-19 and due to the eligibility criteria, the immunocompromised patients were only a subgroup of the total study population in the four randomised trials. Mortality was observed more commonly among standard of care recipients compared with convalescent plasma (risk of mortality according to pooled risk ratio 0.63 (95% CI 0.50–0.79)). At the time the studies included in this meta-analysis were conducted, no specific antiviral therapy with monoclonal antibodies and antiviral substances was available. Furthermore, with a few exceptions, only plasma from non-vaccinated convalescent donors was used. A recent observational study reported the clinical outcome of immunocompromised COVID-19 outpatients who received contemporary COVID-19 specific therapy with or without concomitant treatment with very high-titre COVID-19 convalescent plasma from vaccinated donors. A relative risk reduction for hospitalisation of 65% (p = 0.046) was reported.

**Added value of this study**

COVIC-19 is a randomised trial to include only immunocompromised patients and to compare the early administration of very high-titre plasma in addition to standard of care with standard of care alone. The standard therapy included anti-S monoclonal antibodies and antivirals. The vast majority of patients had multiple vaccinations against SARS-CoV-2. The convalescent plasma was obtained from selected vaccinated and convalescent donors and had very high concentrations of anti-SARS-CoV-2 antibodies, also confirmed by neutralisation tests. Thus, the COVIC-19 trial provides information on the use of convalescent plasma in the light of the rapid evolution of the pandemic, a vaccine-immunised population and the advent of other treatment options. We found that compared with standard of care alone, high-titre convalescent plasma given within 7 days of symptom onset reduced hospitalisation or death in immunocompromised patients with mild COVID-19. Our data are consistent with the results of subgroup analyses of randomised trials that included, among other patient groups, immunocompromised patients, as well as with the results of cohort studies that also suggested a benefit of convalescent plasma. The course of antibody titres in the plasma of recipients compared to the standard therapy group shows the passive transfer of SARS-CoV-2 antibodies, particularly against newer variants. Genotyping of SARS-CoV-2 from nasopharyngeal specimens indicated no immune selection of new variants.

**Implications of all the available evidence**

The available evidence indicates that administration of very high-titre convalescent plasma to immunocompromised patients with mild COVID-19 before hospital admission can prevent hospitalisation and/or death. Contemporaneous convalescent plasma remains an immediate treatment option for vulnerable patients that remain at risk of progression to severe disease. In the event of another epidemic with a pathogen for which treatment with passive humoural immunity appears possible, clinical studies should be carried out as early as possible, especially in immunocompromised patients, with early administration of convalescent plasma with the highest possible antibody titres.

## Introduction

COVID-19 convalescent plasma (CCP) has been explored as one of the treatment options for COVID-19. A systematic review and meta-analysis of clinical trials concluded with high certainty of evidence that CCP for individuals with moderate to severe disease does not reduce mortality and has little to no effect on clinical improvement or deterioration.^1^ The majority of CCP trials included hospitalised patients with moderate to severe COVID-19.^1^ Few trials enrolled outpatients.^2^, ^3^, ^4^, ^5^, ^6^, ^7^ Cohort studies^8^, ^9^, ^10^, ^11^, ^12^ or subgroup analyses of randomised trials^13^, ^14^, ^15^, ^16^ suggested benefit of CCP in immunocompromised inpatients. Definitive evidence, in the current epidemiology circumstances, for CCP effectiveness in immunocompromised outpatients is absent.

The randomised, open-label COVIC-19 clinical trial was developed based on the lessons learnt from previous CCP studies, but has several distinctive aspects compared to previous CCP studies.^17^ First, only patients with immune deficiency, who are less likely to mount a strong immune response to vaccination, were included.^18^^,^^19^ Second, the standard of care (SoC) included S-protein monoclonal antibodies or antiviral drugs (Molnupiravir, Nirmatrelvir/Ritonavir, Remdesivir). Third, only CCP units from selected, convalescent, vaccinated donors (“superimmunised donors”) were administered.^20^^,^^21^ Moreover, CCP was administered very early after onset of mild COVID-19. Finally, the cross-neutralisation capacity of the transfused CCP units, the dynamics of SARS-CoV-2 antibodies in the recipients and their reactivity against new SARS-CoV-2 variants were studied and causal virus and those variants evolving during infection were characterised by whole genome sequencing.

In this randomised controlled clinical trial, we investigated whether very high-titre CCP from convalescent and vaccinated donors, administered 7 days of symptom onset, would prevent hospitalisation or death in immunocompromised patients with mild COVID-19.

## Methods

### Trial design

The clinical trial “Early, Very High-Titre Convalescent Plasma Therapy in Clinically Vulnerable Individuals with Mild COVID-19” (Acronym COVIC-19) was an investigator-initiated, randomised, open-label, multicentre study conducted across 10 centres in Germany, France, and the Netherlands. Details of the trial design are provided in the Supplementary Appendix (S) (pp 4–11) and have been published previously.^17^ The trial protocol was approved by the Ethical Committee of University of Ulm (number 41/22) and the local ethical committee at each trial site and by relevant regulatory agencies.

### Patients

**Fig. 1:**
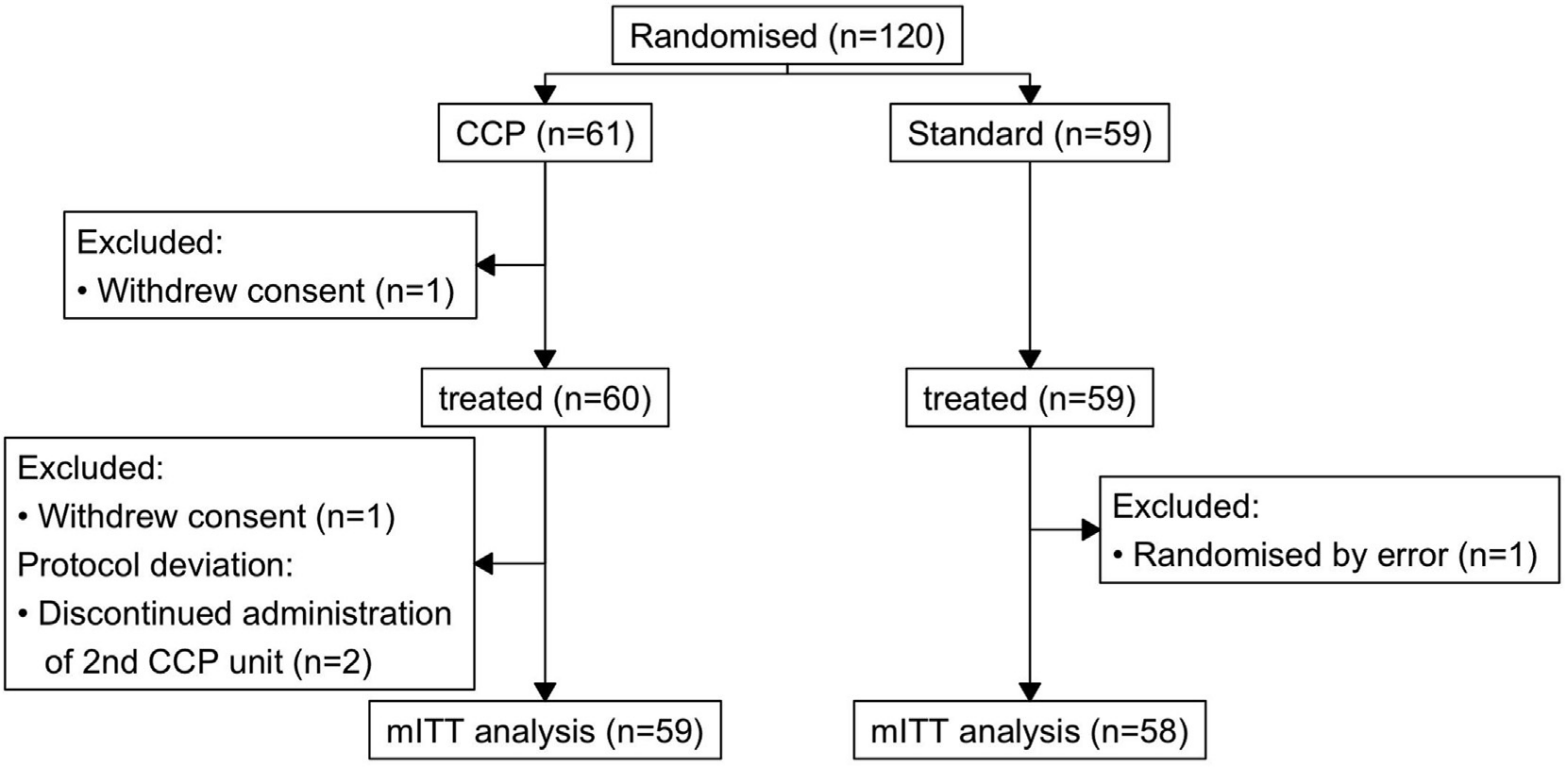
**Trial profile**. One patient was randomised by error. The patient in the SoC group was already hospitalised for COVID-19. Two patients in the CCP group withdrew consent, one patient before and one after administration of CCP.

The trial enrolled patients 18 years of age or older with acquired or congenital immune deficiency and mild COVID-19 within 7 days of symptom onset. All patients provided written informed consent. The inclusion and exclusion criteria are provided in the Appendix (p 4–5). A total of 120 patients were enrolled in Germany (100 patients, 7 centres), the Netherlands (11 patients, 1 centre), France (9 patients, 2 centres) (Fig. 1).

### Randomisation and masking

Eligible patients were randomised using a central web-based randomisation service (CleanWeb, Telemedicine Technologies; Boulogne Billancourt, France). Patients were allocated based on a pre-specified randomisation list. Randomisation was performed at a 1:1 ratio, blocked (with randomly varying block sizes of two and four) and stratified by country.

### Procedures

Patients received SoC plus CCP (CCP group) or SoC alone (SoC group) at a 1:1 ratio. Patients in the CCP group received two ABO-compatible CCP units (200–350 ml each) within 7 days of symptom onset. CCP was obtained by aphaeresis from vaccinated, convalescent donors with very high titres of anti-SARS-CoV-2 antibodies. Plasma contained an anti-SARS-CoV-2 antibody concentration ≥4.000 BAU/ml measured by the QuantiVac anti-SARS-CoV-2 IgG ELISA (Euroimmun, Lübeck, Germany, cat. no. EI 2606-9601-10 G) or ≥20.000 IU/ml measured by the anti-SARS-CoV-2 Elecsys test (Roche, Basel, Switzerland, cat. no. 09 289 275 190) or a minimum neutralising antibody titre of 1:640 against delta (B1.617.2), omicron (B1.1.529), or any future SARS-CoV-2 variant.

The following COVID-19 medications were authorised as standard of care in patients enrolled in the study as pre-exposure prophylaxis, post-exposure prophylaxis, as well as early treatment: anti-SARS-CoV-2 monoclonal antibodies (including Casirivimab/Imdevimab, Regdanvimab, Sotrovimab, and Tixagevimab/Cilgavimab) and antiviral drugs (Molnupiravir, Nirmatrelvir/Ritonavir, and Remdesivir).

### Outcomes

The primary endpoint was the proportion of participants with at least one overnight stay in hospital for progressive COVID-19 symptoms, or who died, by day 28 after randomisation.

Secondary endpoints included proportion of participants with hospitalisation for progressive COVID-19 symptoms, or death by day 14 (stages 4–10 of the WHO scale) and all-cause mortality by day 28, 90, and 180 after randomisation. A full list of endpoints and details regarding randomisation, laboratory methods for measurement of SARS-CoV-2 antibodies and their neutralising capacity and the detection of SARS-CoV-2 in nasopharyngeal specimens and the whole genome sequencing of SARS-CoV-2 are provided in the Appendix (pp 5–10).

### Statistical analysis

We estimated that the risk of severe COVID-19 in the vulnerable population would be 30% and that the intervention would reduce the risk of hospitalisation by 50%.^2^ Based on a Z-Test, a two-tailed α of 0.05, a 1-β of 0.90, and a relative risk of 0.5, 316 patients are required. The sample size was increased to 340 to account for missing data/loss to follow up. More information on sample size calculations have been previously published^17^ and are presented in the Appendix (p 5). All efficacy analyses were performed on the modified intention to treat (mITT) population in which patients were analysed according to treatment allocation and patients enrolled in error were excluded. The primary endpoint was analysed using Fisher's exact test under bilateral hypotheses, with risk difference between groups and 95% confidence interval (95% CI) provided.^22^ The significance threshold was set at p < 0.05. Predefined subgroup analyses of the primary endpoint were performed according to sex, age, and the use of anti-SARS-CoV-2 monoclonal antibodies or antivirals. Risk difference or absolute differences (with 95%-CI) are provided for secondary outcomes and no hypothesis tests were performed. The width of confidence intervals was not adjusted for multiplicity. The safety analysis was performed on 119 patients as treated. In accordance with the criteria of the study protocol, the study was terminated prematurely on January 18, 2024, before the planned number of patients had been reached. Due to the significant decline in the number of newly diagnosed infections and the difficulties in opening new centres in the outgoing pandemic, recruitment declined. The Data Safety Monitoring Board recommended to stop the trial.

The trial is registered with EudraCT 2021-006621-22, and ClinicalTrials.gov, NCT05271929.

### Role of funders

The funders of the study had no role in study design, data collection, data analysis, data interpretation, or writing of the report.

## Results

### Patients

120 patients were enrolled between 11th April 2022 and 27th November 2023. 117 patients were included in the modified intention to treat analysis (mITT), 59 patients in the CCP arm and 58 patients in the SoC arm (Fig. 1). All patients were immunocompromised (Table 1, Appendix Figures S1 and S2). Most patients had a coexisting condition at baseline (Table 1). More than 67% of patients in both arms received immunosuppressive treatment at baseline (Appendix Table S1). The time from symptom onset of SARS-CoV-2 infection to randomisation was 3 days (IQR 2–4, Appendix Figure S3). The most common symptoms were cough, fatigue, headache, rhinorrhoea, sore throat, and fever (Appendix Table S2). All infections with a virus of identifiable lineage were caused by Omicron B.1.1.529 (Table 1, Appendix Figure S4). Almost all of the study population had received at least three SARS-CoV-2 vaccinations (Table 1, Appendix Table S3, Appendix Figure S1). Patients had low antibody levels at enrolment (Appendix Figure S5). Concentration of anti-SARS-CoV-2 antibodies (IgG) by QuantiVac ELISA was 149.1 BAU/ml (IQR 36.9–592.9) (Appendix Figure S5). While the GenScript Neutralisation assay demonstrated neutralising activity against wild-type virus (73%, IQR 37.5%–94.2%), the neutralisation of Omicron was low (3.5%, IQR 0%–11.3%) (Appendix Figure S5).

**Table 1:** Characteristics of the patients at inclusion.

Characteristic	Overall n = 117^a^	SoC group n = 58^a^	CCP group n = 59^a^
**Age**			
Median (Q1, Q3)	57 (44, 65)	57 (47, 63)	57 (43, 66)
Min, Max	25, 82	25, 78	28, 82
**Sex at birth**			
Male	68 (58.1%)	33 (56.9%)	35 (59.3%)
Female	49 (41.9%)	25 (43.1%)	24 (40.7%)
**Blood group**			
A	44 (37.6%)	22 (37.9%)	22 (37.3%)
B	14 (12.0%)	8 (13.8%)	6 (10.2%)
AB	9 (7.7%)	5 (8.6%)	4 (6.8%)
O	50 (42.7%)	23 (39.7%)	27 (45.8%)
**Immunodeficiency**^b^	117 (100.0%)	58 (100.0%)	59 (100.0%)
Lymphoid malignancy	14 (12.0%)	5 (8.6%)	9 (15.3%)
Myeloid malignancy	8 (6.8%)	3 (5.2%)	5 (8.5%)
Solid tumour	1 (0.9%)	1 (1.7%)	0 (0.0%)
Allogenic HSCT	3 (2.6%)	2 (3.4%)	1 (1.7%)
Organ transplantation	88 (75.2%)	47 (81.0%)	41 (69.5%)
B cell deficiency	2 (1.7%)	0 (0.0%)	2 (3.4%)
T cell deficiency	1 (0.9%)	0 (0.0%)	1 (1.7%)
**Country of enrolment**			
Germany	97 (82.9%)	49 (84.5%)	48 (81.4%)
France	9 (7.7%)	3 (5.2%)	6 (10.2%)
Netherland	11 (9.4%)	6 (10.3%)	5 (8.5%)
**Anti-SARS-CoV-2 monclonal antibodies**^c^			
Tixagevimab/Cilgavimab	52 (44.4%)	26 (44.8%)	26 (44.1%)
Sotrovimab	31 (26.5%)	18 (31.0%)	13 (22.0%)
**Antivirals**^c^			
Nirmatrelvir/Ritonavir	15 (12.8%)	3 (5.2%)	12 (20.3%)
Remdesivir	10 (8.5%)	8 (13.8%)	2 (3.4%)
Molnupiravir	1 (0.85%)	0 (0%)	1 (1.7%)
**Medical condition (any)**	117 (100.0%)	58 (100.0%)	59 (100.0%)
**Type of condition**			
Obesity (BMI > 30)	15 (12.8%)	7 (12.1%)	8 (13.6%)
Chronic cardiac disease	29 (24.8%)	17 (29.3%)	12 (20.3%)
Hypertension	100 (85.5%)	49 (84.5%)	51 (86.4%)
Chronic pulmonary disease (not asthma)	6 (5.1%)	3 (5.2%)	3 (5.1%)
Asthma	4 (3.4%)	1 (1.7%)	3 (5.1%)
Chronic kidney disease (stage 1–4)	92 (78.6%)	47 (81.0%)	45 (76.3%)
Chronic liver disease	12 (10.3%)	5 (8.6%)	7 (11.9%)
Chronic neurological disease	8 (6.8%)	2 (3.4%)	6 (10.2%)
Rheumatoid disease, lupus or psoriasis	5 (4.3%)	5 (8.6%)	0 (0.0%)
Cerebrovascular disease	6 (5.1%)	1 (1.7%)	5 (8.5%)
Diabetes	14 (12.0%)	4 (6.9%)	10 (16.9%)
Current smoker	6 (5.1%)	3 (5.2%)	3 (5.1%)
Asplenia or spleen disease	1 (0.9%)	1 (1.7%)	0 (0.0%)
Malignant neoplasm	25 (21.4%)	9 (15.5%)	16 (27.1%)
Other	105 (89.7%)	52 (89.7%)	53 (89.8%)
**Past COVID-19 infection**^d^ (>90 days prior to enrolment)	11 (9.4%)	7 (12.1%)	4 (6.8%)
**SARS-CoV-2 vaccination** (at least one dose)	111 (96.5%)	56 (98.2%)	55 (94.8%)
Unknown	2	1	1
**Number of SARS-CoV-2 vaccinations**			
0	4 (3.5%)	1 (1.8%)	3 (5.2%)
1	2 (1.8%)	1 (1.8%)	1 (1.7%)
2	3 (2.6%)	2 (3.6%)	1 (1.7%)
3	30 (26.3%)	15 (26.8%)	15 (25.9%)
4	75 (65.8%)	37 (66.1%)	38 (65.5%)
Unknown	3	2	1
**Time from last vaccine dose to randomisation** (days)			
Median (Q1; Q3)	234 (168; 335)	240 (144; 366)	218 (176; 329)
Min, max	44, 754	44, 552	107, 754
Unknown	45	23	22
**Variant causing the current SARS-CoV-2 infection**			
Alpha B.1.1.7	0 (0.0%)	0 (0.0%)	0 (0.0%)
Beta B.1.351	0 (0.0%)	0 (0.0%)	0 (0.0%)
Gamma P.1	0 (0.0%)	0 (0.0%)	0 (0.0%)
Delta B.1.617.2	0 (0.0%)	0 (0.0%)	0 (0.0%)
Omicron B.1.1.529	108 (92.3%)	57 (98.3%)	51 (86.4%)
Iota B.1.526	0 (0.0%)	0 (0.0%)	0 (0.0%)
Kappa B.1.617.1	0 (0.0%)	0 (0.0%)	0 (0.0%)
Lambda C.37	0 (0.0%)	0 (0.0%)	0 (0.0%)
Mu B.1.621	0 (0.0%)	0 (0.0%)	0 (0.0%)
Other/unassigned	1 (0.9%)	0 (0.0%)	1 (1.7%)
Unknown/result not clear	5 (4.3%)	1 (1.7%)	4 (6.8%)
Unknown/no sample	3 (2.6%)	0 (0.0%)	3 (5.1%)
**VOIs**^e^	0.0 (0%)	0.0 (0%)	0.0 (0%)
**Omicron variants**			
BA.1 lineage	0 (0.0%)	0 (0.0%)	0 (0.0%)
BA.2 lineage	29 (26.9)	14 (24.6%)	15 (29.4%)
BA.4 lineage	2 (1.9%)	1 (1.8%)	1 (1.7%)
BA.5 lineage	40 (37.0%)	22 (37.5%)	18 (35.3)
BF.7/BF.14	5 (4.6%)	3 (5.4%)	2 (3.9%)
BQ1/BQ1.1	4 (3.7%)	3 (5.4%)	1 (1.7%)
XBB/XBB.1	18 (16.7%)	11 (19.6%)	7 (13.7%)
EG.5.1.	3 (2.8%)	1 (1.8%)	2 (3.9%)
other	2 (1.9%)	0 (0.0%)	2 (3.9%)
Unknown – result not clear	5 (4.6%)	2 (3.6%)	3 (5.9%)

a n (%).

b Some patients fulfilled more than one criterion. The intersection between the criteria for immune deficiency is shown in UpSet plots in Appendix Figure S2.

c Medication given up to day 28 (evaluation of primary endpoint) are listed. Some patients received more than one SARS-CoV-2 medication. The intersection is shown in UpSet Plots in Appendix Figure S6.

d Six of eleven patients received monoclonal antibodies for treatment of this prior COVID-19 (3 sotrovimab, 3 casirivimab/imdevimab), none received antivirals.

e Variant of interest.

The CCP group and SoC groups were similar in terms of demographic characteristics, underlying immune deficiency, comorbidity, SARS-CoV-2 variant causing the current infection, symptoms, SARS-CoV-2 vaccination status and immune response to vaccination (Table 1). Median follow-up was 182 days (IQR 180–187) and 180 days (IQR 179–184) in the CCP and the SoC groups, respectively.

### Study treatment

Patients in both arms could receive mAbs and antiviral drugs according to national recommendations and local availability, as authorised in the study protocol (Appendix Table S4, Appendix Figure S6). After a median interval of 4 days from symptom onset, patients in the CCP group received a median total of 559 ml CCP (IQR 534–577 ml; Appendix Figure S7), with an anti-SARS-CoV-2-IgG concentration of 11,104 BAU/ml (IQR 8,453–12,279 BAU/ml) and 81,810 IU/ml (IQR 52,664–120,230 IU/ml) measured by QuantiVac and Elecsys, respectively (Appendix Figure S8) and good neutralising capacity (Appendix Figure S9). The majority of donors were most likely infected by Delta, BA.1 and BA.2. CCP units were transfused at a median of 116 days (IQR 66–205 days) after collection (Appendix Figure S10).

### Clinical outcomes

**Fig. 2:**
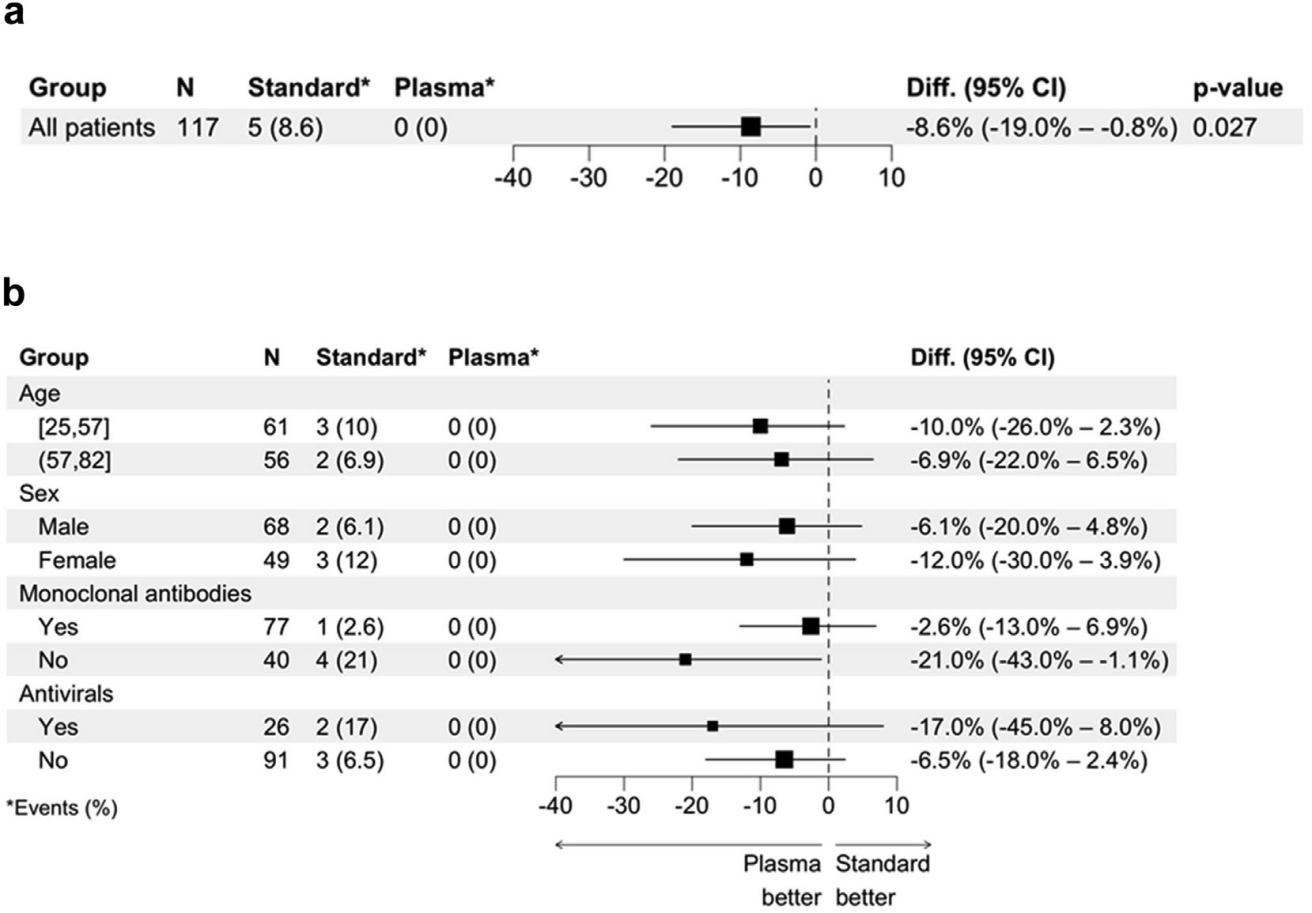
**Forest plot of absolute risk differences in primary outcome of the mITT group (a) and subgroups (b)**.

Five patients (8.6%) in the SoC group versus 0 (0%) in the CCP group were hospitalised for progressive COVID-19 symptoms (n = 4) (three for dyspnoea and lung complications and one for fever) or died (n = 1; after 4 days) within 28 days of randomisation (p = 0.027, Fisher's exact test) (Fig. 2a). Events were rated as COVID-19-related by an adjudication committee, blinded to treatment allocation. Fig. 2 shows the primary outcome at day 28 as well as subgroup analyses with no difference in the subgroups regarding age, sex and anti-SARS-CoV-2 antivirals. The effect of CCP was most pronounced in the group that did not receive monoclonal S-antibody (Fig. 2b).

At day 14, four patients (6.9%) in the SoC group versus 0 (0%) in the CCP group were hospitalised for progressive COVID-19 symptoms (n = 3) or died (n = 1, difference −6.9%, 95% CI −16% to 0.51%, Appendix Table S5). At day 28, supplemental oxygen was required by two patients (3.4%) in the SoC group versus no patients (0%) in the CCP group. At day 180, two patients in the SoC group (3.4%) and one patient (1.7%) in the CCP group had died (Appendix Table S5). All other secondary outcomes are detailed in the Appendix (Tables S5 and S6, Figure S11).

### Adverse events

Thirty-five patients (59%) in the CCP group experienced 78 adverse events and 42 patients (71%) in the SoC group experienced 87 adverse events (Table 2). Twelve patients (20%) in the CCP group experienced 14 serious adverse events and 21 patients (36%) in the SoC group experienced 33 serious adverse events, none were considered to be related to the study intervention (Table 2, Appendix Table S7).

**Table 2:** Summary of adverse events through day 180.

	Standard care (n = 59)^a^	CCP (n = 59)^a^
**Total number of adverse events**	87	78
**Relatedness**		
Related to intervention	0 (0%)	2 (2.6%)
Unknown	1	2
**Outcome**		
Solved or back to previous status	64 (74%)	58 (74%)
Improvement	9 (10%)	11 (14%)
Worsening	0 (0%)	0 (0%)
Not recovered or stable	4 (4.6%)	1 (1.3%)
Fatal^b^	4 (4.6%)	1 (1.3%)
Unknown	6 (6.9%)	7 (9.0%)
**Number of serious adverse event**	33	14
**Relatedness**		
Related to intervention	0/33 (0%)	0/14 (0%)
Unknown	0	0
**Outcome**		
Resolved or back to previous status	22 (67%)	12 (86%)
Improvement	4 (12%)	1 (7.1%)
Worsening	0 (0%)	0 (0%)
Not recovered or stable	2 (6.1%)	0 (0%)
Fatal^b^	4 (12%)	1 (7.1%)
Unknown	2 (6.1%)	0 (0%)

a All treated patients with follow up were included in the safety analysis.

b One death occurred in a patient who was enrolled in error (already hospitalised at time of enrolment due to COVID-19) who was excluded from the mITT but included in the safety analysis. This patient experienced two serious adverse events.

### SARS-CoV-2 antibodies in patients

**Fig. 3:**
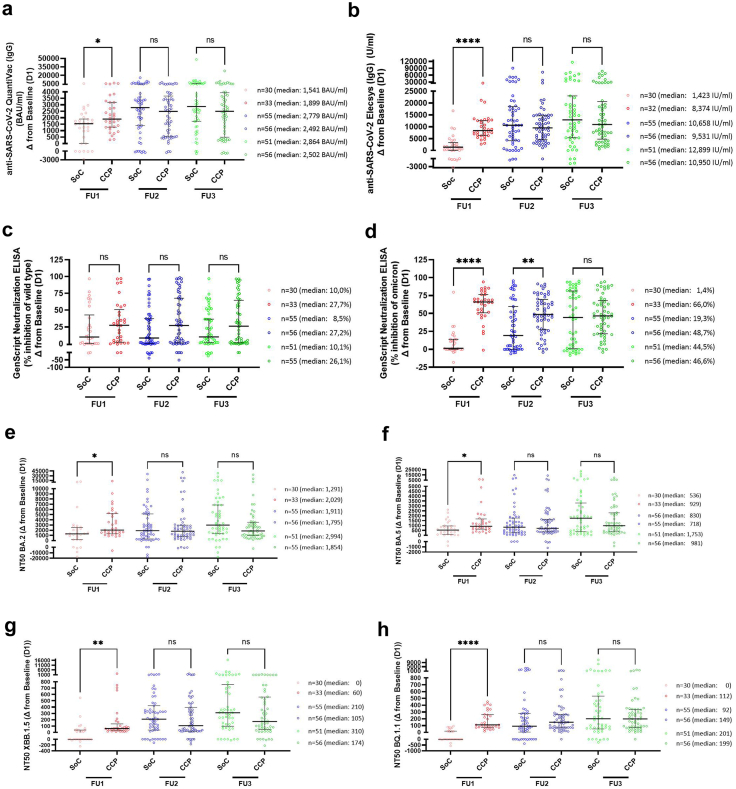
**Change in serum SARS-CoV-2 antibody concentration and neutralisation capacity of patients through day 28**. Change of the anti-SARS-CoV-2 antibody concentration (panels **a–d**) and change of the neutralising capacity (panels **e–h**) in serum of patients on follow up visit 1 (FU1, day 3; red symbols), visit 2 (FU2, day 14; blue symbols), and visit 3 (FU3, day 28; green symbols) compared to baseline measured by anti-SARS-CoV-2-QuantiVac-ELISA (IgG) (**a**), Elecsys Anti-SARS-CoV-2 S (**b**) or Genscript Surrogate Neutralisation Test against wild-type (**c**) or Omicron (**d**) and neutralising titre (50% inhibition of pseudovirus; NT50) measured against BA.2 (**e**), BA.5 (**f**), XBB.1.5 (**g**) or BQ.1.1 (**h**). Results are shown for SoC group (light symbols) and the CCP group (bold symbols). For analysis of the course of the anti-SARS-CoV-2 antibodies in the patients, the difference between the antibody concentrations at the follow-up examinations (follow-up 1, day 3; follow-up 2, day 14; and follow-up 3, day 28) and the concentration before the start of therapy (baseline) was calculated for each individual patient and each follow-up time point (Δ from baseline values). Positive values indicate an increase of the antibody concentration or NT50, negative values a decrease of antibody concentration or NT50 compared to baseline. Horizontal lines indicate the median and error bars the interquartile range. The median baseline levels of the SoC and CCP groups in the QuantiVac ELISA were 163.6 BAU/ml and 138.3 BAU/ml (n.s.) (see Appendix Figure S5a), in the Elecsys-Assay 771.0 and 540.0 IU/ml (n.s.) (see Appendix Figure S5b), in the GenScript Assay against wild type 75.9% and 69.3% (n.s.) (see Appendix Figure S5c) and in the GenScript Assay against omicron 2.2% and 4.0% (n.s.) (see Appendix Figure S5d). The mean baseline NT50 titres in the SoC and CCP groups against BA.2 were 76.3 and 93.8 (n.s.) (see Appendix Figure S5e), against BA.5 were 43.5 and 34.8 (n.s.) (see Appendix Figure S5f), against XBB.1.5 were 10 and 10 (n.s.) (see Appendix Figure S5g), and against BQ.1.1 were 10 and 10 (n.s.) (see Appendix Figure S5h). Geometric means of the change of SARS-CoV-2 antibody concentrations and neutralisation capacity are summarised in Appendix Table S8. For each follow visit, the Δ from baseline between the SoC group and the CCP group was compared by Kruskal–Wallis test followed by Dunn's test for correction of multiple comparisons. The p-values for the pairwise comparisons are p > 0.05 (ns; not significant), ∗p < 0.05, ∗∗p < 0.01, ∗∗∗∗p < 0.0001.

On day 3 (Follow-up (FU) visit 1) there was a significantly greater increase in serum concentration of patients' SARS-CoV-2 antibodies relative to the baseline value on day 1 (i.e., before CCP administration in the CCP group), in the CCP group compared to the SoC group using both the QuantiVac ELISA (Fig. 3a) and the Elecsys CLIA (Fig. 3b). At further follow-up visits, there was no significant difference between the groups (Fig. 3a and b). Neutralisation of the wild-type virus did not differ between the CCP group and the SoC group at any follow-up visit in the GenScript surrogate neutralisation test (Fig. 3c), but there was a significant increase in the neutralisation of Omicron in the CCP group compared to baseline both on days 3 and 14, but not on day 28 (Fig. 3d).

In the pseudovirus neutralisation assays the NT50 values in the CCP group versus the SoC group increased significantly more on day 3 relative to baseline in the inhibition of BA.2, BA.5 (p < 0.05; Fig. 3e and f), XBB.1.5 (p < 0.01; Fig. 3g), and BQ.1.1 (p < 0.001; Fig. 3h). In the pseudovirus neutralisation assays there was no significant difference between the groups at further follow-up visits (Fig. 3e–h). In the pseudovirus neutralisation assays, the NT50 values continued to increase in both groups over time (Fig. 3e–h). The geometric means of the change of serum SARS-CoV-2 antibody concentration and neutralisation capacity on day 3, day 14, and day 28 for the SoC and CCP groups in the assays shown in Fig. 3a–h are summarised in Appendix Table S8.

### Viral load in nasopharyngeal swabs and viral evolution

The viral load in the nasopharyngeal swabs as measured by PCR decreased over time without significant difference between the CCP and the SoC groups (Appendix Figure S12).

**Fig. 4:**
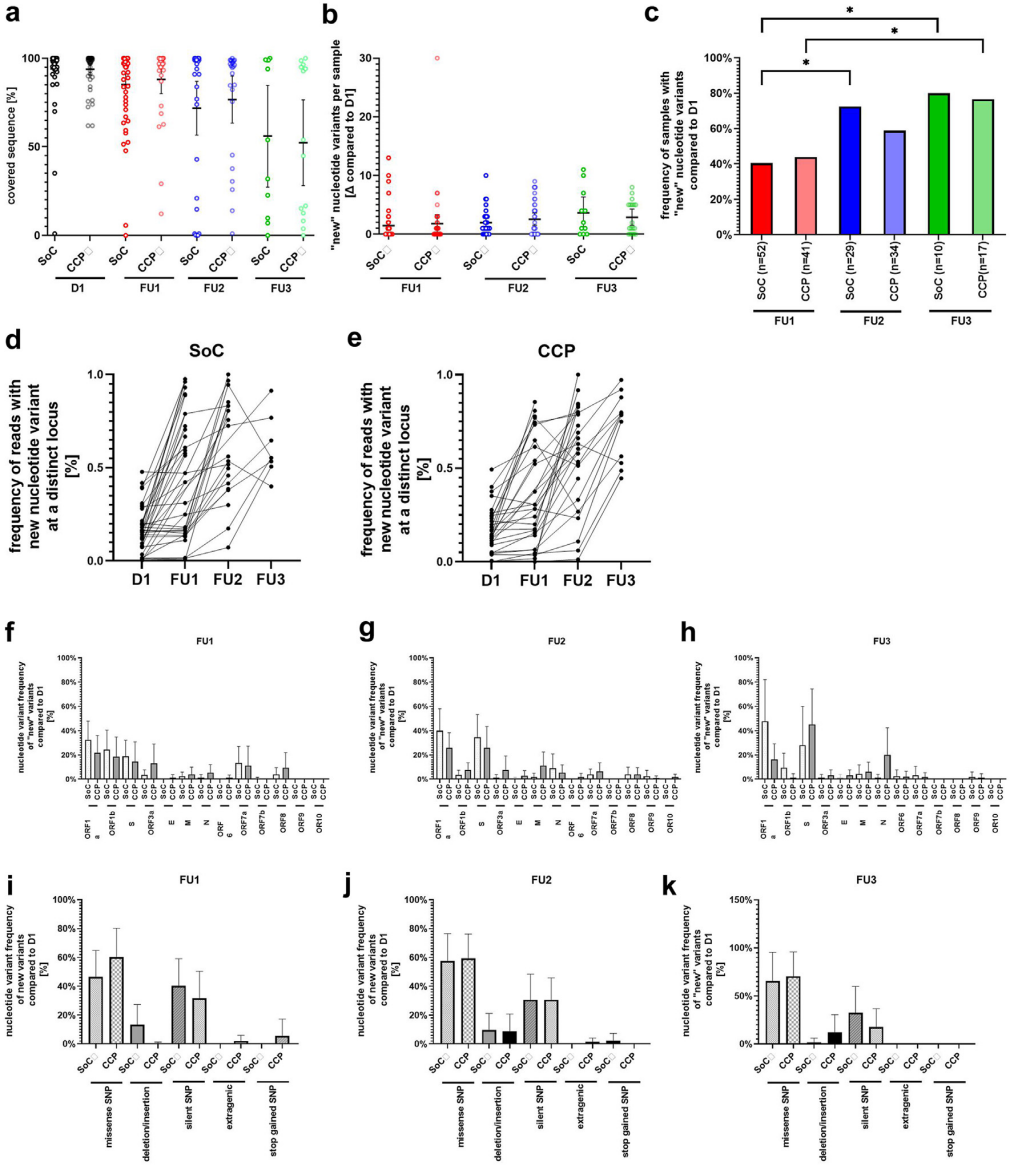

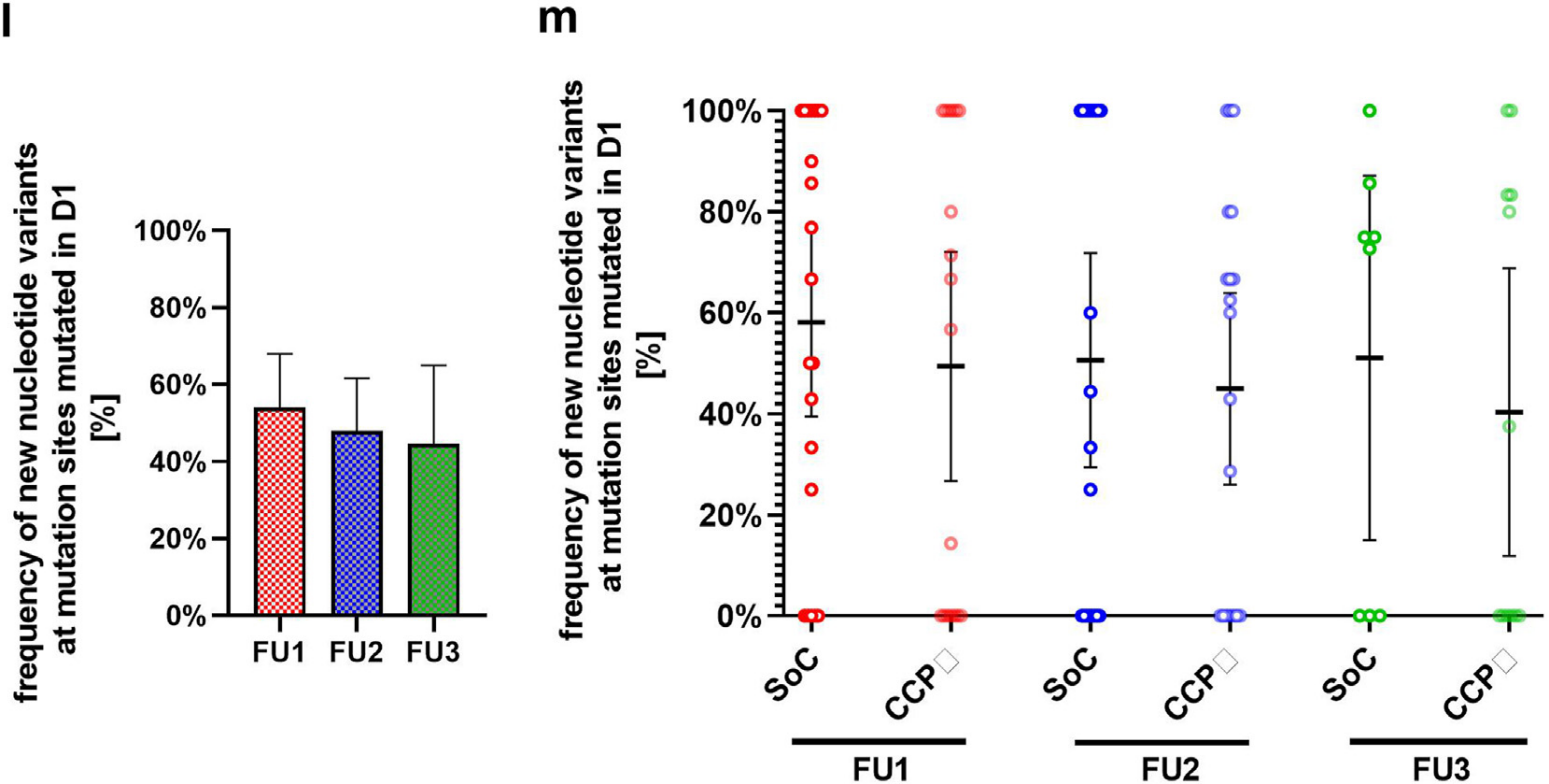
**Sequencing of SARS-CoV-2 virus from nasopharyngeal specimens of patients**. Sequencing was performed with Oxford Nanopore. Sequences obtained on follow up visit 1 (FU1, day 3), visit 2 (FU2, day 14), and visit 3 (FU3, day 28) were compared to baseline (D1). Sequence coverage of SARS-CoV-2 genome from nasopharyngeal swabs in the SoC group and the CCP group were compared. Horizontal lines indicate the mean and error bars the 95% confidence interval of the mean. Mean values were compared between the SoC and the CCP group by Kruskal–Wallis test followed by Dunn's test for correction of multiple comparisons. The p-values for all pairwise comparisons were p > 0.05 (not significant) (**a**). Panel (**b**) indicate the count of new acquired mutations compared to baseline (D1). Horizontal lines indicate the mean and error bars the 95% confidence interval of the mean. Mean values were compared between the SoC and the CCP group by Kruskal–Wallis test followed by Dunn's test for correction of multiple comparisons. The p-values for all pairwise comparisons were p > 0.05 (not significant). (**c**) The proportion of patients with still detectable SARS-CoV-2 and new mutations in the SoC and CCP group. P-values for comparison of treatment groups were calculated by Fisher's exact test and were not significant (p > 0.05), whereas the difference within treatment group during time was significant (∗p < 0.05). Nucleotide variant evolution of new variants compared to baseline data (D1) for SoC (**d**) or CCP (**e**) group is visualised by the frequency of reads with the distinct variant call connected by vertical lines from D1 to FU. (**f–h**) Proportion of new variants in SARS-CoV-2 genes were calculated for every sample with at least one new variant. (**i–k**) Frequencies of different mutation types of new variants were calculated for every sample with at least one new variant. (**f–k**) Means (horizontal line; error bars = 95% confidence interval) were compared between treatment groups SoC and CCP and showed no significant difference calculated by Kruskal–Wallis test followed by Dunn's test for correction of multiple comparisons. (**l**) For the analysis we only considered samples with at least one new mutation compared to baseline (D1). Among those, we calculated the proportion of mutations localised at sites that already exhibited mutations at baseline (D1) in comparison to the Wuhan reference sequence. Depicted are means with error bars as 95% confidence interval. (**m**) Variants depicted in L were compared between SoC and CCP treatment and showed no significant difference calculated by Kruskal–Wallis test followed by Dunn's test for correction of multiple comparisons.

Whole genome nanopore sequencing was performed in all cases when virus was detected by PCR. The percentage of sequences covered per sample decreased over time (Fig. 4a) and follow-up samples revealed changes in viral genome sequence which were not present in baseline samples (Fig. 4b and c), but with no difference between groups, although variant proportion evolved dynamically with time (Fig. 4d and e). New sequence changes were detected in many genes (Fig. 4f–h), particularly in large genes as ORF1a, ORF1b and S. New sequence variants were more common in S than ORF1a and ORF1b, despite a smaller gene size. Most mutations were missense or silent single nucleotide polymorphisms (Fig. 4i–k), with no significant difference between SoC and CCP groups. There was an accumulation of new nucleotide variants at mutation sites which were mutated at baseline (D1) compared to the SARS-CoV-2 reference sequence (Fig. 4l) but these were equally distributed between SoC and CCP groups (Fig. 4m).

## Discussion

The COVIC-19 trial shows very high-titre CCP administered to immunocompromised patients with mild COVID-19 before hospital admission can prevent hospitalisation and/or death. The primary outcome of hospitalisation and/or death occurred in 5/58 (8.6%) in immunocompromised patients in the control (SoC) group and in 0/59 patients (0%) in the CCP group (p-value 0.027). This trial suggests a role for high-titre CCP from immunised donors in managing immunocompromised patients with COVID-19, given the current epidemiology of the disease.

Other randomised trials included hospitalised patients with COVID-19 of whom a subgroup had immune deficiency or cancer,^13^, ^14^, ^15^, ^16^ but did not show efficacy considering the whole trial population. However, in the RECOVERY trial those with cancer experienced a shortened median time to improvement and superior survival with CCP versus the control arm.^15^ In the CORIPLASM trial patients with immunodeficiency had a lower odds of death at 14 and 28 days after CCP although not quite reaching statistical significance.^16^ Also, in the REMAP-CAP trial, CCP demonstrated potential benefit in participants with immunodeficiency.^14^ In a meta-analysis which included randomised trials, matched cohort studies and case series, transfusion of CCP was associated with reduced mortality in immunocompromised COVID-19 patients.^23^

Several aspects that distinguish the COVIC-19 trial from all other previous randomised CCP trials must be considered when interpreting the results:

(i) During the enrolment period the Omicron variants BA.2, then BA.5, later on XBB/XBB.1 became prevalent.^24^ Although, the Omicron lineage predominated, immunocompromised patients continued to exhibit a higher risk of progression to severe COVID-19 compared to the general population, albeit at substantially lower rates compared to previous periods.^25^^,^^26^
(ii) In contrast to previous studies of early CCP treatment^2^^,^^3^^,^^5^, ^6^, ^7^ or trials including immunocompromised patients,^13^, ^14^, ^15^, ^16^ in this trial, most participants (87%) also received other anti-SARS-CoV-2 therapy including anti-SARS-CoV-2 monoclonal antibodies or antivirals, or both (standard therapy according to prevailing national recommendations). The effect of CCP appeared to be more pronounced in participants who did not receive monoclonal anti-S antibodies. Although novel monoclonal antibodies are available in some regions, such as Pemivibart and Sipavibart, recent studies indicate that these antibodies lose antiviral efficacy against variants such as KP1.1, LB.1, and KP3.3.^27^ However, if they retain even limited activity against the variants prevalent in our study population the monoclonal antibodies might in part mask the effect of CCP. CCP may still provide a rapid passive transfer of immunity even in the presence of monoclonal antibodies. In patients not treated with monoclonal antibodies, CCP transfusions provide a rapid transfer of humoural immunity that can prevent worsening of COVID-19.
(iii) Most participants had received at least 3 vaccinations.

Therefore, this COVID-19 trial studied the efficacy of CCP in vaccinated patients when other effective, direct anti-viral treatments were generally available. Due to the waning pandemic, the number of participants recruited was lower than estimated in the sample size calculation. Furthermore, the number of participants reaching the primary endpoint was lower than assumed in the sample size calculation due to the combination of low pathogenicity of the new Omicron variants, the high proportion of vaccinated participants, and the use of antivirals and monoclonal antibodies as SoC.

Consistent with the immune deficiency in the trial population the antibody concentration at baseline was low compared to non-immunocompromised individuals.^19^^,^^28^ Furthermore, the increase in antibody titres in the SoC group as early as day 3 shows the immune response in this vaccinated population irrespective of passively transferred antibodies in the CCP group. Despite impairment in B and T cell memory in immunocompromised patients,^19^^,^^29^ a serological response can be boosted in this population by repeated recall vaccinations and/or temporary withholding of immunousuppression.^29^, ^30^, ^31^

The vaccine-induced immunity was directed against the wild-type virus, as few patients had received a vaccination with the new bivalent vaccine (only available after Sept 2022). In contrast, CCP units afforded high neutralisation capacity against Omicron. Indeed, the significant increase of Omicron neutralisation capacity in the CCP group on day 3 shows a passive transfer of immunity against Omicron with the transfused CCP. The difference in neutralisation capacity between the groups decreased over time, due not only to the natural immune response, but also the waning of passively transferred antibodies. This course of antibody titres over time is similar to a previous study which reported that titres rose significantly faster in the CCP group than that of placebo, but there was no difference between groups after day 14.^6^ The data from the COVIC-19 trial shows that a measurable passive transfer of SARS-CoV-2 antibodies was possible by CCP even given the vaccine-induced immunisation. We did not observe a difference in the time to first negative PCR between the SoC and CCP groups, despite the improved clinical outcomes of the latter, highlighting that any role of serial PCRs for predicting clinical response in this setting may be limited. The same discordant observation between nasal viral load and clinical effect has been observed with remdesivir, which reduced the hospital admission rate by 80% but had no effect on nasal viral load.^32^

A substantial number of changes in the SARS-CoV-2 genome sequences in a broad range of genes was detected in patients with SARS-CoV-2 still present during follow-up visits as previously described.^24^^,^^33^, ^34^, ^35^ These changes have been observed previously, particularly in immunocompromised individuals with prolonged infection and treatment with CCP or monoclonal S-protein antibodies, suggesting that escape mutations are selected in vivo and passive immunisation of immunocompromised patients might promote selection of new variants.^33^, ^34^, ^35^, ^36^ This possibility has not been studied so far in the context of a controlled trial. While our data confirm the substantial viral dynamics in an immunocompromised patient population, some of whom also received monoclonal S-protein antibodies and antivirals, our trial data provide reassurance that CCP did not select for mutations associated with reduced susceptibility to monoclonal antibodies or antiviral therapies.

Several previous CCP trials demonstrate a dose effect with significantly better results only in participants who had received CCP with higher titres.^2^^,^^37^, ^38^, ^39^, ^40^ Thus, the COVIC-19 trial aimed to provide CCP with very high titres and broad neutralising capacity. As confirmed by biochemical assays and neutralisation assays this has been achieved. Also, the median transfused volume of CCP (559 ml) transfused in the COVIC-19 study was about twice as high as in the other studies on early CCP therapy, which administered 250–300 ml of CCP.^2^, ^3^, ^4^, ^5^, ^6^, ^7^

Although neutralising anti-SARS-CoV-2 antibodies have been proposed as a potential key mechanism of action of CCP by inhibiting SARS-CoV-2 binding to its receptors on host cells, other antibody-mediated mechanisms (antibody-dependent cytotoxicity, antibody-dependent phagocytosis, complement-dependant cytotoxicity) and effects by non-antibody components in CCP leading to modulation of T cells, B cells or dendritic cells, cytokine release und the complement and coagulation systems might also contribute to its overall effect.^41^, ^42^, ^43^

Potential limitations of the study include the sample size, a fragility index of 1 and the possible bias due to the open label design.

CCP was well tolerated. There was no evidence that the frequency, severity, type and outcome of AEs or SAEs in CCP group differed from the SoC group, indeed there were fewer AEs and SAEs in the CCP group. Like previous studies, this study does not indicate any new safety issues when very high-titre CCP is administered to patients with COVID-19.^1^^,^^44^

To conclude, this trial provides evidence that very high-titre, contemporaneous CCP remains an immediate treatment option for vulnerable immunocompromised patients, that remain at risk of progression to severe disease. Furthermore, in the event of another pandemic, where clinically effective passive humoural immunity is plausible, this study and others, makes it clear that randomised trials to define the efficacy of early administration of convalescent plasma for patients who are unable to mount their own rapid immune response would be valuable at an early stage. Finally, this trial demonstrates that effective therapy, using plasma from “superimmunised” donors with the highest possible antibody, is feasible as the pandemic evolves.

## Contributors

Conceptualisaton: LJE, DJR, DB, CV, PT, MD, and HS generated the study design and wrote the study protocol.

Project administration: SH, SK, CV, GB, MD, PT, ET, BR, and HS coordinated the trial.

Supervision: HS, AD, and ET, and BR: Principal Investigators in Germany, France, and Netherlands.

Formal analysis: MD trial statistician, developed the statistical analysis plan and performed the statistical analysis. MD, PT, SH, MF, ES, and HS analysed and interpreted data.

Funding acquisition: HS, ES, BR, and PT applied for funding.

Investigation: SK, SH, HH, HS: plasma procurement.

ES, FH, FB, KB, JS, BR, AD, NJ, ED, LP, BG, ATT, SK, HH, JG, and HS conducted clinical trial, patient care, data collection.

AS, JM, CL, CV, MS, and BJ: analysis of SARS-CoV-2 antibodies.

MF: SARS-CoV-2 PCR and sequencing.

Validation: MD, CV, SH, and HS.

Visualisation: SH, MD, MF, and HS.

Writing – original draft: HS, SH, ES, and MD wrote the manuscript.

Writing – review & editing: all authors.

The first three authors and the last author wrote the first draft of the manuscript. All the authors contributed to subsequent drafts. The authors vouch for the completeness and accuracy of the data and for the fidelity of the trial to the protocol. All authors contributed to interpretation of data, manuscript writing and approved the manuscript.

## Data sharing statement

Anonymised data will be available upon request for independent review panel-approved research proposals with a signed data sharing agreement. The data will be available after approval and data sharing agreement is in place until 3 years after publication. The data can be obtained by request to maxime.desmarets@univ-fcomte.fr.

## Declaration of interests

PT is an employee of Établissement Français du Sang, the blood establishment responsible for blood collection, qualification and supply in France. HS, SH, SK, HH, MS are, and ESe was an employee of the German Red Cross Blood Transfusion Service Baden-Württemberg-Hessen (or its affiliates), the establishment responsible for blood collection, qualification, and supply of blood products (including CCP) in several federal states, Germany. LJE and DJR are employees of NHS Blood and Transplant, the blood establishment responsible for blood establishment responsible for blood collection, qualification and supply in England. KB reports grants/contracts with Alexion, Astellas, AstraZeneca, Chiesi, CSL Behring, MSC, Otsuka, Stada, Takeda (all payments to his institution); consulting fees from Aicuris, Alexion, Astellas, AstraZeneca, Bayer, Bristol-Myers Squibb, Carealytics, CareDx, Chiesi, CSL Behring, Fresenius, Hans, HiBio, MSD, Natera, Neovii, Paladin, Pfizer, Pirche, Sanofi, Stada, Takeda, Veloxis, Vifor, Xenothera; honoraria for lectures, presentation, speaker's bureaus manuscript writing or educational events from Astellas, AstraZeneca, Chiesi, Fresenius, Hansa, MSD, Neovii, Paladin, Sanofi, Takeda; support for attending meetings and/or travel from AstraZeneca, Chiesi, Hansa, HiBio, MSC, Neovii, Paladin, Stada, Tadeda, Veloxis; participation on a Data Safety Monitoring Board or Advisory Board for Aicuris, Alexion, Astellas, AstraZenca, Bristol-Myers-Squipp, Carealytics, CareDx, Chiesi, CSL Behring, HiBio, MSC, Natera, Neovii, Paladin, Pfizer, Stada, Takeda, Veloxis, Vifor; leadership or fiduciary role in the German Transplant Organisation and Eurotransplant. ED reports honoraria for honoraria for lectures, presentation, speaker's bureaus manuscript writing or educational events from Janssen, Jazz Pharmaceuticals (France); support for attending meetings from Novartis, Sanofi. JS reports support for attending meetings/travel from Pierre Fabre and stocks from BioNTech, Roche and Johnson & Johnson. The authors declare no other competing interests.

## Appendix ASupplementary data

Supplementary Appendix

Protocol

## References

[1] Iannizzi C., Chai K.L., Piechotta V. (2023). Convalescent plasma for people with COVID-19: a living systematic review. Cochrane Database Syst Rev.

[2] Libster R., Perez M.G., Wappner D. (2021). Early high-titer plasma therapy to prevent severe Covid-19 in older adults. N Engl J Med.

[3] Sullivan D.J., Gebo K.A., Shoham S. (2022). Early outpatient treatment for Covid-19 with convalescent plasma. N Engl J Med.

[4] Alemany A., Millat-Martinez P., Corbacho-Monne M. (2022). High-titre methylene blue-treated convalescent plasma as an early treatment for outpatients with COVID-19: a randomised, placebo-controlled trial. Lancet Respir Med.

[5] Millat-Martinez P., Gharbharan A., Alemany A. (2022). Prospective individual patient data meta-analysis of two randomized trials on convalescent plasma for COVID-19 outpatients. Nat Commun.

[6] Gharbharan A., Jordans C., Zwaginga L. (2023). Outpatient convalescent plasma therapy for high-risk patients with early COVID-19: a randomized placebo-controlled trial. Clin Microbiol Infect.

[7] Korley F.K., Durkalski-Mauldin V., Yeatts S.D. (2021). Early convalescent plasma for high-risk outpatients with Covid-19. N Engl J Med.

[8] Thompson M.A., Henderson J.P., Shah P.K. (2021). Association of convalescent plasma therapy with survival in patients with hematologic cancers and COVID-19. JAMA Oncol.

[9] Hueso T., Godron A.S., Lanoy E. (2022). Convalescent plasma improves overall survival in patients with B-cell lymphoid malignancy and COVID-19: a longitudinal cohort and propensity score analysis. Leukemia.

[10] Biernat M.M., Kolasinska A., Kwiatkowski J. (2021). Early administration of convalescent plasma improves survival in patients with hematological malignancies and COVID-19. Viruses.

[11] Ripoll J.G., Tulledge-Scheitel S.M., Stephenson A.A. (2024). Outpatient treatment with concomitant vaccine-boosted convalescent plasma for patients with immunosuppression and COVID-19. mBio.

[12] Ripoll J.G., Gorman E.K., Juskewitch J.E. (2022). Vaccine-boosted convalescent plasma therapy for patients with immunosuppression and COVID-19. Blood Adv.

[13] Bar K.J., Shaw P.A., Choi G.H. (2021). A randomized controlled study of convalescent plasma for individuals hospitalized with COVID-19 pneumonia. J Clin Invest.

[14] Estcourt L.J., Turgeon A.F., McQuilten Z.K. (2021). Effect of convalescent plasma on organ support-free days in critically ill patients with COVID-19: a randomized clinical trial. JAMA.

[15] Denkinger C.M., Janssen M., Schakel U. (2023). Anti-SARS-CoV-2 antibody-containing plasma improves outcome in patients with hematologic or solid cancer and severe COVID-19: a randomized clinical trial. Nat Cancer.

[16] Lacombe K., Hueso T., Porcher R. (2023). Use of covid-19 convalescent plasma to treat patients admitted to hospital for covid-19 with or without underlying immunodeficiency: open label, randomised clinical trial. BMJ Med.

[17] Desmarets M., Hoffmann S., Vauchy C. (2023). Early, very high-titre convalescent plasma therapy in clinically vulnerable individuals with mild COVID-19 (COVIC-19): protocol for a randomised, open-label trial. BMJ Open.

[18] DeWolf S., Laracy J.C., Perales M.A., Kamboj M., van den Brink M.R.M., Vardhana S. (2022). SARS-CoV-2 in immunocompromised individuals. Immunity.

[19] Rincon-Arevalo H., Choi M., Stefanski A.L. (2021). Impaired humoral immunity to SARS-CoV-2 BNT162b2 vaccine in kidney transplant recipients and dialysis patients. Sci Immunol.

[20] Wang Z., Muecksch F., Schaefer-Babajew D. (2021). Naturally enhanced neutralizing breadth against SARS-CoV-2 one year after infection. Nature.

[21] Seidel A., Hoffmann S., Jahrsdorfer B. (2023). SARS-CoV-2 vaccination of convalescents boosts neutralization capacity against Omicron subvariants BA.1, BA.2 and BA.5 and can be predicted by anti-S antibody concentrations in serological assays. Front Immunol.

[22] Newcombe R.G. (1998). Interval estimation for the difference between independent proportions: comparison of eleven methods. Stat Med.

[23] Senefeld J.W., Franchini M., Mengoli C. (2023). COVID-19 convalescent plasma for the treatment of immunocompromised patients: a systematic review and meta-analysis. JAMA Netw Open.

[24] Markov P.V., Ghafari M., Beer M. (2023). The evolution of SARS-CoV-2. Nat Rev Microbiol.

[25] Pinana J.L., Vazquez L., Heras I. (2024). Omicron SARS-CoV-2 infection management and outcomes in patients with hematologic disease and recipients of cell therapy. Front Oncol.

[26] Yang H., Sun F., He Z. (2024). Clinical feature of omicron infection in children with inborn errors of immunity in China. Front Immunol.

[27] Planas D., Staropoli I., Planchais C. (2024). Escape of SARS-CoV-2 variants KP.1.1, LB.1, and KP3.3 from approved monoclonal antibodies. Pathog Immun.

[28] Jahrsdorfer B., Proffen M., Scholz J. (2022). BNT162b2 booster vaccination elicits cross-reactive immunity against SARS-CoV-2 variants B.1.1.529 and B.1.617.2 in convalescents of all ages. Front Immunol.

[29] Sattler A., Thumfart J., Toth L. (2022). SARS-CoV2 mRNA vaccine-specific B-, T- and humoral responses in adolescents after kidney transplantation. Transpl Int.

[30] Schrezenmeier E., Rincon-Arevalo H., Jens A. (2022). Temporary antimetabolite treatment hold boosts SARS-CoV-2 vaccination-specific humoral and cellular immunity in kidney transplant recipients. JCI Insight.

[31] Osmanodja B., Ronicke S., Budde K. (2022). Serological response to three, four and five doses of SARS-CoV-2 vaccine in kidney transplant recipients. J Clin Med.

[32] Goyal A., Duke E.R., Cardozo-Ojeda E.F., Schiffer J.T. (2022). Modeling explains prolonged SARS-CoV-2 nasal shedding relative to lung shedding in remdesivir-treated rhesus macaques. iScience.

[33] Kemp S.A., Collier D.A., Datir R.P. (2021). SARS-CoV-2 evolution during treatment of chronic infection. Nature.

[34] McCarthy K.R., Rennick L.J., Nambulli S. (2021). Recurrent deletions in the SARS-CoV-2 spike glycoprotein drive antibody escape. Science.

[35] Cele S., Gazy I., Jackson L. (2021). Escape of SARS-CoV-2 501Y.V2 from neutralization by convalescent plasma. Nature.

[36] Abbasi J. (2021). Researchers tie severe immunosuppression to chronic COVID-19 and virus variants. JAMA.

[37] Joyner M.J., Carter R.E., Senefeld J.W. (2021). Convalescent plasma antibody levels and the risk of death from Covid-19. N Engl J Med.

[38] Körper S., Weiss M., Zickler D. (2021). Results of the CAPSID randomized trial for high-dose convalescent plasma in patients with severe COVID-19. J Clin Invest.

[39] Korper S., Gruner B., Zickler D. (2022). One-year follow-up of the CAPSID randomized trial for high-dose convalescent plasma in severe COVID-19 patients. J Clin Invest.

[40] Salazar E., Perez K.K., Ashraf M. (2020). Treatment of coronavirus disease 2019 (COVID-19) patients with convalescent plasma. Am J Pathol.

[41] Hoffmann M., Kleine-Weber H., Schroeder S. (2020). SARS-CoV-2 cell entry depends on ACE2 and TMPRSS2 and is blocked by a clinically proven protease inhibitor. Cell.

[42] Zohar T., Alter G. (2020). Dissecting antibody-mediated protection against SARS-CoV-2. Nat Rev Immunol.

[43] Focosi D., Franchini M., Pirofski L.A. (2021). COVID-19 convalescent plasma is more than neutralizing antibodies: a narrative review of potential beneficial and detrimental co-factors. Viruses.

[44] Franchini M., Cruciani M., Casadevall A. (2024). Safety of COVID-19 convalescent plasma: a definitive systematic review and meta-analysis of randomized controlled trials. Transfusion.

