# Supplementary material for: Early, very high-titre convalescent plasma therapy in clinically vulnerable individuals with mild COVID-19: an international, randomised, open-label trial
Source: eBioMedicine. 2025 Feb 27;113:105613. doi: 10.1016/j.ebiom.2025.105613 (PMC11919330; doi:10.1016/j.ebiom.2025.105613)
Supplement: Supplementary Appendix [file mmc1.pdf]

SUPPLEMENTARY APPENDIX

Early, very high-titre convalescent plasma therapy in clinically vulnerable individuals with mild COVID-19: an international, randomised, multicentre, open-label trial

Simone Hoffmann[1]*, Eva Schrezenmeier[2]*, Maxime Desmarets[3,4], Fabian Halleck[2], Antoine Durrbach[5,6], Lynn Peters[7], Anna-Teresa Tremmel[8], Alina Seidel[9], Marita Führer[1], Friederike Bachmann[2], Jens Schrezenmeier[10], Jochen Greiner[11], Sixten Körper[1], Henrike Hofmann[1], Carolin Ludwig[1], Christiane Vieweg[1], Bernd Jahrsdörfer[1], Klemens Budde[2], Michael Schmidt[12], Jan Münch[9], Nizar Joher[6], Etienne Daguindau[3,13], Beate Grüner[7], Gaëlle Brunotte[4], Charline Vauchy[3,4], Erhard Seifried[12], Daniel Bradshaw[14], Lise J. Estcourt[15,16], David J. Roberts[15,16], Eric Toussirot[3,4], Bart Rijnders[17], Pierre Tiberghien[3, 18]**, Hubert Schrezenmeier[1]** #

**ES, SH equally contributing first authors*
*** PT, HS equally contributing senior authors;* #corresponding author

[1] Institute of Clinical Transfusion Medicine and Immunogenetics, German Red Cross Blood Transfusion Service Baden-Württemberg-Hessen and University Hospital Ulm, and Institute of Transfusion Medicine, University of Ulm, Ulm, Germany.

[2] Department of Nephrology andMedical Intensive Care, Charité-Universitätsmedizin Berlin, Berlin, Germany

[3] Université de Franche-Comté, EFS, Inserm, RIGHT (UMR 1098), Besançon, France

[4] CHU Besançon, Inserm, Centre d'Investigation Clinique (CIC 1431), Besançon, France.

[5] Department of Nephrology, AP-HP Hôpital Henri Mondor, Créteil, Île-de-France, France.

[6] INSERM UMR1186, Universite Paris Saclay, France

[7] University Hospital of Ulm, Department of Internal Medicine III, Division of Infectious Diseases, Ulm, Germany.

[8] Clinics Ostalb, Stauferklinikum, Mutlangen, Germany

[9] Institute of Molecular Virology, Ulm University Medical Center, Ulm, Germany

[10] Division of Hematology, Oncology, and Cancer Immunology, Medical Department, Charité - Universitätsmedizin Berlin, Corporate Member of Freie Universität Berlin, Humboldt-Universität zu Berlin, and Berlin Institute of Health, Berlin, Germany

[11] Department of Internal Medicine, Diakonie Hospital Stuttgart, Stuttgart, Germany

[12] Institute of Transfusion Medicine and Immunohematology, German Red Cross Blood Transfusion Service Baden-Württemberg - Hessen, Frankfurt, Germany

[13] Hematology department, CHU Besançon, Besançon, France

[14] Virus Reference Department, UK Health Security Agency, London, UK.

[15] NHS Blood and Transplant, Oxford, Oxfordshire, UK.

[16] Radcliffe Department of Medicine, University of Oxford, Oxford, Oxfordshire, UK.

[17] Department of Internal Medicine, Section of Infectious Diseases and Department of Medical Microbiology and Infectious Diseases, Erasmus MC, University Medical Center, Rotterdam, The Netherlands [18] Etablissement Francais du Sang, La Plaine Saint-Denis, Île-de-France, France

Table of Content

SUPPLEMENTARY METHODS 4
Design of the trial 4
Trial Design and Oversight 4
Details of Ethics and Regulatory Approval 4
Patients 4
Randomisation 5
Sample size calculation 5
Early trial termination 6
Outcome measures 6
Adjudication of the primary endpoint 6
Complete list of secondary endpoints 6
Exploratory Analysis 7
Safety Analysis 7
CCP transfusions and standard treatment 7
Quantification of anti-SARS-CoV-2 antibodies and neutralisation assays 8
SARS-CoV-2 PCR 9
Sequencing of the SARS-CoV-2 9
Further Details on statistical analysis 11
SUPPLEMENTARY RESULTS 12
Patients 12
SARS-CoV-2 infection 12
SARS-CoV-2 immunity at enrolment 13
Study Treatment 13
Secondary Outcomes 14
Adverse Events 14
Passive transfer of antibodies by CCP 14
Viral load in nasopharyngeal swabs 16
Viral Evolution 16
SUPPLEMENTARY FIGURES: 17
Figure S1. Patient characteristics at baseline 17
Figure S2. Upset Plot showing the intersection of criteria of immune deficiency at baseline 18
Figure S3. Time from onset of symptoms to randomisation and to CCP transfusion 19

Figure S4. Enrolment of patients over the trial period ... 20
Figure S5. Anti-SARS-CoV-2 antibody concentration at baseline by randomization group ... 21
Figure S6. Upset Plot of COVID-19 medications (monoclonal antibodies and antivirals) ... 24
Figure S7. Characteristics of transfused CCP units ... 25
Figure S8. Anti-SARS-CoV-2 antibody concentration in transfused CCP units ... 26
Figure S9. Neutralising titers against SARS-CoV-2 in transfused CCP ... 27
Figure S10. SARS-CoV-2 variants which caused infection in donors and time between donation and transfusion of CCP. ... 28
Figure S11. Health-related quality of life dimensions ... 29
Figure S12. Detection of SARS-CoV-2 in nasopharyngeal swabs from patients ... 30
SUPPLEMENTARY TABLES ... 32
Table S1. Concomitant corticosteroids and immunosuppressive drugs at enrolment ... 32
Table S2. Most common COVID-19 symptoms at enrolment*. ... 33
Table S3. SARS-CoV-2 vaccination of trial patients – type of vaccine ... 34
Table S4. CCP treatment, anti-S monoclonal antibodies and antivirals ... 36
Table S5. Secondary outcomes ... 37
Table S6. Secondary Outcomes: Post-COVID-Functional Scale, COVID-19 YRS and EQ5D ... 38
Table S7. Number and type of Adverse Events and Serious Adverse Events ... 39
Table S8. Change in serum anti-SARS-CoV-2 antibody concentration and neutralisation capacity of patients through day 28 ... 43
References ... 44

SUPPLEMENTARY METHODS

The rationale of the trial and a detailed description of the trial protocol has been published previously [1]. The trial planned to enrol patients in two cohorts: unvaccinated patients with a COVID-age ≥ 70 years (cohort 1) and immunocompromised patients with an acquired or congenital immune deficiency (cohort 2). No patient was enrolled in cohort 1. Here we report solely on the results of cohort 2.

Design of the trial

This is a multicentre, open-label randomised clinical trial to evaluate the efficacy and safety of early treatment (≤ 7 days from symptom onset) with very-high titre CCP (from superimmunised vaccinated and convalescent donors) added to SoC (CCP group) vs. SoC alone in vulnerable outpatients with mild COVID-19.

Trial Design and Oversight

The trial was designed and overseen by an international steering committee and supported by unrestricted grants from the European Commission (Support-e project), the German Federal Ministry of Research and Education and the Netherlands Organization for Health Research and Development; the funders had no influence on the design or conduct of the trial and were not involved in data collection or analysis, in the writing of the manuscript, or in the decision to submit it for publication.

Details of Ethics and Regulatory Approval

The study was approved by the Paul-Ehrlich-Institute (Federal Institute for Vaccines and Biomedicines, Langen, Germany) on Feb 08th, 2022 and the French National Agency for the Safety of Medicines and Health Products (ANSM) on Oct 18th, 2022. Approval has been obtained from research ethics board of all involved institutions (University of Ulm ethics committee decision #41/22 on February 14th, 2022; *Comité de protection des personnes Sud-Est I* #2022-A01307-36 on July 11th, 2022; ErasmusMC ethics committee decision #MEC-2022-0365 on August 2nd, 2022). All data collection and management were performed in accordance with the European Union's General Data Protection Regulation (GDPR). Signed informed consent was obtained from all included patients. The trial is registered: EudraCT 2021-006621-22 and Clinical Trials.gov NCT05271929. The trial was conducted in accordance with the Good Clinical Practice guidelines of the International Council of Harmonization and the principles of the Declaration of Helsinki.

Patients

A total of 120 patients were enrolled in Germany (100 patients, 7 centres), the Netherlands (11 patients, 1 centre), France (9 patients, 2 centres), all in cohort 2.

The trial initially planned to enrol patients, aged 70 years or older, or under 70 years with significant comorbidities resulting in a COVID-age of 70 years or more according to the ALAMA risk calculator in cohort 1. Since vaccination was an exclusion criterion for cohort 1, recruitment was effectively made impossible by the successful vaccination campaigns. Information on sex at birth was self-reported by study participants.

Inclusion criteria for cohort 2 were criteria for a immunocompromised population: patients with primary or acquired (lymphoid or myeloid malignancies, solid tumour with ongoing chemotherapy, allogenic hematopoietic stem cell transplantation, organ transplantation, immunosuppressive treatments, AIDS), immune deficiencies or patients without detectable seroconversion ≥ 3 weeks after complete vaccination schedule with an approved vaccine. Patients eligible for both cohorts were included in the immunocompromised cohort (cohort 2).

The main exclusion criteria were: age < 18 years; history of documented SARS-CoV-2 infection in the 90 days prior to enrolment; patients for whom CCP transfusion could not be completed within 7 days of symptom onset; and prior SARS-CoV-2 vaccination (only for cohort 1); and unauthorised prior or concurrent treatment for COVID-19. The following treatment options were authorized as SoC: monoclonal antibodies (e.g. Sotrovimab, Tixagevimab/Cilgavimab) and antivirals (Nirmatrelvir/Ritonavir, Molnupiravir and Remdesivir).

Randomisation

Eligible patients were randomised using a central web-based randomisation service (CleanWeb, Telemedicine Technologies; Boulogne Billancourt, France). Patients were allocated based on a pre-specified randomisation list, separated for each patient population (cohort 1 and 2). Randomisation was performed at a 1:1 ratio, blocked (with randomly varying block sizes of two and four) and stratified by country. Unless otherwise stated, the time of enrolment is given as an interval (in days) since the onset of symptoms.

Sample size calculation

We estimated that the risk of severe COVID-19 in the vulnerable population would be 30% and that the intervention would reduce the risk of hospitalisation by 50%.[2] Based on a Z-Test, a two-tailed α of 0.05, a 1-β of 0.90, and a relative risk of 0.5, 316 patients are required. The sample size was increased to 340 to account for missing data/loss to follow up. Because the sample size calculation was done before vaccines became largely available and the actual risk of severe COVID-19 was evolving rapidly, an interim analysis with the objective to increase the sample size was performed after enrolment of 102 patients.[1] The conditional power was below the threshold for sample size increase set at 50% and no adjustment to the sample size was performed.

Early trial termination

In accordance with the criteria of the study protocol, the study was terminated prematurely on January 18, 2024, before the planned number of patients had been reached. Due to the significant decline in the number of newly diagnosed infections and the difficulties in opening new centres in the outgoing pandemic, recruitment declined. The Data Safety Monitoring Board recommended to stop the trial.

Outcome measures

Adjudication of the primary endpoint

The primary endpoint is the proportion of participants with (1) at least one overnight stay in hospital for progressive COVID-19 symptoms, or (2) who died, by day 28 after randomisation.

According to the trial protocol, COVID-19 related hospitalisations were adjudicated by a three-member panel. Each member independently came to a decision as to whether or not the hospitalisation or the decision to extend hospitalisation was related to COVID-19, using as much information as available, such as hospital discharge forms, but remained blinded for the randomisation group. The final classification of whether the hospitalization was due to COVID-19 or not, was made by majority decision of the panel.

Complete list of secondary endpoints

Secondary endpoints included (i) proportion of participants with hospitalisation for progressive COVID-19 symptoms, or death by day 14 after randomisation (stages 4 to 10 of the WHO scale); (ii) proportion of participants with hospitalisation for progressive COVID-19 symptoms requiring O_2 support, or death by day 14 and 28 after randomisation (stages 5 to 10 of the WHO scale); (iii) all-cause mortality by day 28, 90 and 180 after randomisation; (iv) proportion of patients with supplemental oxygen by day 14 and 28 after randomisation; (v) proportion of patients with non-invasive ventilation by day 14 and 28 after randomisation; (vi) proportion of patients with intubation and mechanical ventilation by day 14 and 28 after randomisation; (vii) change in 10-point WHO Clinical Progression Scale score by day 14 and 28 after randomisation; (viii) duration of hospital admission censored at 28 days after randomisation (for participants reaching primary endpoint); (ix) proportion of patients with admission to ITU by day 14 and 28 after randomisation, (x) duration of ITU admission censored at 28 days after randomisation; (xi) proportion of patients with long COVID-19 symptoms and time to recovery assessed by Post-COVID-19 functional status scale (PCFS) and COVID-19 Yorkshire Rehabilitation Scale (C19-YRS) questionnaires at days 28 and 180 post randomisation (xii) health-related quality of life assessed using the EQ-5D-5L at 28 and 180 days after randomisation (xiii) Assessment of O_2 support requirement based on O_2 saturation level on room air ≤ 93 % and/or respiratory rate > 30.

The PCFS was used as a tool to measure functional status over time after COVID-19 [3]. The scores of this ordinal scale are: PCFS score 0 (no limitations): no symptoms related to the COVID-19 infection. PCFS score 1 (negligible limitations): Patient can perform all usual duties/activities, although still has persistent symptoms e.g. cough, loss of taste/smell. PCFS score 2 (slight limitations): Patient occasionally needs to avoid or reduce usual duties/activities/work or needs to spread these over time due to symptoms and may require occasional assistance to complete activities due to persistent symptoms e.g. minor headache/fatigue, muscle aches. PCFS score 3 (moderate limitations): patient unable to perform all usual duties/activities/work due to symptoms e.g. chest pain, moderate fatigue/brain fog, nerve pain. PCFS score 4 (severe limitations): patient unable to take care of oneself, is dependent on nursing care and/or assistance from another person due to symptoms e.g. shortness of breath, severe fatigue/brain fog.[3]

The C-19 YRS is a validated patient-reported outcome measure capturing symptom severity, functional disability, and overall health state. It was used to evaluate symptoms of long COVID-19 symptoms. The scale was used with kind licence by the University of Leeds.[4]

Exploratory Analysis

Exploratory endpoints include (i) the change in SARS-CoV-2 RNA level (polymerase chain reaction, Cycle Threshold (CT) value) in oral or nose/throat swab samples at days 3, 14, 28 and hospitalisation; (ii) change in anti-SARS-CoV-2 antibody levels in blood at days 3, 14, 28 and hospitalisation after randomisation; (ii) SARS-CoV-2 whole-genome sequence analysis in oral or nose/throat swab samples during follow up; (iii) virus sequence variation and cultivability over time, overall and in individuals receiving vs. not receiving CCP.

Safety Analysis

Safety analysis includes the number of serious Adverse Events (AE)(Grade 3/4 adverse events and AE unexpected for their nature, onset, evolution, severity or frequency) and arterial and venous thromboembolic events at 28, 90 and 180 days after randomisation.

CCP transfusions and standard treatment

CCP was obtained by apheresis from donors who had recovered from COVID-19 infection (at least 14 days after recovery) and had been vaccinated (irrespective of the order of infection and vaccinations). The donors had to fulfil all criteria for plasma donation according to national regulations.

Two ABO-compatible CCP units (200 – 350 mL each) were administered in addition to the standard of care within 7 days of symptom onset. CCP units transfused to patients included in France underwent pathogen reduction (Intercept Blood System, Cerus, Concord, CA). As far as availability allowed, the two CCP units for a patient should had been donated by two different donors. Plasma contained a minimum neutralising antibody

titer of 1:640 against delta (B1.617.2), Omicron (B1.1.529), or any future SARS-CoV-2 variant, or an anti-SARS-CoV-2 antibody concentration ≥ 4.000 BAU/ml measured by the QuantiVac anti-SARS-CoV-2 IgG ELISA (Euroimmun, Lübeck, Germany, cat. no. EI 2606-9601-10 G) or ≥ 20.000 IU/ml measured by the anti-SARS-CoV-2 Elecsys test (Roche, Mannheim, Germany, cat. no. 09 289 275 190).

Unless otherwise stated, the time of CCP transfusion is given as an interval (in days) since the onset of symptoms.

The following COVID-19 medications were authorised as standard of care in patients enrolled in the study as pre-exposure prophylaxis, post-exposure prophylaxis, as well as early treatment: anti-SARS-CoV-2 monoclonal antibodies (including Casirivimab/Imdevimab, Regdanvimab, Sotrovimab and Tixagevimab/Cilgavimab) and antiviral drugs (Molnupiravir, Nirmatrelvir/Ritonavir and Remdesivir).

Quantification of anti-SARS-CoV-2 antibodies and neutralisation assays

Serum samples of CCP donors and trial patients were analysed by two commercially available assays according to the instructions of the manufacturer (anti-SARS-CoV-2-QuantiVac-ELISA (IgG), Euroimmun and Elecsys Anti-SARS-CoV-2 S, Roche).

[5]Production of rhabdoviral pseudotypes has been previously described.[5] In brief, 293T cells (ATCC no. CRL-3216, RRID: CVCL_0063) were transfected with expression plasmids encoding SARS-CoV-2 spike variants B.1 [6], BA.1 [7], BA.2 [8], or BA.5 [9], BQ.1.1 [10], XBB.1.5 [11], BA.2.86 and EG.5.1 [12] (kindly provided by Stefan Pöhlmann, Infection Biology Unit, German Primate Center, Göttingen, Germany) by TransIT LT-1 (Mirus Bio LCC, cat. no. MIR 2306). One day after transfection, cells were inoculated with a replication-deficient vesicular stomatitis virus (VSV) vector in which the genetic information for its native glycoprotein (VSV-G) was replaced by genes encoding enhanced green fluorescent protein and firefly luciferase (FLuc, kindly provided by Gert Zimmer, Institute of Virology and Immunology, Mittelhäusern, Switzerland)[13], and incubated for 2 h at 37°C. Then the inoculum was removed, cells were washed with phosphate-buffered saline (PBS) and fresh medium containing anti-VSV-G antibody (I1-hybridoma cells; ATCC no. CRL-2700, RRID: CVCL_G654) was added to block remaining VSV-G carrying particles. After 16-18 h, supernatants were collected and centrifuged (2.000 x g, 10 min, room temperature) to clear cellular debris. Samples were then aliquoted and stored at -80°C.

The pseudovirus neutralisation experiments were performed as previously described.[5] In brief, Vero E6 cells were seeded in 96-well plates one day prior (6.000 cells/well, 2.5% FCS). Sera were heat-inactivated (56°C, 30 min) and serially titrated (4-fold titration series with 7 steps + buffer only control) in PBS, undiluted pseudovirus stocks added (1:1, v/v) and the mixtures incubated for 30 min at 37°C before being added to cells in duplicates (final on-cell dilution of sera: 20, 80, 320, 1.280, 5.120, 20.480, 81.920-fold). After an incubation period of 16-18 h, transduction efficiency was analysed. For this, the supernatant was removed, and cells were lysed by

incubation with Cell Culture Lysis Reagent (Promega, cat. no. E1531) at room temperature. Lysates were then transferred into white 96-well plates and luciferase activity was measured using a commercially available substrate (Luciferase Assay System, Promega, cat. no. E1501) and a plate luminometer (Orion II Microplate Luminometer, Berthold). For analysis of raw values (relative luminescence units per s, RLU/s), background signal of untreated cells was subtracted and values normalized to cells inoculated with pseudovirus preincubated with PBS only. Results are given as serum dilution on cell resulting in 50% pseudovirus neutralization (NT50), calculated by nonlinear regression ([Inhibitor] vs. normalized response – variable slope) in GraphPad Prism Version 9.1.1. For quantitative analyses, NT50 values <20 were set to a value of 10. Neutralisation capacity of transfused CCP was also assessed using GenScript surrogate neutralisation test against wild type (GenScript Biotech (Piscataway, NJ, USA), cat. no. L00847-C) and Omicron (GenScript Biotech (Piscataway, NJ, USA), cat. no L00847-C + Z03730-2).

SARS-CoV-2 PCR

SARS-CoV-2 in nasopharyngeal specimen was measured by PCR and results were expressed as cycle threshold values (CT values). CT values were measured in PCR against two different target regions: ORF-1 and pan-sarbeco. Change versus baseline was calculated as difference between the CT-values at the respective follow-up visit and the baseline value (Δ from baseline) of the individual patients. Positive values indicate an increase of CT values, i.e. a reduction of viral load. Negative values indicate a decrease of CT values, i.e. increased viral load compared to baseline.

Sequencing of the SARS-CoV-2

For SARS-CoV-2 Whole Genome Sequencing (WGS) viral RNA from nasopharyngeal swabs was isolated using RNAClean XP Beads (Beckman Coulter, cat. no.: A63987) in conjunction with isolation buffer (20 % PEG 8000 [Merck, cat. No. PHR2894]; 2,5 M NaCL [Invitrogen, cat. no. AM9760G]; 1 mM Tris-HCl pH 8 [Invitrogen, cat. no. AM9855G]; 0,05 % Tween 20 [Merck, cat. no. P1379]). In short 500 µl of the sample was diluted with 60 µl RNAClean XP Beads and 240 µl isolation buffer and incubated for 20 min at RT. Samples were pelleted on a magnet for 10 min and washed twice with 85% ethanol (Merck, cat. No. 32205-2.5L-M). Beads were dried 2-3 min and eluted with 10 µl nuclease-free water (IDT; New England Biolabs, cat. no. B1500A). A maximum of 8 µl of isolated RNA was mixed with 2 µl of LunaScript RT Supermix (NEB, cat. No M3010L) (2 min 20°C; 20 min 55°C; 1 min 95°C) for reverse transcription. For amplification of the SARS-CoV-2 genome, commercially available primer sets, designed by the ARTIC network group (https://artic.network/) (for coverage of the genome in 400 bp overlapping steps) and additionally the "Midnight" primer set (https://www.protocols.io/view/34-midnight-34-sars-cov2-genome-sequencing-protoc-14egn2q2yg5d/v1) (for coverage of the genome in 1200 bp

overlapping steps) were ordered from IDT.[14, 15] Primer sets were updated during the study to new versions when new viral variants emerged and led to primer failures. With the used primer sets, 4 multiplex PCRs were performed using the previously published conditions on protocols.io (available at https://www.protocols.io/view/ncov-2019-sequencing-protocol-bp2l6n26rgqe/v1; https://www.protocols.io/view/34-midnight-34-sars-cov2-genome-sequencing-protoc-14egn2q2yg5d/v1) by the respective research groups.[14] The Amplicons were purified using Ampure XP (Beckmann Coulter, cat. no. A63880) beads and the four respective PCRs per sample were pooled equimolar and used as input for nanopore library preparation with the ligation sequencing DNA kit (ONT, cat. no. SQK-LSK109) and native barcoding expansion kit (ONT, EXP-NBD104/) according to the manufacturer´s protocol. Each library was loaded either on a MinION R9 flow cell (ONT, FLO-MIN106) or a MinION R9 flongle (ONT, FLG001) and sequenced with a GridION Q line device for up to 24 h. Data was super-accuracy basecalled using the Guppy software v.6.2.11 integrated in the MinKnow software v22.08.14 Q of the GridION device. The Artic SARS-CoV-2 analysis pipeline (available at https://artic.network/ncov-2019/ncov2019-bioinformatics-sop.html)[14] was executed to generate alignment files, variant data, and consensus sequences (based on the MN908947.3 SARS-CoV-2 reference genome) for 400 bp and 1200 bp amplicons separately. A minimum coverage of 20 was used for the consensus sequence, less covered regions were labelled with N. Additionally, a consensus sequence from the BAM alignment of the artic pipeline was created using the CLC Genomics Workbench v12.0. Variants of the different consensus sequences were compared, inspected and corrected manually using the BAM alignment files with the CLC Genomics Workbench (RRID: SCR_011853). For viral variant annotation corrected consensus sequences were uploaded to Nextclade (available at https://clades.nextstrain.org/)[16] and Pangolin (available at https://pangolin.cog-uk.io/)[17]. Additionally, variant annotations were retrieved from Nextclade and COVID-19 genome annotator (available at http://giorgilab.unibo.it/coronannotator/).[18]

For variant analysis and comparison of the patients, all consensus sequences of the different samples of a patient were compared. A nucleotide variant was labelled as “new” if it was not seen on D1 (baseline) but in consecutive samplings (FU1, FU2, FU3). Nucleotide variants were counted as variants when the different pipelines (ARTIC or CLC Genomics Workbench) called the variant and it was seen in at least 30% of the reads at this locus. Samples with no or only D1 sample draw or 0 % covered sequence at consecutive sample draws were not considered for nucleotide variant analysis. For samples with SC and D1 sample draw the newest sample was denoted as D1 for nucleotide variant analysis. Patients with monoclonal antibody or antiviral medication were not analysed separately because of their equal distribution between SoC and CCP cohort (no monoclonal medication 12 x SoC / 10 x CCP; antiviral medication 13 x SoC / 12 x CCP).

Further Details on statistical analysis

All efficacy analysis were performed on the modified intention to treat (mITT) population in which all patients were analysed according to the group they were allocated to regardless of trial conduct. In this population, patients enrolled by error were excluded. Continuous variables were described using medians and interquartile ranges (IQR) for all patients with available data. Categorical variables were described with frequencies and percentages with the number of missing values presented. Because of the absence of events in the intervention arm, the primary endpoint was analysed using Fisher's exact test under bilateral hypotheses, with risk difference between groups provided. The 95% confidence interval for the risk difference was computed using Newcombe's method.[19] The significance threshold was set at $p<0.05$. Predefined subgroup analyses studied the treatment effect according to sex, age (above or below the median), the use of anti-SARS-CoV-2 monoclonal antibodies, and the use of SARS-CoV-2 antivirals. Planned subgroup analyses according to SARS-CoV-2 variant and vaccination status could not be performed because most patients fell into a single category. No hypothesis tests were performed for secondary outcomes. Risk difference for binary outcomes or absolute differences for continuous outcomes with their 95% CI are provided. Secondary endpoints involving hospital durations could not be analysed because of the absence of hospital stays in the intervention group. The safety analysis was performed on 119 patients as treated.

For analysis of the course of the anti-SARS-CoV-2 antibodies in the patients, the difference between the antibody concentrations at the follow-up examinations (follow-up 1, day 3; follow-up 2, day 14; and follow-up 3, day 28) and the concentration before the start of therapy (baseline) was calculated for each individual patient and each follow-up time point (Δ from baseline values). For each follow visit, the Δ from baseline between the SoC group and the CCP group was compared by Kruskal-Wallis test followed by Dunn´s test for correction of multiple comparisons. Results of SARS-CoV-2 antibody measurements and neutralization capacity are presented by medians and interquartile ranges (Fig. 3, Fig. S5, Fig. S8, Fig. S9) and geometric means (Table S8 and Figure legends). For calculation of geometric mean negative values or zero were substituted by 1.

For the SARS-CoV-2 sequencing results, the count of new acquired mutations during follow-up compared to baseline and the frequencies of different mutation types of new variants during follow up were compared between the SoC group and the CCP group by Kruskal-Wallis test followed by Dunn's test for correction of multiple comparison. The proportion of patients with still detectable SARS-CoV-2 and new mutations in the SoC and CCP group were compared using Fisher's exact test.

Statistical analysis was performed using R software v4.4.1 (R Core Team, R Foundation for Statistical Computing Vienna Austria) with the following packages: gtsummary v 2.0.1 (Sjoberg DD), UpSetR v1.4.0 (Conway J & Gehlenborg N), forestploter v1.1.2 (Dayimu A), fmsb v0.7.6 (Nakazawa m) and ggplot2 v3.5.1 (Wickham H et al.)

as well as with GraphPad Prism v9.0.2 (GraphPad Software, Boston, MA, USA). The analysis code is available on GitHub (https://github.com/mxdsmrts/covic-19). The DOI is https://doi.org/10.5281/zenodo.14790336.

SUPPLEMENTARY RESULTS

Patients

A total of 120 patients were enrolled between 11th April 2022 and 27th November 2023 in cohort 2 (**Fig 1**). Three patients were excluded, two patients in the CCP group due to withdrawal of informed consent after signature, one patient in the SoC group because he was already hospitalised for COVID-19 at time of enrolment. A total of 117 patients were included in the modified intention to treat analysis (mITT), 59 patients in the CCP arm and 58 patients in the SoC arm. Median follow-up was 182 days (IQR 180-187) and 180 days (IQR 179-184) in the CCP group and the SoC group, resp.

Baseline demographics and clinical characteristics are shown in **Table 1**. The majority of patients were male (58.1%). The median age was 57 years (IQR 44-65). All patients were immunocompromised; the most common reasons for immune deficiency were organ transplantation (75.2%) and lymphoid or myeloid haematological malignancies (12.0% and 6.8%, resp., **Table 1, Fig. S1** and **Fig. S2**). A majority of patients had a coexisting condition at entry into the trial. The most frequent comorbidities were hypertension (85.5%), chronic kidney disease (78.6%), chronic cardiac disease (24.8%) and malignant neoplasms (21.4%, **Table 1**).

SARS-CoV-2 infection

The median time from symptom onset of the current SARS-CoV-2 infection to randomisation was 3 days (IQR 2-4, **Fig.** S3). The most common symptoms were cough (87.2%), fatigue (64.1%), headache (60.7%), and rhinorrhoea (59.0%), sore throat (52.1%) and fever (44.4%, **Table S1**).

All SARS-CoV-2 infections were confirmed by a PCR from nasopharyngeal swab. PCR was still positive in 98.3% of patients at baseline. All infections in which the virus lineage could be identified were caused by Omicron B.1.1.529, whereby BA.2, BA.5 and XBB were identified in 24.8%, 34.2% and 15.4% of cases, respectively (**Table 1).** The predominant SARS-CoV-2 variant changed during the recording period and reflected the predominant variant in the population at that time **Fig. S4**).

Four patients in the CCP group and seven patients in the control group had past SARS-CoV-2 infections more than 90 days prior to enrolment (**Table 1**).

SARS-CoV-2 immunity at enrolment

The study population was intensively vaccinated. 89.8% of patients in the CCP group and 89.7% in the SoC group had received at least three vaccine doses, and 65.5% and 66.1% had received four doses, respectively (**Table 1, Table S3, Fig. S1**). The median interval between the last vaccination and inclusion in the study was 218 days (IQR 176-329) and 240 days (IQR 144-366) in the CCP group and SoC group, resp.. Forty percent of those vaccinated received a homologous vaccination regimen, i.e. all vaccination shots were given with the vaccine of one manufacturer. 84.6% of the vaccine doses for which the vaccine type was known were given with mRNA vaccines (either Tozinameran, Comirnaty®, 68.1% or Elasomeran, Spikevax®, 16.5%) (**Table S3**). 93% of the last vaccinations with known vaccination date were performed before bivalent vaccines became available in Europe after Sept 01, 2022.

Baseline levels of anti-SARS-CoV-2 antibodies measured by different assays are presented in **Fig. S5**. Taking all assays into account, patients had low antibody levels at enrolment given their SARS-CoV-2 vaccination status (e.g. median concentration of anti-SARS-CoV-2 antibody measured by QuantiVac ELISA (IgG) was 149.1 BAU/ml (IQR 36.9-592.9, **Fig. S5a**) and the concentration measured by Elecsys was 742 IU/ml (IQR 131.4-2,868, **Fig. S5b**)). While the GenScript Neutralization Assay demonstrated neutralizing activity against wild type (median 73.9%, IQR 37.5%- 94.2%; **Fig. S5c**), the neutralisation of Omicron in this assay was poor (median 3.5%, IQR 0%-11.3%; **Fig. S5d**) and also the NT50 in pseudovirus neutralization assays were low (**Fig. S5e to h**). None of the assays showed a significant difference of the baseline antibody levels between the SoC and CCP groups (**Fig. S5a to f**).

Overall, the CCP group and SoC groups were similar in terms of demographic characteristics, underlying immune deficiency, comorbidity, SARS-CoV-2 variant causing the current infection, symptoms, SARS-CoV-2 vaccination status and immune response to vaccination.

Study Treatment

Patients in both arms could receive monoclonal antibodies and antiviral drugs according to national recommendations and local availability, which were also authorized as standard therapy in the study protocol (**Table S4, Fig. S6**). All patients received two CCP units, each transfused on the same day. Median interval from symptom onset to transfusion was 4 days. The median transfused CCP volume was 559 ml (IQR 534-577, **Fig. S7**). 93.3% of patients received CCP from two different donors. 6.7 % of patients received CCP from the same donor, however, in half of these patients the CCP came from different donations (**Fig. S7**).
The transfused CCP units contained high anti-SARS-CoV-2 antibody concentrations: median IgG concentrations measured by QuantiVac ELISA (IgG) was 11,104 BAU/ml (IQR 8,453 – 12,279 BAU/ml) and 81,810 IU/ml (IQR

52,664 – 120,230 IU/ml) measured by Elecsys (**Fig. S8)**. The 50% neutralisation titres (NT50) of transfused CCP against B.1, BA.1, BA.2 and BA.5 were assessed in a pseudovirus neutralisation assay and showed a NT50 (median) of 12,158, 12,728, 14,376 and 5,110. The neutralisation capacity against the newer variants XBB.1.5, B.Q.1.1, BA.2.86 and EG5.1. had fallen significantly, with the following NT50: BQ.1. 1,116, XBB.1.5 634, BA.2.86 820, EG.5.1 759 (**Fig. S9)**.

Secondary Outcomes

Further secondary outcomes not already described in the main manuscript are summarized in **Tables S5** and **S6**. The number of events was very low overall for the endpoints requirement of supplemental oxygen, admission to intensive care unit, and non-invasive ventilation or mechanical ventilation. No difference could be calculated between the CCP and SoC groups for duration of hospitalisation, admission to the intensive care unit and ventilation, because the corresponding events did not occur in the CCP group (**Table S5**).

At day 28, the proportion of patients with a PCFS Score ≥ 2 (i.e. at least slight limitations) was 16% in the SoC group and 24% in the CCP group (difference -8.0%, 95%CI -25 to 9.0%). At day 28, the median PCFS in the SoC and the plasma groups was 0.0 and 1.0 (difference -0.19, 95%CI -0.56 to 0.18) and the median C19-YRS overall score was 7.00 and 7.00 (difference -0.02, 95%CI -0.89 to 0.85)(**Table S6**).
Health related quality of life as assessed by the EQ5D at baseline, day 28 and day 180 is shown in **Fig. S11**.

Adverse Events

Thirty-five patients (59%) in the plasma group and 42 patients (71%) in the SoC group experienced adverse events (**Table 2**). The total number of adverse events was 78 and 87 in the CCP and SoC group, resp. (**Table 2**). Only two of the AEs in the CCP group (2.6%) were considered related to the study intervention. The most frequent adverse events were fever, flu-like symptoms, urinary tract infections, lung infections and other infections (total of 17 infections, 22% of adverse events) in the CCP group and 21 infections in the SoC group, resp. **(Table S7**). Twelve patients (20%) in the CCP group and 21 patients (36%) in the SoC group experienced a total of 14 and 33 serious adverse events (**Tables 2 and S7**) none of which was considered as related to the study intervention. The most frequent serious adverse events were infections (total of 9 in the CCP group and 7 in the SoC group, **Table S7**).

Passive transfer of antibodies by CCP

We investigated the impact of the CCP transfusion on the concentration of SARS-CoV-2 antibodies in the patients' serum. To this end, we compared the change in the serum concentration of the antibodies at the time of the follow-up visits on days 3, 14 and 28 between the randomization groups relative to the baseline value on day 1 (i.e. before plasma administration in the CCP group). The SoC group show the immune response in

the vaccinated trial population without being influenced by the passively transferred antibodies in the transfused CCP. As a manifestation of this natural immune response, the anti-SARS-CoV-2 IgG concentration (measured with QuantiVac) in the control group increased by a median of 1,541 BAU/ml. However, a significantly higher increase in median antibody concentration by 1,899 BAU/ml was achieved in the CCP group (p<0.05, **Fig. 3a**). This significantly higher increase in total antibody concentration in the CCP group was also confirmed by measurements in a second system, the anti-SARS-CoV-2 Elecsys test (median increase on day 3 compared to baseline by 1,423 U/ml in the SoC vs. 8,374 U/ml in the CCP, p<0.001; **Fig. 3b**). Thus, on day 3 (follow-up visit 1), both the QuantiVac ELISA (**Fig. 3a**) and the Elecsys CLIA (**Fig. 3b**) showed a significantly higher increase in the CCP group compared to the plasma group. At further follow-up visits, there was no significant difference between the treatment groups (**Fig. 3a** and **3b**). In the surrogate neutralisation assay GenScript, the neutralisation of the wild type did not differ between the CCP group and the SoC group at any of the follow-up visits (**Fig. 3c**), but there was a significantly higher increase in the neutralisation of Omicron in the CCP group compared to baseline both on day 3 and on day 14, but no longer on day 28 (**Fig. 3d**).

In the GenScript surrogate neutralisation assay against wild type, there was an increase on day 3 compared to the initial values, but with no significant difference between the groups (**Fig. 3c**). In contrast, the neutralisation of Omicron increased by only 1.4 percentage points (median) in the SoC, but by 66 percentage points in the CCP group (p<0.0001; **Fig 3d**). The SoC group exhibited a continuous increase in neutralization capacity and the passively transferred antibodies in the CCP group waned over time. Thus, the difference in neutralisation of Omicron in the GenScript assay decreased on day 14 (median increase by 19.3 percentage points in SoC vs. 48.7 percentage points in CCP, p<0.01) and was no longer significantly different on day 28 (median increase by 44.5 percentage points in SoC and 46.6 percentage points in CCP; **Fig. 3d**).

In the pseudovirus neutralisation assays the NT50 values in the CCP group versus the SoC group increased significantly more on day 3 relative to baseline in the inhibition of BA.2, BA.5 (p<0.05; **Fig. 3e-f**), XBB.1.5 (p<0.01; **Fig. 3g**) and BQ.1.1 (p<0.001; **Fig. 3h**). In the pseudovirus neutralisation assays there was no significant difference between the groups at further follow-up visits (**Fig. 3e-h**). The superimmunized donors also had neutralizing activities against these new variants (**Fig. S9**). In the pseudovirus neutralisation assays, the NT50 values continued to increase in both groups over time (**Fig.3e-h**). On day 14 and 28 the difference between groups was no longer significant, most likely due to the developing immune response in the SoC group and the waning of the passively transferred antibodies. The geometric means of the change of serum SARS-CoV-2 antibody concentration and neutralization capacity on day 3, day 14 and day 28 for the SoC and CCP groups in the assays shown in **Figure 3a-h** are summarized in **Table S8**.

Viral load in nasopharyngeal swabs

The viral load in the nasopharyngeal swabs was measured by PCR. The CT values increased over time, indicating a reduction in viral load and elimination of the SARS-CoV-2. However, there was no significant difference between the CCP group and the SoC group (**Fig. S12 a and b**). The difference between the CT-values at the follow-up visits and the baseline value of the individual patients are shown in **Fig. S12 c and d**.

Viral Evolution

Whole genome sequencing was performed in all cases with virus detection by PCR and subsequent nanopore sequencing. Sequences obtained during follow-up visits were compared to baseline. The percentage of sequences covered per sample decreased over time but showed no significant differences between treatment groups (**Fig. 4a**) Follow-up samples revealed changes in viral genome sequence which were not present in baseline samples (**Fig. 4b** and **c**), but again with no difference between treatment groups, although variant proportion evolved dynamically with time (**Fig. 4d** and **e**). New sequence changes were detected in a broad range of genes (**Fig. 4f-h**), particularly in large genes as ORF1a, ORF1b and S. Interestingly, new sequence variants occurred in equal or higher proportions in S than ORF1a and ORF1b, despite a smaller gene size. However, no obvious differences were observed between the CCP group and the SoC group. Most mutations were missense or silent single nucleotide polymorphisms (SNP) (**Fig. 4i-k**), with no significant difference between SoC and CCP treatment group according to mutation type. There was a clear accumulation of new nucleotide variants at mutation sites which were mutated in D1 compared to the SARS-CoV-2 reference sequence (**Fig. 4l**). This was equally observed in SoC and CCP treatment group (**Fig. 4m**).

SUPPLEMENTARY FIGURES:

Figure S1. Patient characteristics at baseline

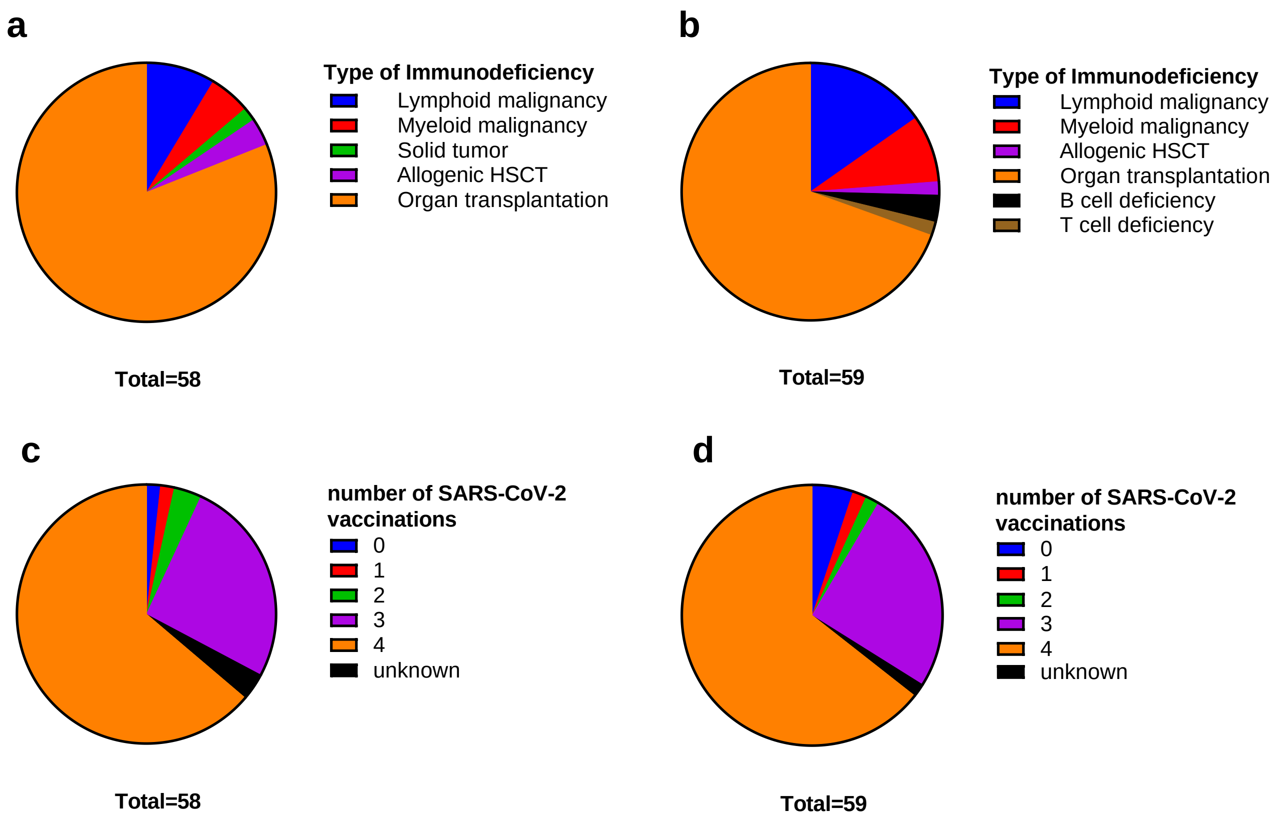

Figure S1. Patient characteristics at baseline

Proportion of patients by type of immunodeficiency in the SoC group (**a**) and the CCP group (**b**) and number of anti-SARS-CoV-2 vaccination of trial participants in the SoC group (**c**) and the CCP group (**d**).

Figure S2. Upset Plot showing the intersection of criteria of immune deficiency at baseline

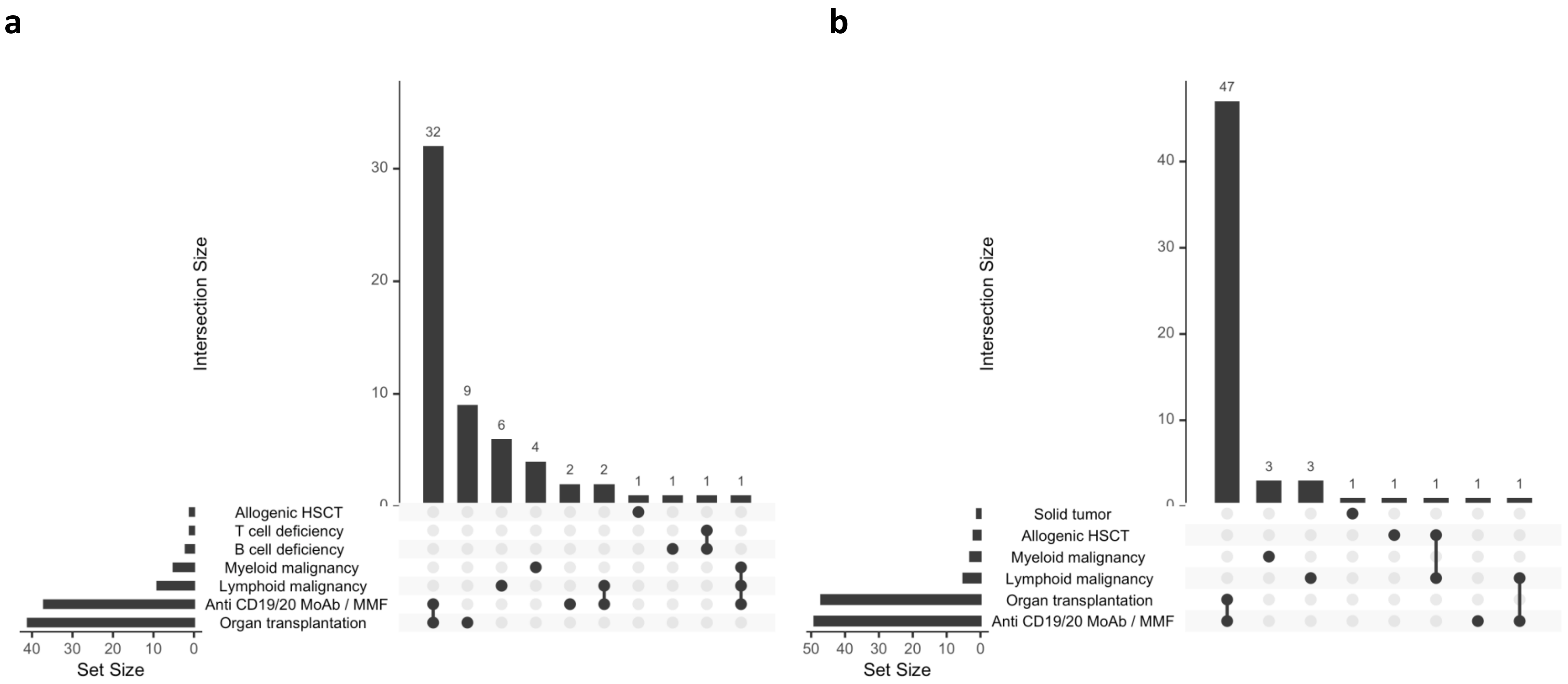

Figure S2. Upset plot showing the intersection of criteria of immune deficiency in the CCP group (a) and the SoC group (b)

Figure S3. Time from onset of symptoms to randomisation and to CCP transfusion

a **b**

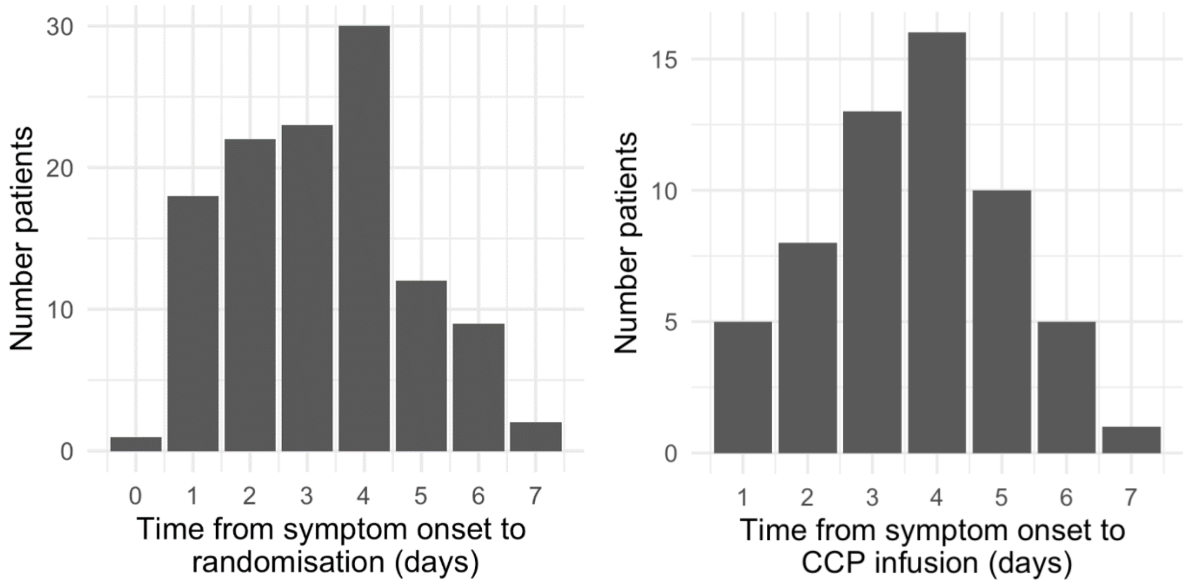

Figure S3. Time from symptom onset to randomisation (a) and to CCP transfusion (b)

The columns show the number of patients randomised at the indicated day after symptom onset (**a**; all patients) and number of patients transfused with CCP at the indicated day after symptom onset (**b**, only CCP group).

Figure S4. Enrolment of patients over the trial period

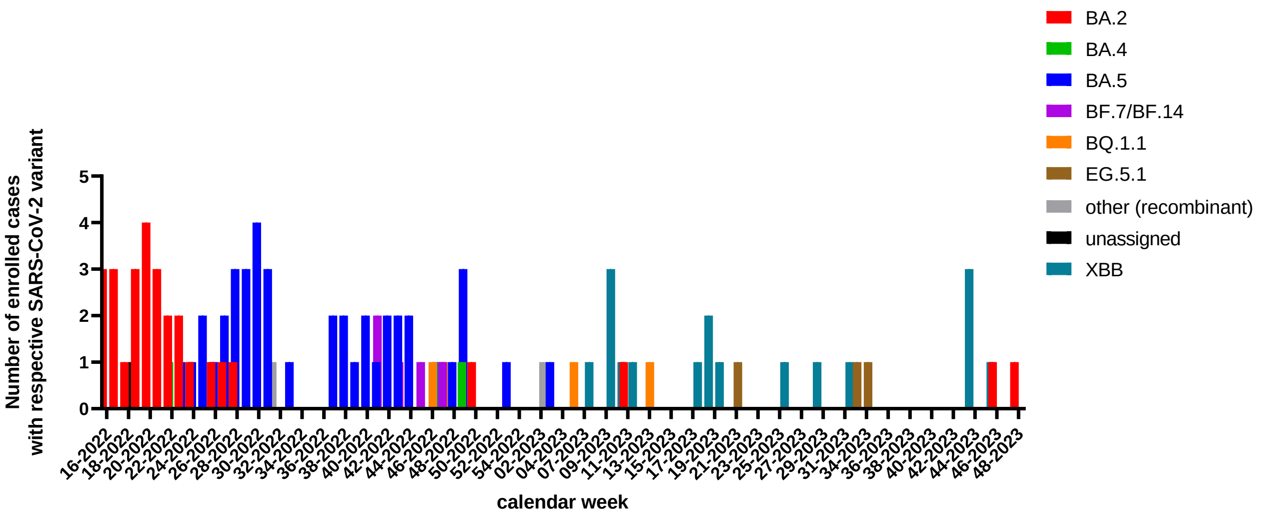

Figure S4. Enrolment of patients over the trial period by SARS-CoV-2 variant identified in the patients by time of enrolment

The columns show the number of newly enrolled patients infected by the respective SARS-CoV-2 variant.

Figure S5. Anti-SARS-CoV-2 antibody concentration at baseline by randomization group

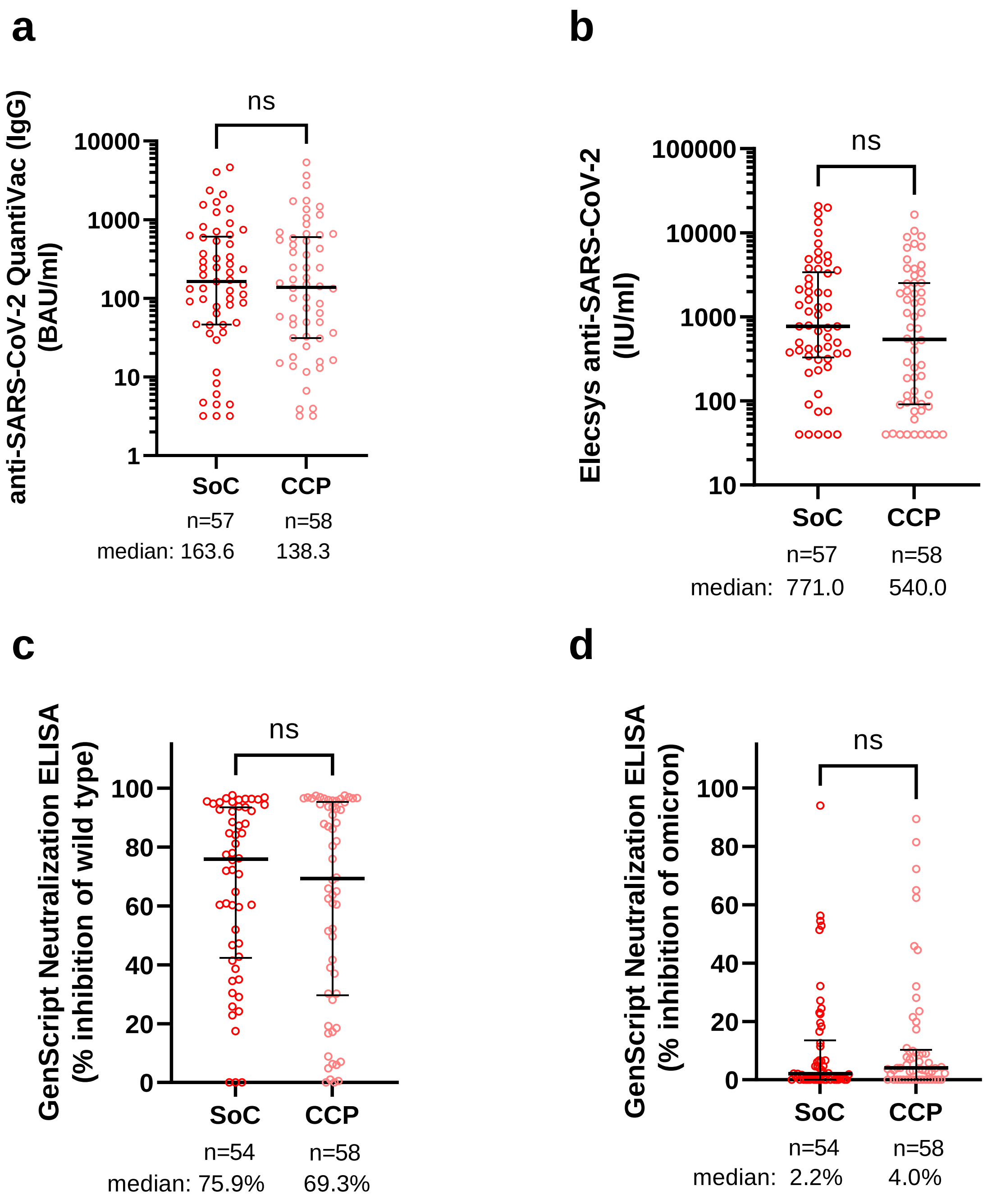

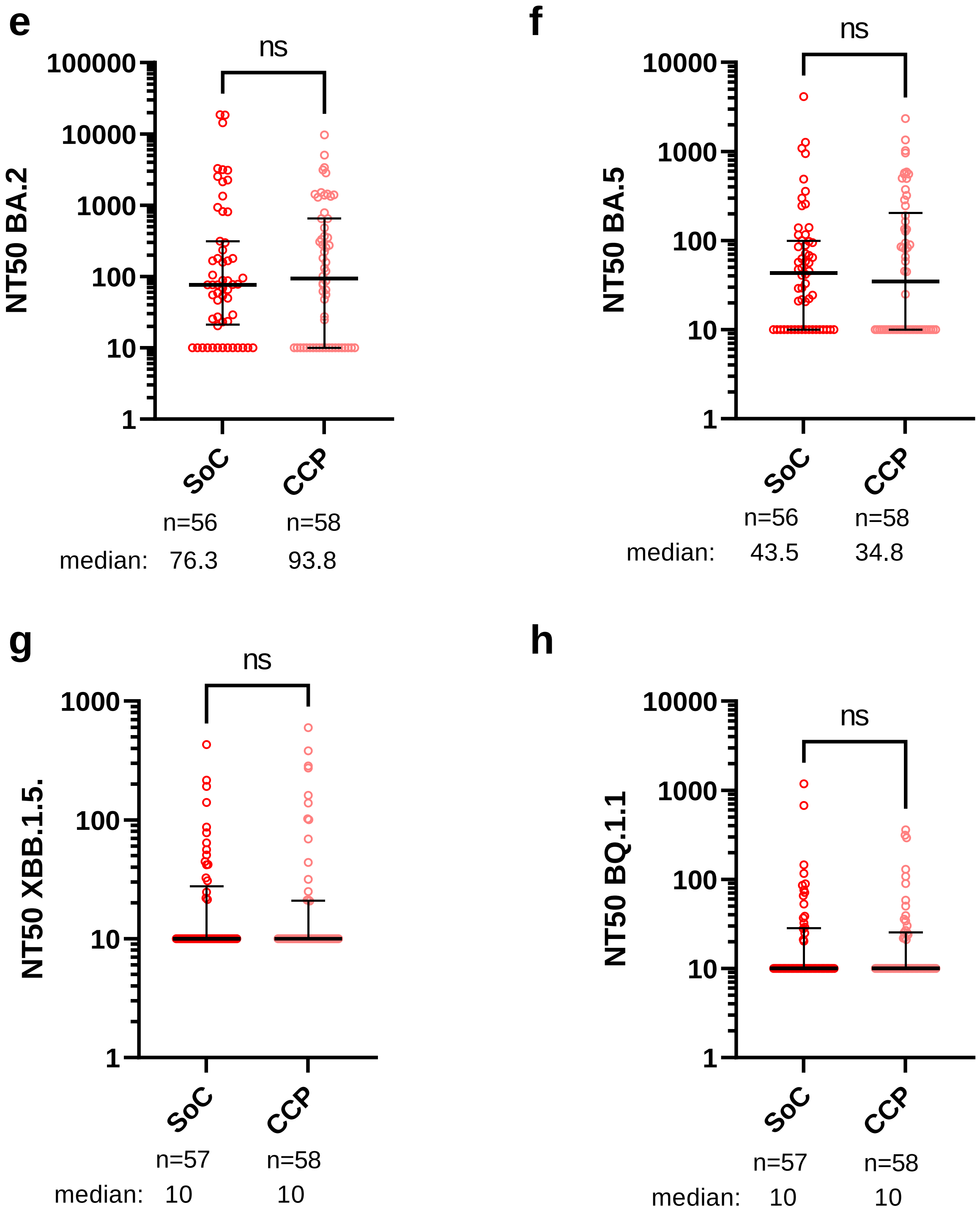
e
ns
100000
10000
1000
100
10
1
NT50 BA.2
SoC
CCP
n=56
n=58
median: 76.3
93.8
f
ns
10000
1000
100
10
1
NT50 BA.5
SoC
CCP
n=56
n=58
median: 43.5
34.8
g
ns
1000
100
10
1
NT50 XBB.1.5.
SoC
CCP
n=57
n=58
median: 10
10
h
ns
10000
1000
100
10
1
NT50 BQ.1.1
SoC
CCP
n=57
n=58
median: 10
10

Figure S5. Anti-SARS-CoV-2 antibody concentration at baseline by randomisation group

Anti-SARS-CoV-2 antibody concentration in serum of patients at baseline measured by anti-SARS-CoV-2-QuantiVac-ELISA (IgG) (**a**), Elecsys anti-SARS-CoV-2 S (**b**) or GenScript Surrogate Neutralisation Test against wild type (**c**) or Omicron (**d**) and neutralising titer (50% inhibition of pseudovirus; NT50) measured against BA.2 (**e**), BA.5 (**f**), XBB.1.5 (**g**) and BQ.1.1 (**h**). Results are shown for SoC group (light symbols) and the CCP group (bold symbols). Horizontal lines indicate the median and error bars the interquartile range. Baseline values of the SoC group and the CCP group were compared by Kruskal-Wallis test (p-values for the pairwise comparisons were $p>0.05$ (ns; not significant). The geometric means of the antibody concentration in the SoC and CCP groups were 140 and 126 BAU/ml in the anti-SARS-CoV-2 QuantiVac ELISA (IgG) (**a**), 865 U/ml and 527 U/ml in Elecsys Anti-SARS-CoV-2 S assay (**b**), 51.2% and 39.6% in the GenScript Surrogate Neutralisation assay against wild type (**c**), 3.4% and 4.9% in the GenScript Neutralisation assay against Omicron (**d**), titres 111 and 106 (NT50) in the pseudovirus neutralisation assay against BA.2(**e**), titres 47 and 48 against BA.5 (**f**), titres 17 and 17 against XBB.1.5 (**g**), and titres 18 and 17 against BQ.1.1(**h**).

Figure S6. Upset Plot of COVID-19 medications (monoclonal antibodies and antivirals)

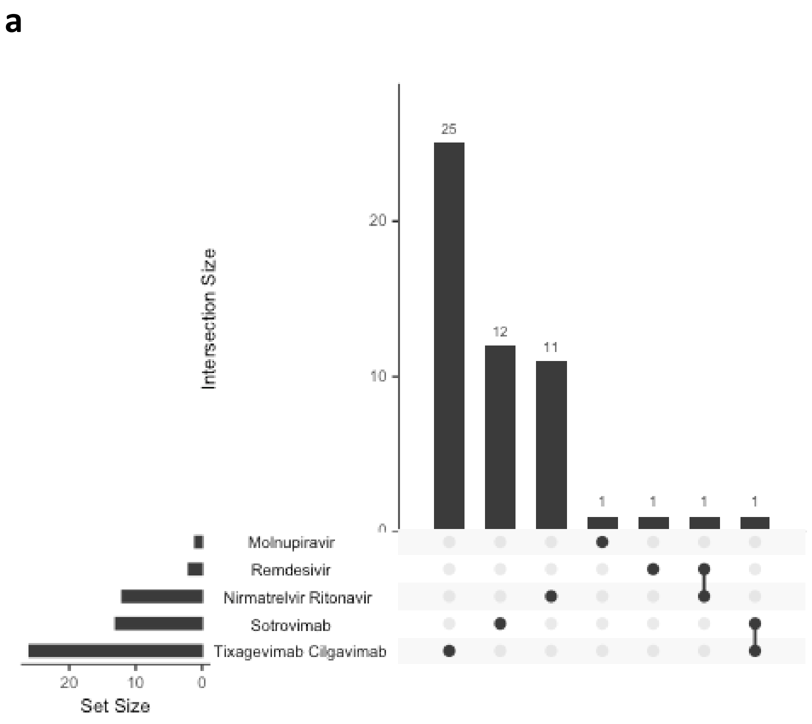

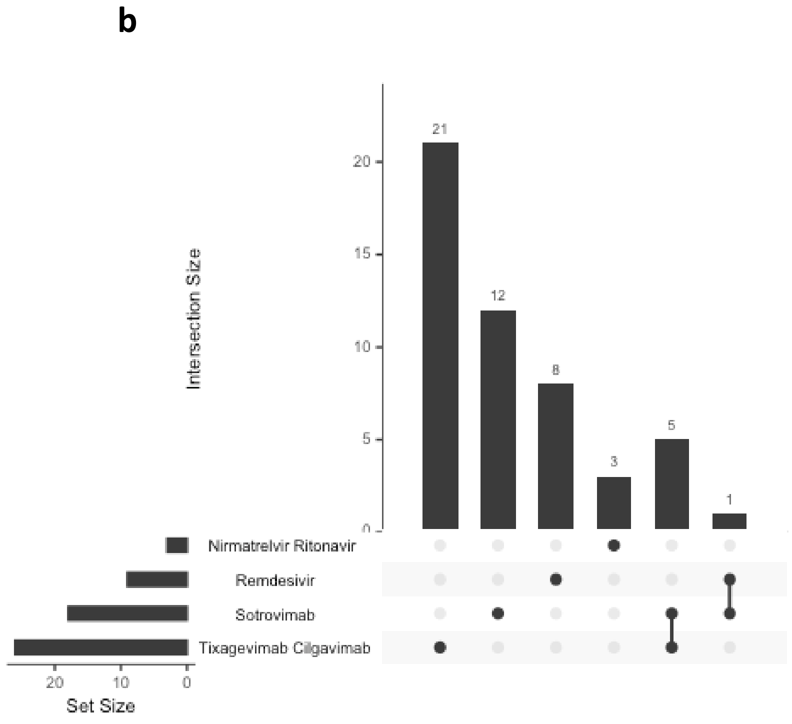

Figure S6. Upset Plot of COVID-19 medications (monoclonal antibodies and antivirals) in the CCP group (a) and the SoC group (b)

Figure S7. Characteristics of transfused CCP units

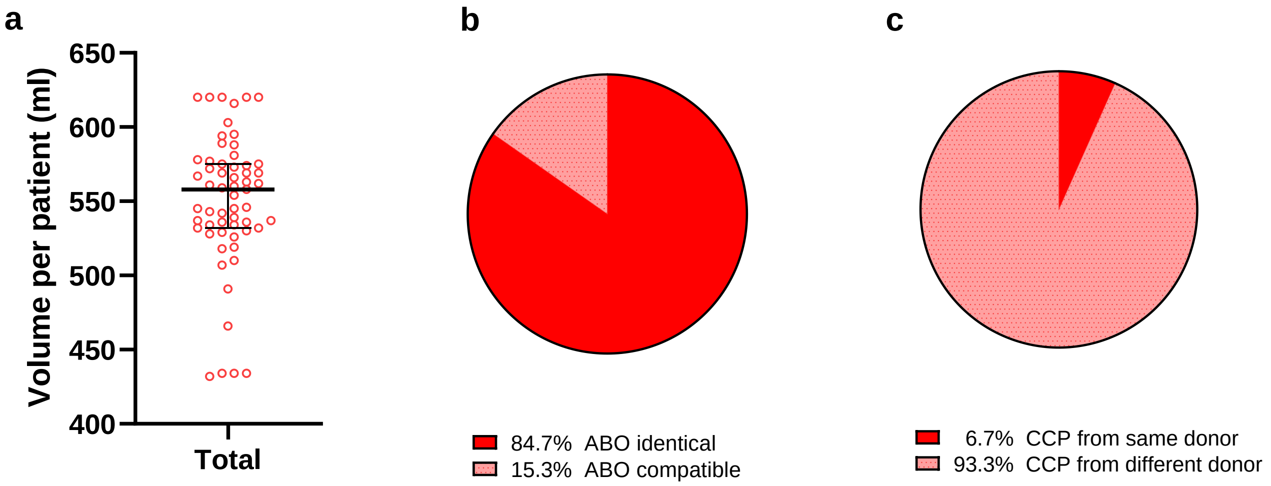

Figure S7. Characteristics of transfused CCP units

The characteristics of CCP are shown in terms of (**a**) total CCP volume per patient (in ml), (**b**) ABO matching (ABO identical [dark red] or ABO compatible [light red] and (**c**) source of the two CCP units from either two different donors (light red) or the same donor (dark red). Horizontal lines indicate the median and error bars the interquartile range.

Figure S8. Anti-SARS-CoV-2 antibody concentration in transfused CCP units

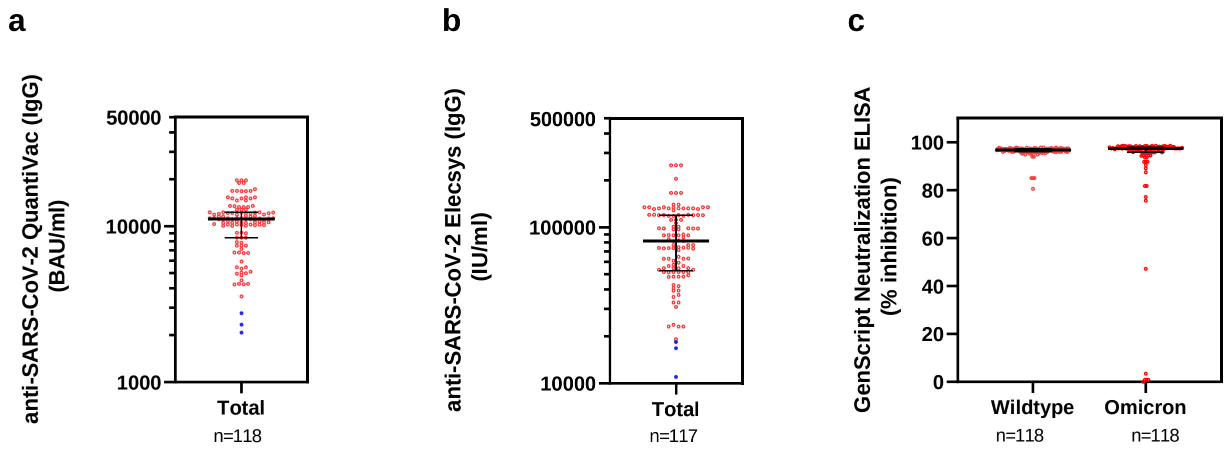

Figure S8. Anti-SARS-CoV-2 antibody concentration in transfused CCP units

Anti-SARS-CoV-2 antibody concentrations of transfused CCP were measured by anti-SARS-CoV-2-QuantiVac-ELISA (IgG) (**a**), Elecsys Anti-SARS-CoV-2 S (**b**) and GenScript Surrogate Neutralization Test against wild type and Omicron (**c**). CCPs below the required antibody concentration (blue circles) of 4,000 BAU/ml in the QuantiVac and 20,000 IU/ml do not represent a violation of the study protocol, as in one study centre the CCPs were selected according to the criterion of a titre > 1:640 in a plaque-reduction neutralisation assay (PRNT). Samples had PRNT titres against BA.5 of 1:3,519, 1:2,422, and 1:8,954. Neutralisation capacity of transfused CCP was assessed using GenScript surrogate neutralisation test (**c**) against wild type and Omicron. Horizontal lines indicate the median and error bars the interquartile range. The geometric means were 9,909 BAU/ml in the anti-SARS-CoV-2-QuantiVac-ELISA (IgG) (**a**), 76,064 U/ml in the Elecsys Anti-SARS-CoV-2 S assay (**b**), 96.1% and 79.3% in the GenScript Surrogate Neutralization Assay against wild type and Omicron, resp. (**c**).

Figure S9. Neutralising titers against SARS-CoV-2 in transfused CCP

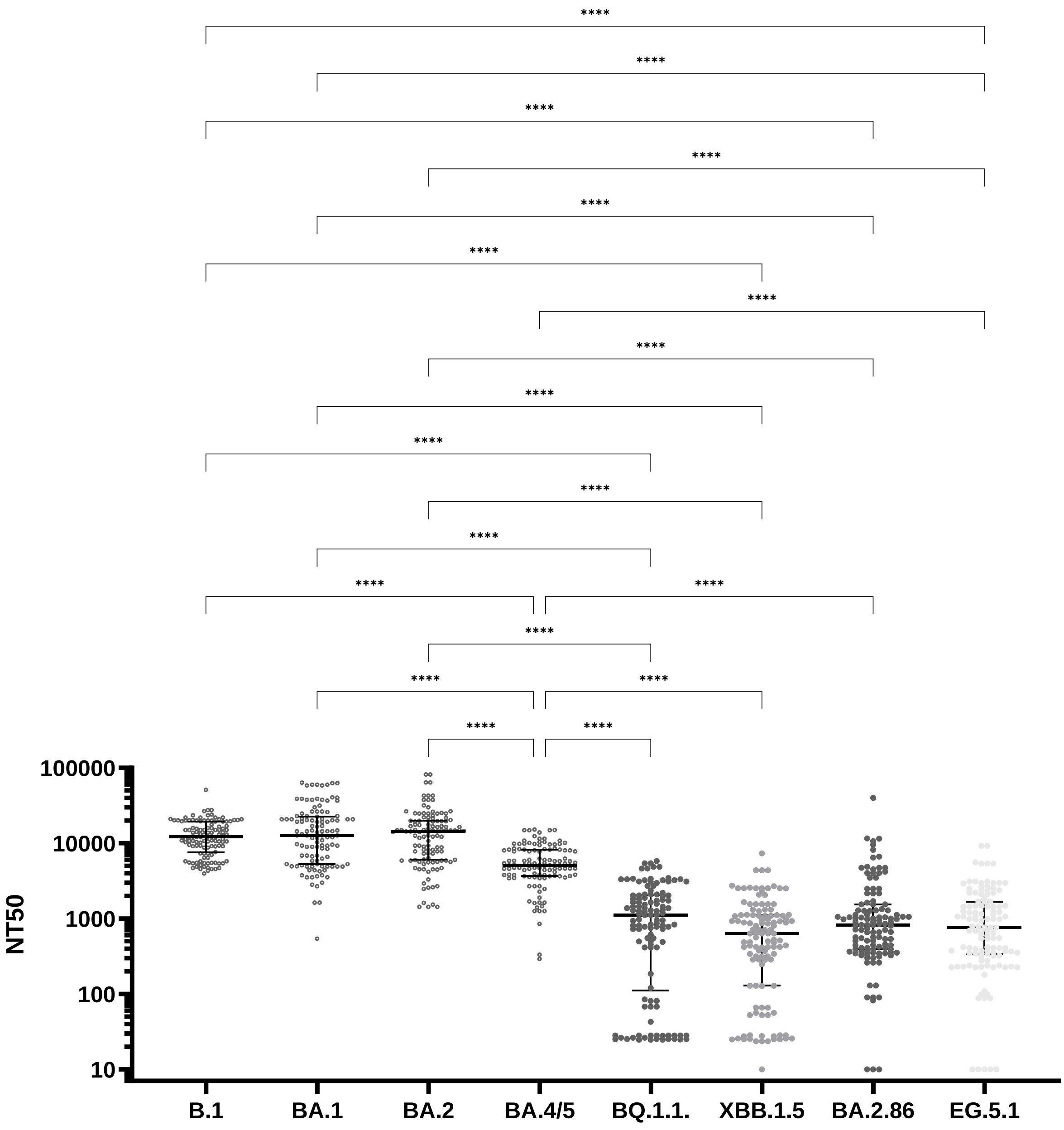

Figure S9. Neutralising titers against SARS-CoV-2 in transfused CCP

NT50 titres of total and transfused CCP (n=118) were determined using pseudovirus neutralisation assays. The p-values for the pairwise comparisons were calculated using the Kruskal-Wallis test followed by Dunn's test to correct for multiple comparisons (*p<0.5, ****p<0.0005). Horizontal lines indicate the median and error bars the interquartile range. The geometric mean of NT50 against the variants were 11,588 (B.1), 12,180 (BA.1), 11,621 (BA.2), 4,956 (BA.4/5), 568 (BQ.1.1), 410 (XBB.1.5), 834 (BA.2.86) and 675 (EG.5.1).

Figure S10. SARS-CoV-2 variants which caused infection in donors and time between donation and transfusion of CCP.

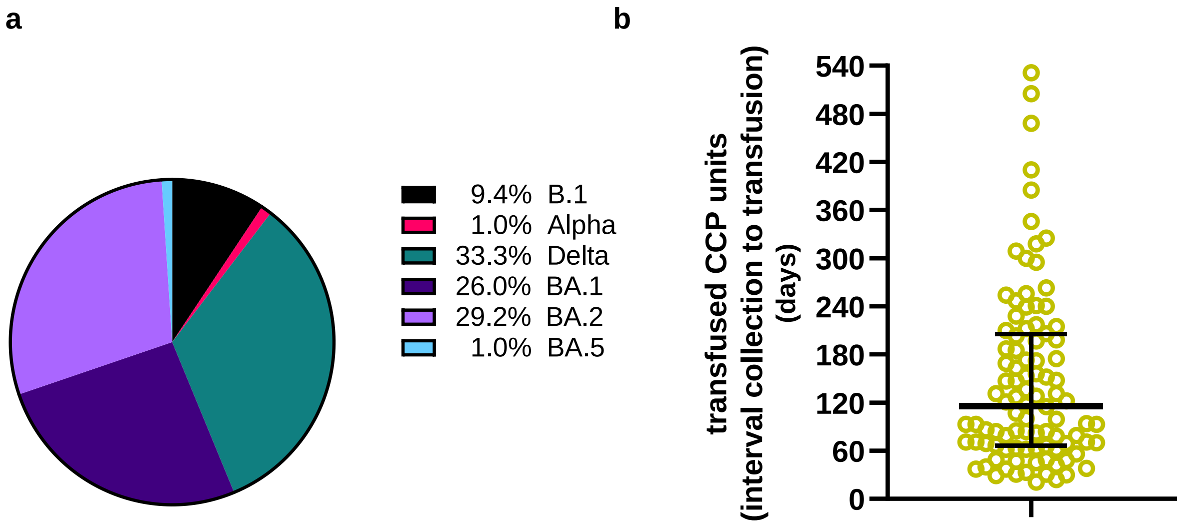

Figure S10. SARS-CoV-2 variants which caused the infection in donors and the time between donation and transfusion of CCP

The pie chart (**a**) shows the SARS-CoV-2 variant which most likely had caused the infection. The date of infection of the donors is known and we assumed that the variant which was most prevalent in the general population according to surveillance data at that time most likely also caused the infection in the donor. The variant that caused the infection in the donors has not been confirmed by molecular virus testing.

The scatter plot (**b**) shows the interval from collection to transfusion of CCP units. The horizontal line represents the median and the error bars the interquartile range.

Figure S11. Health-related quality of life dimensions

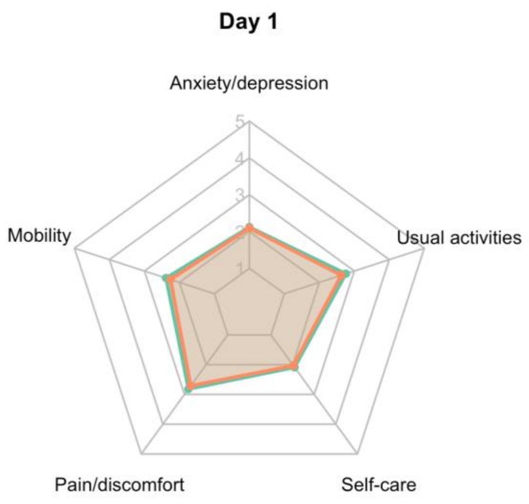

Figure S11. Health-related quality of life dimensions

The dimensions anxiety/depression, usual activities, self-care, pain/discomfort and mobility were assessed by EQ-5D-5L at day 1, day 28 and day 180.

Figure S12. Detection of SARS-CoV-2 in nasopharyngeal swabs from patients

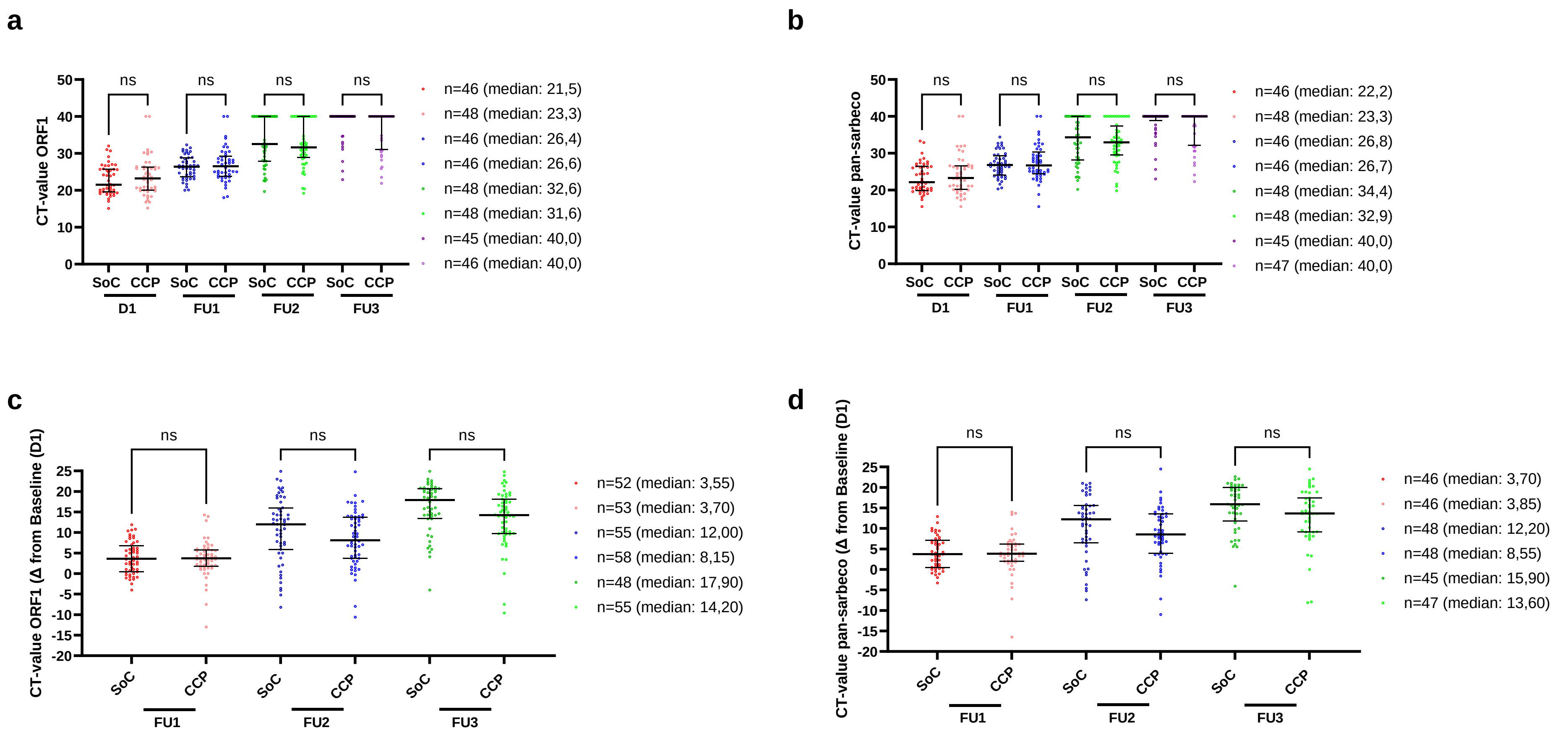

Figure S12. Detection of SARS-CoV-2 in nasopharyngeal swabs from patients on day 3, day 14 and day 28

Cycle threshold values (CT-values) in nasopharyngeal swabs of patients at baseline, follow up visit 1 (FU1, day 3; red symbols), visit 2 (FU2, day 14; blue symbols) and visit 3 (FU3, day 28; green symbols) measured in PCR against two different target regions (ORF-1)(**a**) and pan-sarbeco (**b**). Results are shown for the SoC group (light symbols) and the CCP group (bold symbols). The change of the CT-values in nasopharyngeal swabs of patients on follow up visit 1 (FU1, day 3; red symbols), visit 2 (FU2, day 14; blue symbols) and visit 3 (FU3, day 28; green symbols) compared to baseline measured in PCR against ORF-1 (**c**) and pan-sarbeco (**d**). The values in panels **c** and **d** show the difference between the CT-values at the respective follow-up visit and the baseline value (Δ from baseline) of the individual patients. Positive values indicate an increase of CT values, i.e. a reduction of viral load. Negative values indicate a decrease of CT values, i.e. increased viral load compared to baseline. Horizontal lines indicate the mean and error bars the interquartile range. For each visit, the absolute CT values (panels **a** and **b**) or the Δ from baseline (panels **c** and **d**) between the SoC group and the CCP group was compared by Kruskal-Wallis test followed by Dunn´s test for correction of multiple comparisons. The p-values for all pairwise comparisons were $p>0.05$ (ns; not significant).

SUPPLEMENTARY TABLES

Table S1. Concomitant corticosteroids and immunosuppressive drugs at enrolment

	Standard Care<br>N = 58	CCP<br>N = 59
Corticosteroids	35 (60.3%)	32 (54.2%)
**Immunosuppressive medication**		
Any	41 (70.7%)	38 (64.4)
Tacrolimus	35 (60.3%)	26 (44.1%)
Mycophenolate Mofetil	35 (60.3%)	32 (54.2%)
Ciclosporin	4 (6.9%)	6 (10.2%)
Azathioprin	1 (1.7%)	2 (3.4%)
Belatacept	2 (3.4%)	6 (10.2%)
Everolimus	0 (0.0%)	1 (1.7%)
Rituximab	1 (1.7%)	1 (1.7%)

Table S2. Most common COVID-19 symptoms at enrolment*.

Symptom	Standard Care N=58	CCP N=59
Fever	26 (44.8%)	26 (44.8%)
Cough	49 (84.5%)	53 (89.8%)
Cough with sputum	19 (32.8%)	18 (30.5%)
Sore throat	28 (48.3%)	33 (55.9%)
Loss of smell	3 (5.2%)	8 (13.6%)
Loss of taste	6 (10.3%)	6 (10.2%)
Rhinorrhoea	34 (68.6%)	35 (59.3%)
Wheezing	5 (8.6%)	2 (3.4%)
Chest pain	7 (12.1%)	6 (10.2%)
Myalgia	18 (31.0%)	19 (32.2%)
Arthralgia	19 (32.8%)	17 (28.8%)
Fatigue/Malaise/Dizziness	36 (62.1%)	39 (66.1%)
Dyspnoea	10 (17.2%)	14 (23.7%)
Inability to walk	2 (3.4%)	3 (5.1%)
Headache	37 (63.8%)	34 (57.6%)
Lymphadenopathy	3 (5.2%)	2 (3.4%)
Other	5 (8.6%)	9 (15.3%)

**Only symptoms which were reported in at least 5% of patients in at least one group are listed.*

Table S3. SARS-CoV-2 vaccination of trial patients – type of vaccine

Characteristic	Overall<br>N = 117[1]	Standard care<br>N = 58[1]	CCP<br>N = 59[1]
**Manufacturer of 1st dose**			
Pfizer/BioNTech	74 (67.9%)	37 (67.3%)	37 (68.5%)
AstraZeneca	24 (22.0%)	14 (25.5%)	10 (18.5%)
Moderna	11 (10.1%)	4 (7.3%)	7 (13.0%)
Unknown	8	3	5
**Manufacturer of 2nd dose**			
Pfizer/BioNTech	77 (72.0%)	38 (70.4%)	39 (73.6%)
AstraZeneca	17 (15.9%)	11 (20.4%)	6 (11.3%)
Moderna	13 (12.1%)	5 (9.3%)	8 (15.1%)
Unknown	10	4	6
**Manufacturer of 3rd dose**			
Pfizer/BioNTech	79 (77.5%)	41 (80.4%)	38 (74.5%)
AstraZeneca	9 (8.8%)	3 (5.9%)	6 (11.8%)
Moderna	13 (12.7%)	7 (13.7%)	6 (11.8%)
Janssen	1 (1.0%)	0 (0.0%)	1 (2.0%)
Unknown	15	7	8
**Manufacturer of 4th dose**			
Pfizer/BioNTech	39 (52.0%)	20 (54.1%)	19 (50.0%)
AstraZeneca	1 (1.3%)	1 (2.7%)	0 (0.0%)
Moderna	28 (37.3%)	13 (35.1%)	15 (39.5%)
Other	7 (9.3%)	3 (8.1%)	4 (10.5%)
Unknown	42	21	21

Table S3 (continued). SARS-CoV-2 vaccination of trial patients – time from vaccination to randomisation.

Characteristic	Overall<br>N = 117[1]	Standard care<br>N = 58[1]	CCP<br>N = 59[1]
**Time from 1st dose to randomisation (days)**			
Median (Q1, Q3)	527 days<br>(435 days, 634 days)	499 days<br>(423 days, 609 days)	534 days<br>(439 days, 644 days)
Min, Max	215 days, 1,030 days	215 days, 1,004 days	348 days, 1,030 days
Unknown	9	3	6
**Time from 2nd dose to randomisation (days)**			
Median (Q1, Q3)	485 days<br>(384 days, 590 days)	456 days<br>(381 days, 588 days)	496 days<br>(393 days, 598 days)
Min, Max	293 days, 1,002 days	293 days, 977 days	306 days, 1,002 days
Unknown	11	4	7
**Time from 3rd dose to randomisation (days)**			
Median (Q1, Q3)	330 days<br>(272 days, 451 days)	325 days<br>(281 days, 447 days)	347 days<br>(265 days, 452 days)
Min, Max	108 days, 923 days	152 days, 914 days	108 days, 923 days
Unknown	15	8	7
**Time from 4th dose to randomisation (days)**			
Median (Q1, Q3)	230 days<br>(169 days, 333 days)	239 days<br>(145 days, 362 days)	218 days<br>(176 days, 329 days)
Min, Max	44 days, 754 days	44 days, 552 days	107 days, 754 days
Unknown	44	22	22

Table S4. CCP treatment, anti-S monoclonal antibodies and antivirals

A) Number (proportion) of patients who receive anti-SARS-CoV-2 monoclonal antibodies and/or antivirals.

	Standard Care N=58	*CCP* N=59
**No. of plasma units**		
2 CCP units	–	59 (100%)
**Anti-SARS-CoV-2 monoclonal antibodies***		
Tixagevimab/Cilgavimab	26 (44.8%)	26 (44.1%)
Sotrovimab	18 (31.0%)	13 (22.0%)
**Antivirals***		
Nirmatrelvir/Ritonavir	3 (5.2%)	12 (20.3%)
Remdesivir	8 (13.8%)	2 (3.4%)
Molnupiravir	0 (0%)	1 (1.7%)

B) Timing between the administration of antivirals and monoclonal antibodies relative to the CCP transfusion (day 0) in the CCP group.

	**Molnupiravir**	**Nirmatrelvir Ritonavir**	**Remdesivir**	**Sotro-vimab**	**Tixagevimab Cilgavimab**
**Time relative to CCP (days)**	**N = 1**[1]	**N = 12**[1]	**N = 2**[1]	**N = 13**[1]	**N = 26**[1]
-4		2			1
-3		2			
-2	1	3			
-1		2		1	
0		1	2	12	25
1		1			
Unknown		1			

[1] n

*Medication given up to day 28 (evaluation of primary endpoint) are listed. Some patients received more than one SARS-CoV-2 medication. The intersection is shown in UpSet Plots in Fig. S6.

Table S5. Secondary outcomes

Characteristic	N	Standard Care<br>N = 58[1]	CCP<br>N = 59[1]	Difference[2]	95% CI[2,3]
**COVID-19 related hospitalisation or death (day 14)**	117	4 (6.9)	0 (0)	-6.9%	-16% to 0.51%
**Hospitalisation for COVID-19 w/ O2 or death (day 14)**	117	3 (5.2)	0 (0)	-5.2%	-14% to 1.8%
**Hospitalisation for COVID-19 w/ O2 or death (day 28)**	117	3 (5.2)	0 (0)	-5.2%	-14% to 1.8%
**All-cause mortality (day 28)**	117	1 (1.7)	0 (0)	-1.7%	-9.1% to 4.6%
**All-cause mortality (day 90)**	117	2 (3.4)	1 (1.7)	-1.8%	-10% to 6.0%
**All-cause mortality (day 180)**	117	2 (3.4)	1 (1.7)	-1.8%	-10% to 6.0%
**Oxygen required (day 14)**	117	2 (3.4)	0 (0)	-3.4%	-12% to 3.2%
**Oxygen required (day 28)**	117	2 (3.4)	0 (0)	-3.4%	-12% to 3.2%
**Non-invasive ventilation (day 14)**	117	1 (1.7)	0 (0)	-1.7%	-9.1% to 4.6%
**Non-invasive ventilation (day 28)**	117	1 (1.7)	0 (0)	-1.7%	-9.1% to 4.6%
**Mechanical ventilation (day 14)**	117	0 (0)	0 (0)		
**Mechanical ventilation (day 28)**	117	0 (0)	0 (0)		
**Diff. WHO scale (day 14)**	112	0.00 (-1.00 – 0.00)	0.00 (-1.00 – 0.00)	0.07	-0.35 to 0.49
Unknown		2	3		
**Diff. WHO scale (day 28)**	111	-2.00 (-2.00 – -1.00)	-2.00 (-2.00 – 0.00)	-0.31	-0.72 to 0.09
Unknown		3	3		
**ICU admission (day 14)**	117	0 (0)	0 (0)		
**ICU admission (day 28)**	117	1 (1.7)	0 (0)	-1.7%	-9.1% to 4.6%

[1]n (%); Median (IQR)

[2]2-sample test for equality of proportions with continuity correction; Welch Two Sample t-test

[3]CI = Confidence Interval

The following secondary outcomes could not be calculated since no event occurred in the CCP group: duration of hospital admission censored at 28 days after randomisation (for participants reaching primary endpoint) and duration of ITU admission censored at 28 days after randomisation.

Table S6. Secondary Outcomes: Post-COVID-Functional Scale, COVID-19 YRS and EQ5D

Characteristic	N	Standard Care N = 58[1]	CCP N = 59[1]	Difference[2]	95% CI[2,3]
**Post COVID-19 Functional Scale (PCFS)**					
**PCFS (Day 28)**	106	0.00 (0.00 – 1.00)	1.00 (0.00 – 1.00)	-0.19	-0.56 to 0.18
Unknown		7	4		
**PCFS (Day 180)**	97	0.00 (0.00 – 0.00)	0.00 (0.00 – 1.00)	-0.17	-0.47 to 0.12
Unknown		9	11		
**Diff. PCFS (day 28)**	103	-1.00 (-2.00 – 0.00)	0.00 (-1.00 – 0.00)	-0.36	-0.77 to 0.04
Unknown		9	5		
**Diff. PCFS (day 180)**	93	-1.00 (-2.00 – -1.00)	-1.00 (-2.00 – 0.00)	-0.47	-0.92 to -0.03
Unknown		12	12		
**COVID-19 YRS**					
**C19-YRS Overall Day 28**	109	7.00 (5.00 - 8.00)	7.00 (5.00 – 8.00)	-0.02	-0.89 to 0.85
**C19-YRS Functional Day 180**	92	0.5 (0.0 – 4.0)	1.0 (0.0 – 6.0)	-0.63	-2.9 to 1.7
Unknown		14	11		
**C19-YRS Overall Day 180**	96	8.00 (6.00 – 9.00)	7.00 (5.00 – 8.00)	0.57	-0.46 to 1.6
Unknown		11	10		
**Diff. C19-YRS Symptom (Day28)**	76	1 (-1 – 7)	5 (0 – 14)	-4.3	-8.7 to 0.24
Unknown		21	20		
**Diff. C19-YRS Functional (Day 28)**	86	0.0 (0.0 – 2.0)	0.0 (0.0 – 5.0)	-0.98	-3.2 to 1.3
Unknown		19	12		
**Diff. C19-YRS Overall (Day 28)**	100	0.00 (-2.00 – 1.00)	-0.50 (-2.00 – 0.00)	-0.02	-1.1 to 1.1
Unknown		8	9		
**Diff. C19-YRS Symptom (Day 180)**	67	0 (-3 – 3)	1 (0 – 6)	-3.9	-8.3 to 0.60
Unknown		26	24		
**Diff. C19-YRS Functional (Day 180)**	80	0.0 (0.0 – 2.0)	0.0 (0.0 – 2.0)	-0.58	-3.0 to 1.8
Unknown		20	17		
**Diff. C19-YRS Overall (Day 180)**	88	0.0 (-1.0 – 1.0)	0.0 (-2.0 – 1.0)	0.36	-0.96 to 1.7
Unknown		13	16		

[1]Median (IQR)

[2]Welch Two Sample t-test

[3]CI = Confidence Interval

Note: EQ5D VAS, EQ5D Visual analogue scale (from 0 = worst to 100 = best)

Table S7. Number and type of Adverse Events and Serious Adverse Events

(according to CTCAE Terminology).

	CCP group	SoC group
**Total number of adverse events**	**78**	**87**
**Adverse events**		
**CTCAE terminology**		
Abdominal infection	0 (0%)	1 (1.1%)
Abdominal pain	0 (0%)	1 (1.1%)
Acidosis	0 (0%)	1 (1.1%)
Acute kidney injury	0 (0%)	2 (2.3%)
Allergic reaction	2 (2.5%)	0 (0%)
Amnesia	0 (0%)	1 (1.1%)
Anaemia	1 (1.3%)	1 (1.1%)
Arterial thromboembolism	0 (0%)	1 (1.1%)
Arthralgia	1 (1.3%)	0 (0%)
Arthritis	1 (1.3%)	0 (0%)
Atrial fibrillation	2 (2.5%)	1 (1.1%)
Back pain	0 (0%)	2 (2.3%)
Biliary tract infection	0 (0%)	2 (2.3%)
Bladder infection	1 (1.3%)	0 (0%)
Blood and lymphatic system disorders - Other	0 (0%)	1 (1.1%)
Bronchial infection	0 (0%)	2 (2.3%)
Concentration impairment	2 (2.5%)	0 (0%)
Confusion	1 (1.3%)	0 (0%)
Cough	1 (1.3%)	1 (1.1%)
COVID-19 infection	3 (3.8%)	4 (4.6%)
Creatinine increased	1 (1.3%)	0 (0%)
Cytomegalovirus infection reactivation	1 (1.3%)	1 (1.1%)
Diarrhoea	2 (2.5%)	2 (2.3%)
Dyspnoea	0 (0%)	3 (3.4%)
Oedema face	1 (1.3%)	0 (0%)
Oedema limbs	1 (1.3%)	0 (0%)
Epistaxis	1 (1.3%)	1 (1.1%)
Epstein-Barr virus infection reactivation	1 (1.3%)	1 (1.1%)
Fall	0 (0%)	1 (1.1%)
Fatigue	1 (1.3%)	1 (1.1%)
Fever	5 (6.3%)	3 (3.4%)
Flu like symptoms	5 (6.3%)	7 (8.0%)
Fracture	0 (0%)	1 (1.1%)
Gastrointestinal disorders - Other	0 (0%)	1 (1.1%)
General disorders and administration site conditions – Other	0 (0%)	1 (1.1%)
Glaucoma	0 (0%)	1 (1.1%)
Heart failure	1 (1.3%)	0 (0%)

	CCP group	SoC group
**Adverse events**		
**CTCAE terminology**		
Hematoma	0 (0%)	2 (2.3%)
Haematuria	0 (0%)	1 (1.1%)
Hyperkalaemia	1 (1.3%)	0 (0%)
Infections and infestations - Other	4 (5.1%)	2 (2.3%)
Injury, poisoning and procedural complications - Other	0 (0%)	2 (2.3%)
Insomnia	1 (1.3%)	0 (0%)
Joint range of motion decreased lumbar spine	0 (0%)	1 (1.1%)
Kidney infection	0 (0%)	1 (1.1%)
Localized oedema	1 (1.3%)	0 (0%)
Loss of appetite	0 (0%)	1 (1.1%)
Lung infection	4 (5.1%)	1 (1.1%)
Lymph node pain	0 (0%)	1 (1.1%)
Memory impairment	1 (1.3%)	0 (0%)
Muscle cramp	1 (1.3%)	0 (0%)
Myalgia	1 (1.3%)	0 (0%)
Neoplasms benign, malignant and unspecified (incl. cysts and polyps) - Other	0 (0%)	4 (4.6%)
Nervous system disorders - Other	1 (1.3%)	0 (0%)
Neutrophil count decreased	1 (1.3%)	0 (0%)
Non-Arteritic Anterior Ischemic Optic Neuropathy	0 (0%)	1 (1.1%)
Otitis media	1 (1.3%)	0 (0%)
Pain	2 (2.5%)	1 (1.1%)
Palpitations	1 (1.3%)	0 (0%)
Pelvic infection	0 (0%)	1 (1.1%)
Pharyngolaryngeal pain	0 (0%)	1 (1.1%)
Pneumonitis	0 (0%)	2 (2.3%)
Postoperative haemorrhage	1 (1.3%)	0 (0%)
Proctitis	1 (1.3%)	0 (0%)
Pruritus	1 (1.3%)	0 (0%)
Psychiatric disorders - Other	0 (0%)	1 (1.1%)
Pulmonary oedema	0 (0%)	1 (1.1%)
Pulmonary infection	1 (1.3%)	0 (0%)
Renal and urinary disorders - Other	1 (1.3%)	0 (0%)
Respiratory, thoracic and mediastinal disorders - Other	1 (1.3%)	0 (0%)
Sepsis	0 (0%)	1 (1.1%)
Shingles	1 (1.3%)	1 (1.1%)
Shortness of breath	1 (1.3%)	0 (0%)
Sinus bradycardia	1 (1.3%)	0 (0%)
Sinusitis	0 (0%)	1 (1.1%)

	CCP group	SoC group
**Adverse events**		
**CTCAE terminology**		
Skin and subcutaneous tissue disorders - Other	3 (3.8%)	0 (0%)
Skin infection	0 (0%)	2 (2.3%)
Skin ulceration	0 (0%)	1 (1.1%)
Sore throat	2 (2.5%)	0 (0%)
Stroke	0 (0%)	1 (1.1%)
Sudden death NOS	0 (0%)	2 (2.3%)
Surgical and medical procedures - Other	0 (0%)	2 (2.3%)
Syncope	0 (0%)	1 (1.1%)
Testicular pain	0 (0%)	1 (1.1%)
Urinary fistula	0 (0%)	1 (1.1%)
Urinary tract infection	7 (8.9%)	3 (3.4%)
Ventricular arrhythmia	1 (1.3%)	0 (0%)
Voice alteration	0 (0%)	1 (1.1%)
Weight loss	1 (1.3%)	0 (0%)
Unknown	1	1

Table 7 (cont): Number and type of Adverse Events and Serious Adverse Events (according to CTCAE Terminology).

	CCP group	SoC group
**Total number of SAE**	**14**	**33**
**SAE (CTCAE Terminology)**		
Acute kidney injury	0 (0%)	2 (6.1%)
Atrial fibrillation	1 (7.1%)	0 (0%)
Back pain	0 (0%)	1 (3.0%)
Biliary tract infection	0 (0%)	2 (6.1%)
Bronchial infection	0 (0%)	1 (3.0%)
COVID-19 infection	0 (0%)	1 (3.0%)
Creatinine increased	1 (7.1%)	0 (0%)
Fever	0 (0%)	1 (3.0%)
Flu like symptoms	0 (0%)	1 (3.0%)
Gastrointestinal disorders - Other	0 (0%)	1 (3.0%)
Glaucoma	0 (0%)	1 (3.0%)
Hematoma	0 (0%)	2 (6.1%)
Haematuria	0 (0%)	1 (3.0%)
Infections and infestations - Other	1 (7.1%)	0 (0%)
Injury, poisoning and procedural complications - Other	0 (0%)	1 (3.0%)
Lung infection	3 (21%)	0 (0%)
Neoplasms benign, malignant and unspecified (incl. cysts and polyps) - Other	0 (0%)	4 (12%)
Non-Arteritic Anterior Ischemic Optic Neuropathy	0 (0%)	1 (3.0%)
Pharyngolaryngeal pain	0 (0%)	1 (3.0%)
Pneumonitis	0 (0%)	2 (6.1%)
Postoperative haemorrhage	1 (7.1%)	0 (0%)
Renal and urinary disorders - Other	1 (7.1%)	0 (0%)
Sepsis	0 (0%)	1 (3.0%)
Skin and subcutaneous tissue disorders - Other	1 (7.1%)	0 (0%)
Skin infection	0 (0%)	1 (3.0%)
Stroke	0 (0%)	1 (3.0%)
Sudden death NOS	0 (0%)	2 (6.1%)
Surgical and medical procedures - Other	0 (0%)	2 (6.1%)
Syncope	0 (0%)	1 (3.0%)
Urinary fistula	0 (0%)	1 (3.0%)
Urinary tract infection	5 (36%)	1 (3.0%)

Table S8. Change in serum anti-SARS-CoV-2 antibody concentration and neutralisation capacity of patients through day 28

	Geometric mean of change versus baseline on …					
	FU1 (day 3)		FU2 (day 14)		FU3 (day 28)	
	SoC	CCP	SoC	CCP	SoC	CCP
**Anti-SARS-CoV-2 QuantiVac ELISA (BAU/ml)**	261	1,829	953	1,145	1,732	1,158
**Elecsys anti-SARS-CoV-2 S (IU/ml)**	299	8,694	3,516	7,289	5,230	10,133
**GenScript Surrogate Neutralisation Test against wild type**	6.1	13.2	6.5	11.2	8.2	10.3
**GenScript Surrogate Neutralisation Test against Omicron**	3.7	53.5	11.1	33.8	16.2	32.6
**NT50 against BA.2**	433	2,029	882	1,123	1,519	1,656
**NT50 against BA.5**	267	1,042	494	586	1.111	799
**NT50 against XBB.1.5**	5	71	84	71	177	100
**NT50 against BQ.1.1**	4	125	53	122	110	138

This table provides supplementary information to **Figure 3 a** to **h** of the main manuscript. For analysis of the course of the anti-SARS-CoV-2 antibodies in the patients, the difference between the antibody concentrations at the follow-up examinations (follow-up 1 (FU1), day 3; follow-up 2 (FU2), day 14; and follow-up 3 (FU3), day 28) and the concentration before the start of therapy (baseline) was calculated for each individual patient and each follow-up time point (Δ from baseline values).

References

1. Desmarets M, Hoffmann S, Vauchy C et al. Early, very high-titre convalescent plasma therapy in clinically vulnerable individuals with mild COVID-19 (COVIC-19): protocol for a randomised, open-label trial. BMJ Open 2023;13:e071277.

2. Libster R, Perez MG, Wappner D et al. Early High-Titer Plasma Therapy to Prevent Severe Covid-19 in Older Adults. N Engl J Med 2021;384:610-8.

3. Klok FA, Boon GJAM, Barco S et al. The Post-COVID-19 Functional Status scale: a tool to measure functional status over time after COVID-19. Eur Respir J 2020;56.

4. O'Connor RJ, Preston N, Parkin A et al. The COVID-19 Yorkshire Rehabilitation Scale (C19-YRS): Application and psychometric analysis in a post-COVID-19 syndrome cohort. J Med Virol 2022;94:1027-34.

5. Gross R, Zanoni M, Seidel A et al. Heterologous ChAdOx1 nCoV-19 and BNT162b2 prime-boost vaccination elicits potent neutralizing antibody responses and T cell reactivity against prevalent SARS-CoV-2 variants. EBioMedicine 2021;75:103761.

6. Hoffmann M, Arora P, Gross R et al. SARS-CoV-2 variants B.1.351 and P.1 escape from neutralizing antibodies. Cell 2021;184:2384-93.

7. Hoffmann M, Kruger N, Schulz S et al. The Omicron variant is highly resistant against antibody-mediated neutralization: Implications for control of the COVID-19 pandemic. Cell 2022;185:447-56.

8. Arora P, Zhang L, Rocha C et al. Comparable neutralisation evasion of SARS-CoV-2 omicron subvariants BA.1, BA.2, and BA.3. Lancet Infect Dis 2022;22:766-7.

9. Arora P, Kempf A, Nehlmeier I et al. Augmented neutralisation resistance of emerging omicron subvariants BA.2.12.1, BA.4, and BA.5. Lancet Infect Dis 2022;22:1117-8.

10. Arora P, Kempf A, Nehlmeier I et al. Omicron sublineage BQ.1.1 resistance to monoclonal antibodies. Lancet Infect Dis 2023;23:22-3.

11. Hoffmann M, Arora P, Nehlmeier I et al. Profound neutralization evasion and augmented host cell entry are hallmarks of the fast-spreading SARS-CoV-2 lineage XBB.1.5. Cell Mol Immunol 2023;20:419-22.

12. Zhang L, Kempf A, Nehlmeier I et al. SARS-CoV-2 BA.2.86 enters lung cells and evades neutralizing antibodies with high efficiency. Cell 2024;187:596-608.

13. Berger RM, Zimmer G. A vesicular stomatitis virus replicon-based bioassay for the rapid and sensitive determination of multi-species type I interferon. PLoS ONE 2011;6:e25858.

14. Tyson JR, James P, Stoddart D et al. Improvements to the ARTIC multiplex PCR method for SARS-CoV-2 genome sequencing using nanopore. bioRxiv 2020.

15. Freed NE, Vlkova M, Faisal MB, Silander OK. Rapid and inexpensive whole-genome sequencing of SARS-CoV-2 using 1200 bp tiled amplicons and Oxford Nanopore Rapid Barcoding. Biol Methods Protoc 2020;5:bpaa014.

16. Aksamentov I, Roemer C, Hodcroft EB, Neher RA. Nextclade: clade assignment, mutation calling and quality control for viral genomes. Journal of Open Source Software 2021;6:3773.

17. O'Toole A, Scher E, Underwood A et al. Assignment of epidemiological lineages in an emerging pandemic using the pangolin tool. Virus Evol 2021;7:veab064.

18. Mercatelli D, Triboli L, Fornasari E, Ray F, Giorgi FM. Coronapp: A web application to annotate and monitor SARS-CoV-2 mutations. J Med Virol 2021;93:3238-45.

19. Newcombe RG. Interval estimation for the difference between independent proportions: comparison of eleven methods. Stat Med 1998;17:873-90.
