# Supplementary material for: Early, very high-titre convalescent plasma therapy in clinically vulnerable individuals with mild COVID-19: an international, randomised, open-label trial
Source: eBioMedicine. 2025 Feb 27;113:105613. doi: 10.1016/j.ebiom.2025.105613 (PMC11919330; doi:10.1016/j.ebiom.2025.105613)
Supplement: Protocol [file mmc2.pdf]

EudraCT No : 2021-006621-22
Protocol Number : NCT05271929

A Randomised Open-Label Trial of Early, Very High-Titre Convalescent Plasma Therapy in Clinically Vulnerable Individuals with Mild COVID-19
**COVIC-19 study**
**Version 2.4 23/02/2023**
**Sponsor's Protocol Code: COVIC-19**

Trial Steering Committee (in alphabetical order):

Thomas Appl

Daniel Bradshaw

Maxime Desmarets

Lise Estcourt

Sixten Körper

Bart Rijnders

David Roberts

Erhard Seifried

Hubert Schrezenmeier

Pierre Tiberghien

Eric Toussirot

A NOTE ABOUT THE COVIC-19 INTERNATIONAL TRIAL

COVIC-19 is an international study intended to recruit patients in European countries. Herein is presented the core protocol common to all countries involved. Country-specific annexes will be added for each participating country. The data will be collected and analysed centrally to ensure the power of the trial.

**France**	**Principal investigator in France**	**Pr. Eric Toussirot** (Centre Investigation Clinique INSERM CIC-1431),<br>Address: CHU Besançon, 25030 Besançon Cedex, France<br>Email: etoussirot@chu-besancon.fr<br><br>**Pr Antoine Durrbach (AP-HP Henri Mondor)**<br>Address: Nephrology and Transplantation Department, 94000 Creteil, France<br>Email: antoine.durrbach@aphp.fr
	**Scientific leader**	**Pr Pierre Tiberghien (EFS),**<br>Address: 20 Avenue du Stade de France - 93210 Saint-Denis
	**Study coordinator**	**Charline Vauchy** (Centre Investigation Clinique INSERM CIC-1431)<br>Address: CHU Besançon, 25030 Besançon Cedex, France
	**Sponsor in France**	**CHU de Besançon**<br>2, place Saint-Jacques<br>25030 Besançon cedex
		**Sponsor representative :**<br>**Chantal Carroger, Directrice générale**

**Germany**	**Principal investigator in Germany**	**Univ.Prof. Hubert Schrezenmeier, Univ.Prof.Dr.Dr.h.c. Erhard Seifried**<br>Address: Helmholtzstrasse 10, 89081 Ulm, Germany<br>Email: h.schrezenmeier@blutspende.de
	**Sponsor in Germany**	**German Red Cross Blood Transfusion Service Baden-Württemberg-Hessen**<br>Friedrich-Ebert-Straße 107<br>68167 Mannheim
		**Sponsor representative:**<br>**Univ-Prof.Dr.h.c. Erhard Seifried**

**Netherlands**	**Principal investigator in the Netherlands**	**Bart Rijnders**<br>Address:<br>Email: b.rijnders@erasmusmc.nl
	**Sponsor in the Netherlands**	**Erasmus Medical Center**
		**Sponsor representative:**

**United Kingdom**	**Principal investigator in the United Kingdom**	***Lise Estcourt, Daniel Bradshaw and Pr. David Roberts***<br>NHS Blood and Transplant, Oxford<br>Email: Lise.Estcourt@nhsbt.nhs.uk<br>Email: David.roberts@nhsbt.nhs.uk
	**Sponsor in the United Kingdom**	**National Health Services Blood and Transfusion (NHSBT)**

**Study methodologist**	**Maxime Desmarets**<br>Centre Investigation Clinique INSERM CIC-1431<br>CHU Besançon, France

Confirmation of Trial Protocol / Signature page

Study Title: A Randomised Open-Label Trial of Early, Very High-Titre Convalescent Plasma Therapy in Clinically Vulnerable Individuals with Mild COVID-19

This is a Phase III randomized clinical study to assess the feasibility, safety and efficacy of convalescent plasma for treatment, collected from donors who have recovered from COVID-19 and who have a very high titre of anti-SARS-CoV-2 antibodies, reduce the risk of hospitalisation (for COVID-19) or death in patients with early symptoms of acute COVID-19 who are vulnerable to this disease compared to standard of care.

The signatories declare that they agree to conduct their responsibilities within this trial in accordance with local law, the declaration of Helsinki, ICH-GCP and the trial protocol as presented.

Approved by the following:

Protocol Agreement / Signature page for Investigator

Title: A Randomised Open-Label Trial of Early, Very High-Titre Convalescent Plasma Therapy in Clinically Vulnerable Individuals with Mild COVID-19

This is a Phase III randomized clinical study to assess the feasibility, safety and efficacy of convalescent plasma for treatment, collected from donors who have recovered from COVID-19 and who have a very high titre of anti-SARS-CoV-2 antibodies, reduce the risk of hospitalisation (for COVID-19) or death in patients with early symptoms of acute COVID-19 who are vulnerable to this disease compared to standard of care.

I declare that I have read and understood the protocol and agree to conduct the trial accordingly. I will ensure that all persons working on the trial under my supervision are adequately informed about the protocol, the investigational medicinal product and their duties.

Name of Hospital : ______________________________

Name of Investigator: ____________________________

Signature Investigator: ___________________________

Signature Co-Investigator: ________________________________

Date: ______

List of Abbreviations:

Blood group system	ABO
Body Mass Index	BMI
Acute respiratory distress syndrome	ARDS
Adverse event	AE
Adverse reaction	AR
Alanine Aminotransferase	ALT
Area under curve	AUC
Aspartate transaminase	AST
Bronchoalveolar lavage	BAL
C-reactive protein	CRP
Chimeric antigen receptor	CAR
Chronic obstructive pulmonary disease	COPD
Confidence interval	CI
Coronavirus disease 2019	COVID-19
Case report form	CRF
Clinical research organization	CRO
Common Terminology Criteria of Adverse Events	CTCAE
Clinical Research Associate	CRA
Curriculum vitae	CV
Data Safety Monitoring Board	DMSB
Day	D
Degree Celsius	°C
electronic case report form	eCRF
Extracorporeal membrane oxygenation	ECMO
Enzyme-linked Immunosorbent Assay	ELISA
Ethics committee	EC
Ethylenediaminetetraacetic acid	EDTA
U.S. Food and Drug Administration	FDA
Fraction of inspired oxygen	FiO2
Fresh frozen plasma	FFP
Good Clinical Practice	GCP
Graft-versus-Host-Disease	GVHD
Hepatitis A	HAV
Hepatitis B	HBV
Hepatitis C	HCV
Hepatitis E	HEV
Human immune deficiency virus	HIV
Human leukocyte antigen	Ig
Immunoglobulin	HLA
Intensive care unit	ICU
International Conference on Harmonisation - Good Clinical Practice	ICH-GCP
Investigational medicinal product	IMP
Investigational medicinal product dossier	IMPD
Investigator site file	ISF
Intent to treat	ITT
Intensive Therapy Unit	ITU

Agence nationale de sécurité du médicament (French Agency for the Safety of Health Products)	ANSM
Lactate dehydrogenase	LDH
Medicines and Healthcare products Regulatory Agency (medical licensing and regulatory authority for medicinal products in the UK)	MHRA
Middle East respiratory syndrome	MERS
Modified intention to treat population	mITT
Multi-organ dysfunction syndrome	MODS
Nasopharyngeal swabs	NP
National Health Service Blood and Transplant	NHSBT
New York Heart Association	NYHA
Non-invasive ventilation	NIV
Nonsteroidal anti-inflammatory drugs	NSAIDS
Oxygen saturation	SpO2
Paul-Ehrlich Institut (German Federal Institute for Vaccines and Biomedical Products)	PEI
Qualified person	QP
Quality of life	QoL
Partial pressure of oxygen	pO2
Polymerase chain reaction	PCR
Randomized controlled trial	RCT
Reverse transcription polymerase chain reaction	RT-PCR
Ribonucleic acid	RNA
Riker Sedation-Agitation Scale	SAS
Severe acute respiratory syndrome coronavirus	SARS-CoV
Severe acute respiratory syndrome coronavirus 2	SARS-CoV-2
Serious adverse event	SAE
Standard operating procedure	SOP
Suspected unexpected serious adverse reaction	SUSAR
Transfusion associated circulatory overload	TACO
Transfusion related lung injury	TRALI
Unit	U
Usual Interstitial Pneumonia	UIP
World Health Organization	WHO

TABLE OF CONTENTS

A note about the COVIC-19 international trial 3
Table of contents 9
Version history 12
Study schedule 13
I. Study Presentation - Context 14
II. Background and rationale 15
II-1. WHAT IS THE PROBLEM BEING ADDRESSED? 15
II-2. WHY IS THIS RESEARCH IMPORTANT? 15
II-3. HOW DOES THE EXISTING LITERATURE SUPPORT THIS PROPOSAL? 16
II-3-1. COVID-19 in vulnerable patients 16
II-3-2. Convalescent plasma to treat infectious diseases 16
II-3-3. Covid-19 convalescent plasma to treat COVID-19 17
II-4. WHAT IS THE RESEARCH QUESTION? 18
II-5. Risk/Benefit assessment 19
II-5-1. Known potential Risks 19
II-5-2. Known potential benefits 20
II-6. Experimental medicinal product and justification for dosage and dosage regimen 20
II-7. Patient population 21
III. Study objectives and endpoints 22
III-1. Primary objective 22
III-2. Secondary objectives 22
III-3. Exploratory objectives 22
III-4. Study endpoints 22
IV. Study design 25
IV-1. Overall design 25
IV-2. Rationale for study design 25
IV-3. Study duration and dates 25
V. Study Population 26
V-1. Inclusion criteria 26
V-1-1. Elderly and high COVID-age population: 26
V-1-2. High-risk immunocompromised population 26
V-2. Exclusion criteria 27
V-2-1. Elderly and high COVID-age population: 27
V-2-2. High-risk immunocompromised population 27

VI. Study Assessments and procedures ______ 27
VI-1. Screening Assessment ______ 27
VI-2. Screen Failures ______ 28
VI-3. Baseline/ Follow-up ______ 28
VI-4. Data recorded ______ 31
VI-4-1. Baseline information ______ 31
VI-4-2. Follow up information ______ 31
VII. Study Product ______ 32
VII-1. Investigational therapeutic ______ 32
VII-2. Convalescent plasma collection ______ 32
VII-3. Storage, Handling, Distribution and Stability ______ 32
VII-4. Administration ______ 33
VII-5. Appearance ______ 33
VII-6. Justification for dose ______ 33
VII-7. Authorised medications ______ 33
VII-8. Discontinuation of study intervention ______ 33
VII-8-1. Procedures for replacing these people, if applicable ______ 34
VII-8-2. Procedures for monitoring these people ______ 34
VII-9. Participant discontinuation/withdrawal from the study ______ 34
VII-10. Study discontinuation ______ 34
VIII. Measures to minimise bias ______ 34
VIII-1. Randomisation ______ 34
VIII-2. Maintenance of group comparability ______ 34
IX. Safety reporting ______ 35
IX-1. Criteria for the evaluation of safety ______ 35
IX-1-1. Identified safety data ______ 35
IX-1-2. Regulatory definitions. ______ 35
IX-2. Methods and schedule for measuring, collecting and analysing these parameters (details in appendix) ______ 38
IX-2-1. Intensity of an AE ______ 38
IX-2-2. Causal relationship between AEs and the study procedures ______ 38
IX-3. Procedures for Adverse Events ______ 38
IX-4. Data Safety and Monitoring Board ______ 38
IX-5. Methods and duration of follow-up following the occurrence of an adverse event (details in appendix) ______ 38
X. Statistical methods ______ 38

X-1. Analysis populations ____ 38
X-1-1. Flow diagram ____ 38
X-1-2. Intention to treat population ____ 38
X-1-3. Safety analysis population ____ 39
X-2. Sample size determination ____ 39
X-3. Statistical analysis ____ 40
X-4. Degree of significance ____ 42
X-5. Stopping rules ____ 42
X-6. Handling of missing or incoherent ____ 42
X-7. Modifications to the statistical analysis plan ____ 42
XI. Data handling and record keeping ____ 43
XI-1. Data collection and entry ____ 43
XI-2. Data transfer ____ 43
XI-4. Record keeping and archiving ____ 43
XII. Regulatory, ethical considerations ____ 43
XII-1. Information and consent of the subject ____ 43
XIII. Quality assurance and quality control ____ 44
XIV. Publication ____ 44
XV. References ____ 44

Illustrations

Table 1. Procedures performed on convalescent plasma as part of the protocol.......... 21
Table 2. 10-point WHO Progression scale.......... 24
Table 3: Table of assessments.......... 29

VERSION HISTORY

Version No.	Date	Main changes
V1.0	16/02/2022	First version
V1.1	18/03/2022	Added annex describing current standard of care
V2.0	12/05/2022	Removed O_2 requirement from primary endpoint and added adjudication of primary endpoint<br>Added neutralisation assay against current and future variants for donor selection.<br>Added viral cultivability objectives
V2.1	19/05/2022	Update Trial Steering Committee
V2.2	20/07/2022	Modification of the test for sample size determination (Z-test)<br>Clarification of the statistical paragraphs of the protocol<br>Use of a two-tailed test (alpha 0.05) for the primary endpoint
V2.3	09/08/2022	Reminder of the unvaccinated status of patients in cohort 1<br>Removal of hemoglobinopathies from the inclusion criteria of cohort 1<br>Reminder of the use of standard of care in the PCC arm<br>Reminder of the use of anti-SARS-CoV-2 therapy in the SoC<br>Indication of the WHO scale levels corresponding to severe COVID-19, requiring or not oxygen supplementation<br>Consistency of the exploratory endpoints with the biological samples of the study<br>Reminder: any patient eligible for both cohorts is included in cohort 2.<br>Vigilance: removal of thrombotic events from the list of expected SARs<br>Vigilance: addition of the definition of a serious incident
V2.4	23/02/2023	Inclusion criteria modification: added “or positive antigenic test”.

STUDY SCHEDULE

- Regulatory procedures: 4 months

- Inclusion:
  Step 1: 12 months
  Step 2: 12 to 24 months

- Follow-up: 6 months

- Total study duration: 18 to 42 months

- Data analysis:
  Step 1: 1 month for main objective / 2 months for secondary objectives
  Step 2: 2 months

I. STUDY PRESENTATION - CONTEXT

The COVIC-19 study is a prospective randomized clinical study evaluating the benefit of COVID-19 convalescent plasma (CCP) with a very high antibody titre administered early, on an outpatient basis, to vulnerable patients to prevent the progression of a COVID-19 disease to a severe form requiring hospitalization. The vulnerable patients concerned by this study are patients with recently diagnosed COVID-19 disease who do not need hospitalisation and who are: at least 70 years old; younger patients with risk factors for severe COVID-19; and patients with primary or secondary immunodeficiency. Patients randomized to the treatment arm will receive 2 units of CCP with high titre anti-SARS-CoV-2 antibody on an outpatient basis within 7 days of the onset of symptoms of COVID-19. Patients in the control group will not receive a specific intervention.

This study is based on growing evidence for the effectiveness of passive immunotherapy, by transfusion of CCP or administration of monoclonal antibodies, as long as treatment is carried out early after the diagnosis of the disease. In a recent randomized clinical trial in Argentina by Libster et al[1], the administration of CCP less than 3 days after the onset of symptoms of COVID-19 in vulnerable patients halved the risk of developing severe COVID-19 disease compared to a control group (16% after CCP transfusion vs. 31% without transfusion). Similar findings were recently reported in a clinical trial coordinated by the John Hopkins Hospital (USA).[2] A third randomized trial found similar results as well for patients included less than 5 days after symptoms initiation.[3] A 4th study did not confirm this efficacy of CCP, with however inclusion criteria that differed significantly (patients recruited in emergency rooms).[4]

Importantly, a significant antibody dose response was observed. Likewise, the early administration of monoclonal anti-SARS-CoV-2 antibodies in mono- or bitherapy has been associated with a decrease in the frequency of hospitalization for COVID-19 disease.

Lastly, French data from the Therapeutic Use Protocol (monitored access program authorized by the French regulatory agency ANSM) as well as from a recent American propensity score matching study pertaining to the efficacy of CCP in patients unable to mount an anti-SARS-CoV-2 humoral immune response also plead in favour of an early intervention in these same patients.[5,6]

The primary endpoint of the COVIC-19 study will be the proportion of patients requiring hospitalization at least one overnight for progressive COVID-19 symptoms, or who have died within 28 days after randomization.

The COVIC-19 study will include two distinct cohorts: firstly, unvaccinated elderly and younger patients with co-morbidities, and secondly, immunocompromised patients. This distinction is justified in particular by the differences between these two groups with regard to the characteristics of the COVID-19 disease, differences accentuated by the effects of the vaccination, significant in the 1st cohort, much less so in the 2nd cohort. The results in the two cohorts will be analyzed separately.

The COVIC-19 study will be carried out in 2 stages. A 1st stage has the objective of confirming or not the data of Libster et al[1] in each of the cohorts (50% reduction of a risk of a 30% aggravation rate) with a power of 90%, an alpha risk of 5% (and + 5% of additional patients). The number of patients required is 340 in each cohort, for a total of 680 patients. For each cohort, an unblinded interim analysis will be performed when 30% of the patients of the 1st stage reach the primary endpoint. The aim of this analysis will be to reestimate the sample size of the trial. If the conditional power for detecting the difference in primary outcome between the two arms in the final analysis is between 50% and 90% and if the feasibility is confirmed, the sample size will be increased in a 2nd stage in order to achieve 90% power to detect the effect observed at the interim analysis, with a maximum sample size of 1020 per cohort. Otherwise, the trial will continue up to the sample size planned in the 1st stage and then terminate.

This European study will be deployed in France, England, Netherlands and Germany, with significant support from the European SUPPORT-E consortium (http://www.support-e.eu) or national funding sources. Each country will have coordination and promotion. Like other early therapeutic approaches to COVID-19, the transfusion of CCP very early in the disease requires the establishment or amplification of outpatient intervention methods, here with plasma transfusion, relying on day-care hospitals, doctor´s offices, but also on home care or Elderly long-term care centres and possibly on health centres.

The advent of vaccinated convalescent donors will allow for the provision of CCP with a very high Ab titre and possibly endowed with a cross-reactive specificity, i.e. an ability to neutralize variants to which the donor has not been exposed.[7,8] This orientation towards vaccinated convalescent donors will constitute a singular advantage compared to plasmas used in previous or current clinical studies and compared to monoclonal antibodies. Overall, CCP adaptability and short turn around sets CCP apart from other passive immunotherapy approaches for COVID-19, namely monoclonal antibodies and hyperimmune immunoglobulins (Ig).

The number of apheresis procedures to be performed (in selected donors) for this study remains limited. For the 1st stage of the study, the number of patients to be transfused in each study country is ≈ 113, i.e. ≈ 226 units of CCP. Considering 1. that a donation of apheresis allows the production of 2 to 3 units of plasma, 2. that 50% of the donations would not have a sufficient titre despite the restriction to convalescent vaccinated donors, 3. the additional units needed to take into account ABO compatibility needs and other logistical considerations, the total number of donations required for the study is ≈ 1356-2040 (all countries).

II. BACKGROUND AND RATIONALE

II-1. WHAT IS THE PROBLEM BEING ADDRESSED?

The Severe Acute Respiratory Syndrome Coronavirus 2 (SARS-CoV-2) pandemic has caused more than 5 million deaths worldwide. Mortality is 40% or more for patients from clinically vulnerable groups admitted to hospital.[9] Therefore, treatment is urgently needed soon after symptom onset, to prevent progression to severe disease so avoiding hospitalisation. This is especially important in clinically vulnerable patients who are at high risk of death or prolonged hospitalisation from COVID-19. Furthermore, patients with immunosuppression or immunodeficiency have been disproportionately affected by the COVID-19 pandemic, and often present with persistent SARS-CoV-2 infection and may shed viable SARS-CoV-2 for months.[10] Vulnerable patients may belong to subgroups less likely to respond well to vaccination. We will evaluate the efficacy of CCP collected from donors with high-titre neutralising SARS-CoV-2 antibodies in reducing the risk of hospitalisation in people with early COVID-19 compared to standard care. We will do this through a randomised, open-label two-arm trial in clinically vulnerable patients that will provide robust comparative efficacy data in groups most likely to benefit from early treatment. We will assess virological parameters by sequentially measuring SARS-CoV-2 RNA and virus viability in oropharyngeal samples, antibody levels and viral sequence variation.

II-2. WHY IS THIS RESEARCH IMPORTANT?

People in clinically vulnerable groups will continue to be exposed to SARS-CoV-2 during future waves, at hospital visits, in the community and at home and so continue to be at risk of severe disease and death. Early CCP may reduce morbidity and mortality from COVID-19 pneumonia and avoid complications such as secondary bacterial infections and need for hospitalisation. In addition, CCP may be of particular benefit in people in whom a SARS-CoV-2 vaccine may not elicit effective immune responses, such as older people, those with congenital immunodeficiency or the large number of

patients who are immunocompromised due to underlying disease and/or immunosuppressive therapy.

Faster resolution of COVID-19 may avoid delays to life-saving treatments, such as stem cell transplantation. Antibody therapies in early COVID-19 have the potential to improve quality of life and reduce long-term COVID-19 symptoms. Wider benefits may include reduced burden on limited healthcare resources by reducing hospital admissions for COVID-19 and increasing resources for urgent, non-COVID-19 work in hospitals.

Faster viral clearance in patients treated early will reduce the period of infectivity, conferring particular benefit for immunocompromised people in whom viral shedding can be prolonged, people on haemodialysis, who require frequent visits to healthcare facilities, and those in residential settings with high risk for outbreaks e.g. care homes. Demonstration of CCP effectiveness in outpatient settings may influence future policy surrounding lockdown measures and inform health resource planning. Importantly, early in a pandemic, or in case of the emergence of an immuno-resistant variant, CCP may be the only passive immunotherapy approach readily available. Also, when available, access to monoclonal antibodies or hyperIg may be limited by manufacturing or costs issues. It already has been observed that SARS-CoV-2 spike mutations in newly emerging variants confer resistance against monoclonal antibodies limiting their potential benefit.

A clinical trial of CCP remains important – even in an era of availability of effective vaccines. It is unclear how many individuals will finally choose to become vaccinated .[11] Efficacy of vaccination might decrease over time.[12] New SARS-CoV-2 variants might escape the vaccine-induced immune response.[13] Patients with compromised immune reaction due to underlying disease and or immunosuppressive treatment (i.e. cohort 2 of this trial) might fail to mount an adequate vaccination response.[14–17]

II-3. HOW DOES THE EXISTING LITERATURE SUPPORT THIS PROPOSAL?

II-3-1. COVID-19 in vulnerable patients

Large cohort studies identified advancing age to be the greatest risk for hospitalisation and death in people with COVID-19.[18] Other risk factors include comorbidities, immunosuppression, male sex, and deprivation.[19–21] Health authorities have listed clinically extremely vulnerable groups who are advised to shield during periods of high COVID-19 incidence.[22] Hopefully, a large fraction of these groups are now vaccinated and protected. Nevertheless, for those unvaccinated, or in the event of an immune-resistant variant, older people, those with multiple comorbidities or those recommended to shield, represent groups most likely to benefit from treatment of early COVID-19. Immunosuppressed patients are the least likely to benefit from a SARS-CoV-2 vaccine and are at risk of prolonged (>2 months) shedding of SARS-CoV-2 RNA and so, recurrence of symptoms while posing a risk of sustained onward transmission or selection of variants and so may gain additional benefits from early therapeutic intervention.[9,23]

II-3-2. Convalescent plasma to treat infectious diseases

Convalescent plasma is a passive polyclonal antibody therapy that has been used to prevent or treat infectious diseases for more than a century.[24] The anti-pathogen antibodies from convalescent plasma can mitigate infection by two main mechanisms: antibody effector activity and pathogen neutralization. The polyclonal nature of CCP, in which a spectrum of differentiated antibodies target multiple epitopes of the pathogen, may help to reduce the risk of emergence of viral variants.

Convalescent plasma treatment has been used to improve the survival rate of patients with severe acute respiratory syndromes of viral aetiology.[25] Indeed, a number of studies, unfortunately all inadequately controlled for bias, have reported positive outcomes, including decreased mortality in the so-called Spanish influenza A (H1N1) infections in 1915–1917, the more recent influenza A (H1N1) infections in 2009/2010, and SARS-CoV infections in 2003. A systematic review and exploratory meta-analysis performed in 2014 revealed evidence for a consistent reduction in mortality with plasma therapy.[25] While inconclusive, such findings favored an administration of convalescent plasma as close

as possible to the onset of the infectious course, at a time where pathology may be driven mainly by viral replication.

II-3-3. Covid-19 convalescent plasma to treat COVID-19

COVID-19 convalescent plasma (CCP) has been, and still is assessed in a large number of clinical trials, mainly in hospitalized patients. By and large, most trials performed in hospitalized patients have so far been unable to demonstrate a beneficial effect of convalescent plasma,[26,27] while often offering evidence that early to very early transfusion of high titre CCP may be advantageous.[28–32] Accordingly, and as mentioned earlier, a recent randomized study exhibited a favorable effect of very early administration of high-titre CCP in vulnerable patients.[1] Libster et al. randomized vulnerable older adult patients within 72 h after onset of mild COVID-19 symptoms to receive CCP with high IgG titres against SARS-CoV-2 or placebo. The primary end-point was the occurrence of severe respiratory disease. A total of 160 patients underwent randomization. In the intention-to-treat population, severe respiratory disease developed in 13 of 80 patients (16 %) who received CCP and 25 of 80 patients (31 %) who received placebo (relative risk, 0.52; 95 % confidence interval [CI], 0.29 to 0.94; P =0.03), with a relative risk reduction of 48 %. A modified intention-to-treat analysis that excluded 6 patients who had a primary end-point event before infusion of CCP or placebo showed a greater effect size (relative risk, 0.40; 95 % CI, 0.20 to 0.81). Notably, an Ab dose effect was observed with a 73 % reduction in disease worsening in recipients of CCP with a titre at or above the median concentration versus 31,4% in recipients of CCP with a titre below the median concentration. No adverse events were reported.

Another trial of early CCP for high-risk outpatients with COViD-19 (SIREN-C3PO) failed to demonstrate a significant reduction of disease progression to severe COVID-19 .[4] However, this trial differed in many aspects from the INFANT-COVID-19 trial ("Libster trial"): median age of patients was 54 years (vs. mean age 77 years in the INFANT-COVID-19 trial) and patients in the SIREN-C3PO presented to the emergency department within 7 days after symptom onset, i.e. the interval from symptom to treatment was longer than in the INFANT-COVID-19 trial and the patient population might represent a more unwell subgroup since they were seeking emergency department care. The primary outcome in the SIREN-C3PO trial is a composite of hospital admission for any reason, seeking emergency or urgent care or death. Thus, in contrast to the INFANT-COVID-19 trial the endpoint of the SIREN-C3PO is not limited to progress to severe respiratory disease due to COVID-19 based on objective criteria. The design of this present trial COVIC-19 is close to the INFANT-COVID-19 trial.

In a recently published study, Sullivan et al. report on a large trial involving 1181 outpatients who received either convalescent plasma or control plasma.[33] In this double-blind, randomized, controlled trial, SARS-CoV-2-positive participants were assigned to receive a transfusion of CCP or control plasma within 9 days after the onset of symptoms. The primary outcome was COVID-19-related hospitalisation within 28 days after transfusion; this outcome occurred in 17 of 592 participants (2.9%) who received convalescent plasma and in 37 of 589 participants (6.3%) who received control plasma (absolute risk reduction, 3.4 percentage points; 95% confidence interval, 1.0 to 5.8; P=0.005). Although the time to include participants after the onset of symptoms was longer than in the INFANT-COVID-19 and SIREN-C3PO trials, 44% of patients received a transfusion within 5 days. A total of 89 adverse events of grade 3 or 4 were reported (34 in the convalescent-plasma group and 55 in the control-plasma group), including 44 cases of pneumonia. A total of 16 grade 3 or 4 adverse events (7 in the convalescent-plasma group and 9 in the control-plasma group) occurred in participants who were not hospitalized.

Importantly, CCP may also benefit immunosuppressed patients unable to mount a humoral immune response to SARS-CoV-2.[5,6,34] Indeed, in patients with protracted COVID-19 unable to mount an antibody response to SARS-CoV-2, CCP has been associated with striking clinical improvement, albeit in an uncontrolled setting.[5] In one large matched-control study, the 30-day mortality was evaluated in hospitalized adults with hematologic malignancy and COVID-19, comparing 143 patients transfused with CCP and 823 matched controls who received standard care treatment.[6] Among these patients

with haematological malignancy, CCP treatment was associated with a lower risk of death after adjustment for potential confounding factors (hazard ratio, 0.60; 95% confidence interval [CI], 0.37–0.97) or after propensity-score matching (hazard ratio, 0.52; 95% CI, 0.29–0.92). Several case reports and case series provide further evidence suggesting a mortality benefit and rapid clinical improvement in patients with several forms of immunosuppression following CCP transfusion.[34] In the REMAP-CAP trial,[30] a small sub-group of patients with immunodeficiency or immune suppressive therapy were analysed as part of a pre-specified sub-group. There was an 89.9% probability that CCP was superior to standard care, whereas there was no evidence of benefit in those patients with critical disease who had an intact immune system. The RECOVERY trial also identified no evidence of benefit in hospitalised patients overall, but sub-group analyses showed that those patients treated within 7 days of symptom onset may benefit. The Cochrane living systematic review has highlighted the uncertainty of the evidence for early treatment and the minimal evidence for patients with underlying immunodeficiencies.[35]

CCP RCTs have also shown that a large number of hospitalised patients had antibodies to the virus before any treatment, suggesting that antibody therapy may be ineffective in patients who have already mounted an immune response. Of note, several studies have established that in contrast to antibodies in patients with mild disease or at time of convalescence, early antibody responses in critically ill patients may exhibit pro-inflammatory properties and reduced anti-viral efficacy.[36] Therefore, transfusing CCP to a patient in the process of mounting an immune response may provide direct antiviral activity while possibly abating deleterious inflammation. Furthermore, early administration of a combination of anti-SARS-CoV-2 monoclonal antibodies has recently proven to be associated with a reduction in progression to severe COVID-19 disease as well,[37,38] thus confirming efficacy of early passive immunotherapy. As highlighted in the Libster study mentioned earlier,[1] transfusing CCP with a high titre of neutralizing Ab may be quite critical. A similar Ab dose effect has been observed elsewhere as well.[39,40] Recent data demonstrate that the combination of convalescence from natural infection combined with a SARS-CoV-2 vaccination causes both an enhancement of all aspects of the humoral immune response and a broad immune reaction against new variants.[7,8,41] The underlying mechanisms involve ongoing antibody somatic mutation, memory B cell turnover, and development of antibodies that are resistant to SARS-CoV-2 RBD mutations, including those found in variants of concern.[41] Thus, convalescent vaccinated donors may provide plasma with very high anti-SARS-CoV-2 Ab titre with an addition potential cross-reactive specificity, i.e. an ability to neutralize variants to which the donor has not been exposed.

II-4. WHAT IS THE RESEARCH QUESTION?

In people with mild COVID-19 symptoms aged 70 years or over, or who are under 70 years with multiple comorbidities or immunosuppression, does administration of convalescent plasma containing high titres of neutralising SARS-CoV-2 antibodies in addition to standard care compared to ‘standard care’ alone reduce the risk of hospitalisation (defined as at least one overnight inpatient hospital stay with progressive COVID-19 symptoms) or death within 28 days.

Further objectives are to provide, in early COVID-19:

1) Evidence of whether CCP benefits clinically vulnerable patients;

2) Frameworks for administration of CCP to treat outpatients;

3) Monitoring of virological response to CCP including SARS-CoV-2 RNA levels, antibody titres, viral sequence data and ability to isolate virus;

4) Evidence of whether early intervention in COVID-19 reduces the likelihood of long-term complications.

II-5. Risk/Benefit assessment

II-5-1. Known potential Risks

Known potential risks are mostly those associated with the transfusion of plasma. Adverse effects associated with plasma transfusion CCP include allergy, febrile reactions, transfusion-related circulatory overload (TACO) and Transfusion Related Acute Lung Injury (TRALI). Of note, the frequency of TRALI has significantly lowered since the introduction of anti-HLA Ab screening in child-bearing female donors.

The most frequent adverse event associated with plasma transfusion reported by the French hemovigilance[42] (2019, 266 135 plasma units transfused) is by far allergic reactions with a reported frequency of 113,7/100 000 transfused plasma (grade 2 and 3), followed by TACO and febrile non haemolytic reactions with frequencies of 4.1/100 000 for both. Overall frequency of severe (grade 3) adverse events was 11.4 /100 000, mostly allergies (n=28, 93%) and less frequently TACO (n=1, 3.5%) and TRALI (n=1, 3.5%). No grade 4 (death) has been reported with plasma transfusion since now several years in France.

Data show that CCP is well tolerated. In a Cochrane review, amongst 20 000 people with COVID-19 receiving CCP in non-controlled non-randomised studies of interventions and for whom adverse events were recorded, there were 63 deaths of which 12 were possibly and 1 probably related to transfusion (13/20622, 0.06%). There were 146 serious adverse events (SAE) within 4 hours and 1136 SAEs within 7 days post-transfusion, predominantly allergic, respiratory, thrombotic or cardiac events (1282/20 000, 6.4%).[35] Four RCTs observed severe or serious transfusion-related adverse events in 0 to 1.3% of participants receiving convalescent plasma, including severe transfusion-associated dyspnoea, and probably-transfusion-related deaths. The comparator trials are inconclusive regarding the occurrence of grade 3/4 AEs, but the data support a reduction in the risk of SAEs after CCP treatment (Convalescent plasma versus standard plasma, low-certainty evidence).

In the Recovery trial[27] (11558 hospitalized patients with over 70 % requiring respiratory support), severe allergic reactions were reported in 16 (<1%) of 5795 patients in the COVID-19 convalescent plasma group and two (<1%) of 5763 patients in the usual care group within the first 72 h after randomisation. The frequency of sudden worsening in respiratory status, temperature higher than 39°C or a 2°C or higher increase in temperature above baseline, sudden hypotension, clinical haemolysis, and thrombotic events were broadly similar in the two groups.[27] No significant differences in the frequency of major cardiac arrhythmia were observed. Thirteen patients receiving convalescent plasma had reports submitted to the Serious Hazards of Transfusion haemovigilance scheme: nine patients with pulmonary reactions (none considered to be transfusion-related acute lung injury, including three deaths possibly related to transfusion), and four patients with serious febrile, allergic, or hypotensive reactions (all of whom recovered).

The reported studies pertaining to early CCP administration in mildly ill vulnerable COVID-19 patients (as for patients to be included in COVIC-19) reported no adverse events[1], or adverse events occurring with similar frequency in the CCP and saline control groups except for dyspnea, which occurred more often in the placebo group, and infusion-related reactions, which occurred more often in the convalescent-plasma group[4], or no significant inbalance in the frequency of severe adverse events between the CCP group and standard plasma control group.[2]

Passive immunotherapy as well as other treatment such as remdesivir may result in the selection of resistant variants, notably in immunosuppressed patients with prolonged viral carriage.[43,44] The polyclonal nature of CCP may result in a lesser risk than with anti-SARS-CoV-2 monoclonal antibodies. It has been demonstrated that some SARS-CoV-2 spike mutations in new variants confer resistance to monoclonal antibodies. Variants and associated resistance are regularly updated in the Coronavirus Antiviral and Resistance Database maintained by Stanford University.[45–54]

Lastly, transient lung-function worsening (transient increased O_2 requirement) early after CCP transfusion and without evidence of circulatory overload has been observed in several immunosuppressed patients hospitalized for severe COVID-19 in France (unpublished data). Importantly, such occurrences, always transient, do not prevent a subsequent favourable effect of CCP on the course of COVID-19. The mechanisms involved are currently unknown and could hypothetically result from immune complex formation in airway tissues of severely ill patients unable to clear SARS-CoV-2.[55] It is expected that patients at earlier stage of the disease, and notably in the absence of severe lung disease, will be at lesser risk of such occurrences. Such early transient respiratory worsening has not been reported in the Libster study mentioned earlier as well as other studies involving immunosuppressed patients.

II-5-2. Known potential benefits

As highlighted earlier, early transfusion of high titre CCP in vulnerable patients may significantly reduce the risk of disease worsening and need for hospitalization. Severe COVID-19 disease in vulnerable patients such as elderly patients, less elderly with comorbidities or patients with immunodeficiency remains associated with significant morbidity and mortality. Furthermore, immunosuppressed patients, least likely to benefit from a SARS-CoV-2 vaccine, are at risk of prolonged (>2 months) shedding and pose a risk of sustained onward transmission or selection of variants and so may gain additional benefits from early therapeutic intervention. Furthermore, reducing the need for hospitalization will also reduce the burden on hospital means at time of crisis. To the difference of anti-SARS-CoV-2 monoclonal antibodies, CCP may be readily available and adapted in the presence of an emerging immune-resistant SARS-CoV-2 variant or in case of a pandemic involving a novel pathogen.

The polyclonal antibody content of CCP could make this an ideal passive immunotherapy for treatment of patients infected by new variants. The very recent and rapidly progressing emergence of the Omicron variant with a high number of mutations in the Spike protein lead to viral escape and challenge existing monoclonal antibodies.[54] In-vitro studies show resistance of SARS-CoV-2 Omicron to casirivimab and imdevimab and also other monoclonals (including balavimab/etesivimab; cilgavimab/tixagevimab; regdanvimab) (see also data collection on SARS-CoV-2 mutations conferring resistance to monoclonal antibodies at https://opendata.ncats.nih.gov/variant/activity).[53,54] There is now rapidly growing evidence, that Omicron escape is incomplete in previously infected individuals with very high titer polyclonal antibodies,[54,56] in particular “super immune” donors with combination of infection/vaccination or vaccination /infection maintain neutralization capacity against Omicron.[57] In-vitro studies demonstrated that in particular previous infection, followed by vaccination or booster is likely to increase the neutralization level and likely confer protection from severe disease in Omicron infection.[56] Also breakthrough infections in previously vaccinated individuals substantially boost neutralizing antibody titers.[58] Thus, recent data very much support the approach in this trial: selection of very high-titre plasma donors amongst those strongly immunized by a combination of both infection and vaccination. Given the rapid worldwide spread of the previous variants (Alpha, Beta, Delta and now Omicron) it is likely that further variants will emerge which are resistant to existing monoclonal antibodies and thus warrants the study of the broad and adaptive immune response in “superimmunised” individuals after infection/vaccination or vaccination/ infection.

II-6. Experimental medicinal product and justification for dosage and dosage regimen

Medicinal product: COVID-19 convalescent plasma (CCP)

Dosage: Two units (200 to 350 mL per unit) of high titre CCP.

Convalescent plasma should be derived by apheresis from vaccinated donors, collected at least 3 weeks post first dose of vaccine, who have had a previous laboratory confirmed SARS-CoV-2 infection.

Both sequences (first SARS-CoV-2 infection and subsequent vaccination as well as vaccination and subsequent SARS-CoV-2 infection) are acceptable as long as the antibody thresholds defined below are met in the collected CCP units.

As far as the availability of CCP units allows, the two plasma units should have been donated by two different convalescents. Plasma should contain a minimum neutralising antibody titre of 1:640 against delta (B1.617.2), omicron, or any future variant as that would allow a titre of 1:100 to be achieved in an average recipient and this level is considered sufficient to neutralise the virus within host. Around 30% of vaccinated donors with a previous infection will demonstrate these antibody levels.

Before convalescent plasma is approved for clinical trial use, the SARS-CoV-2 antibodies should be measured from a donor prior to or during donation or from donated plasma after donation either using live virus neutralisation assay with delta variant, Elecsys Anti-SARS-CoV-2 S Assay (from Roche), Anti-SARS-CoV-2-QuantiVac-ELISA (IgG) (from Euroimmun) or other assay which have been demonstrated to predict the required titre level:

- Live virus microneutralisation assay: a minimum neutralising antibody titre of 1:640 against delta, omicron, or any future variant
  or
- Elecsys Anti-SARS-CoV-2 S (Roche): a minimum antibody concentration of 20.000 IU/ml
  Or
- Anti-SARS-CoV-2-QuantiVac-ELISA (IgG) (Euroimmun): a minimum antibody concentration of 4.000 BAU/ml
  or
- Any other assay shown to correlate with any of the above assays.

Lymphocytes isolated from a blood sample drawn from the pre-donation bag will be used for measuring anti-SARS-CoV-2 reactivity including variants.

Retention Plasma or a 5 mL serum sample from each convalescent plasma donation provided for clinical trial should be stored for further testing to allow comparison of data between countries and to analyze their cross-neutralization capacity against new viral variants which might evolve during the course of this trial.

As mentioned earlier, an Ab dose effect has been reported with CCP transfusion. The transfusion of 2 units of plasma (the same day or on 2 subsequent days) is the maximum amount of plasma one can reasonably transfuse early after COVID-19 symptoms initiation and in an outpatient setting. Such a dosage of CCP has been applied in several clinical studies and found to be well tolerated.[27,35]

Table 1. Procedures performed on convalescent plasma as part of the protocol

Procedures performed on convalescent plasma as part of the protocol
Anti-SARS-CoV-2 ELISA or live virus microneutralisation assay
Retention plasma or 5mL serum/plasma from donation

II-7. Patient population

Three populations of patients with symptoms of COVID-19 will be included in two cohorts. Cohort 1 will include patients aged 70 or over and patients under 70 with significant co-morbidities (arterial hypertension, diabetes, obesity, asthma or other chronic pulmonary disease, cardiovascular disease, cerebrovascular disease, chronic kidney disease/dialysis, liver disease, chronic neurological disease, rheumatoid arthritis, lupus or psoriasis) resulting in a “COVID-age” of 70 years or above according to the ALAMA risk calculator. Cohort 2 will include immunosuppressed patients (acquired or primary immune deficiency).

III. STUDY OBJECTIVES AND ENDPOINTS

III-1. Primary objective

The primary objective of the study is to evaluate the efficacy of plasma from COVID-19 convalescent donors (COVID-19 convalescent plasma) and standard of care versus standard of care alone in preventing hospitalisation or death by day 28 after randomisation, in clinically vulnerable patients recently infected by SARS-CoV-2.

Standard of care therapy may include anti-SARS-CoV-2 specific medication listed as authorized in a dedicated appendix.

III-2. Secondary objectives

The secondary objectives of the study are as follow:

To evaluate the efficacy of COVID-19 convalescent plasma on

1. Hospitalisation for severe COVID-19 by day 14 after randomisation
2. Hospitalisation for severe COVID-19 requiring O_2 support by day 14 and 28 after randomisation
3. All-cause mortality by day 28, 90 and 180 after randomisation
4. Requirement for supplemental oxygen by day 14 and 28 after randomisation
5. Requirement for non-invasive ventilation by day 14 and 28 after randomisation
6. Requirement for mechanical ventilation by day 14 and 28 after randomisation
7. The change in WHO Clinical Progression Scale score by day 14 and 28 after randomisation
8. The duration of hospital stay
9. The need for admission to ITU by day 14 and 28 after randomisation
10. The duration of ITU stay
11. Cases of long COVID-19 symptoms and the time to recovery
12. Health-related quality of life

To analyse the safety of COVID-19 convalescent plasma with regard to:

1. Serious adverse events (Grade 3/4 adverse events) and AE unexpected for their nature, onset, evolution, severity or frequency
2. Arterial and venous thrombotic events

III-3. Exploratory objectives

The study also aims to:

- Analyse the effect of convalescent plasma on SARS-CoV-2 viral load.
- Describe the course of anti-SARS-CoV-2 spike antibody levels in blood (provisional).
- Analyse the SARS-CoV-2 whole genome sequence.
- Determine whether persistently-infected immunosuppressed individuals are a source of infectious viral variants.
- Analyse the association between receipt of CCP and development of infectious SARS-CoV-2 viral variants.

III-4. Study endpoints

The primary endpoint is the proportion of participants with (1) at least one overnight stay in hospital for progressive COVID-19 symptoms, or (2) who died, by day 28 after randomisation. COVID19 related

hospitalizations will be adjudicated using a three member panel. Each member will independently come to a decision on whether the hospitalization or decision to extended a hospitalisation was or was not related to COVID-19 using as much information that could be provided such as hospital discharge forms but remain blinded for the randomization group. Classification of whether the hospitalization was due to COVID-19 will be by majority decision of the panel.

Secondary outcome measures include core outcomes from the meta-core outcome set (COS) for hospitalised patients (http://www.comet-initiative.org/Studies/Details/1538), and the WHO progression scale.[59] There is no COS for patients with COVID-19 managed in the community. Viral outcome measures will enable assessment of mechanism.

Secondary endpoints include:

Efficacy:

1. Proportion of participants with hospitalisation for progressive COVID-19 symptoms, or death by day 14 after randomisation (stages 4 to 9 of the WHO scale)
2. Proportion of participants with hospitalisation for progressive COVID-19 symptoms requiring O_2 support*, or death by day 14 and 28 after randomisation (stages 5 to 9 of the WHO scale)
3. All-cause mortality by day 28, 90 and 180 after randomisation
4. Proportion of patients with supplemental oxygen by day 14 and 28 after randomisation
5. Proportion of patients with non-invasive ventilation by day 14 and 28 after randomisation
6. Proportion of patients with intubation and mechanical ventilation by day 14 and 28 after randomisation
7. Change in 10-point WHO Clinical Progression Scale score by day 14 and 28 after randomisation (see below)
8. Duration of hospital admission censored at 28 days after randomisation (for participants reaching primary endpoint)
9. Proportion of patients with admission to ITU by day 14 and 28 after randomisation
10. Duration of ITU admission censored at 28 days after randomisation
11. Proportion of patients with long COVID-19 symptoms and time to recovery assessed by questionnaire at days 28 and 180 post randomisation
12. Health-related quality of life assessed using the EQ-5D-5L at 28 and 180 days after randomisation

* O_2 support requirement based on O_2 saturation level on room air ≤ 93 % and/or Respiratory Rate > 30

Safety:

13. Number of serious Adverse Events at 72 hours after randomisation (Grade 3/4 adverse events and AE unexpected for their nature, onset, evolution, severity or frequency)
14. Arterial and venous thromboembolic events at 28, 90 and 180 days after randomization

Exploratory endpoints:

1. Change in SARS-CoV-2 RNA level (Polymerase chain reaction, Cycle Threshold value) in oral or nose/throat swab samples at days 3, 14, 28, hospitalisation and 180 after randomisation (cohort 2 only)
2. Change in anti-SARS-CoV-2 spike antibody levels in blood at days 3, 14, 28 and hospitalisation after randomisation

3. SARS-CoV-2 whole-genome sequence analysis in oral or nose/throat swab samples at day 1, 28, hospitalisation and day 180 after randomisation
4. Proportion and clinical characteristics of patients with cultivable virus at day 28, hospitalisation and day 180
5. Virus sequence variation and cultivability over time, overall and in individuals receiving vs not receiving CP

Table 2. 10-point WHO Progression scale

Patient state	Descriptor	Score
Uninfected	Uninfected; no viral RNA detected	0
Ambulatory	Asymptomatic; viral RNA detected	1
	Symptomatic; independent	2
	Symptomatic; assistance needed	3
Hospitalised: moderate disease	Hospitalised; no oxygen therapy*	4
	Hospitalised; oxygen by mask or nasal prongs	5
Hospitalised: severe disease	Hospitalised; oxygen by NIV or high flow	6
	Intubation and mechanical ventilation, $pO_2/FiO_2 \geq 150$ or $SpO_2/FiO_2 \geq 200$	7
	Mechanical ventilation $pO_2/FIO_2 < 150$ ($SpO_2/FiO_2 < 200$) or vasopressors	8
	Mechanical ventilation $pO_2/FiO_2 < 150$ and vasopressors, dialysis, or ECMO	9
Dead	Dead	10

ECMO=extracorporeal membrane oxygenation. FiO2=fraction of inspired oxygen. NIV=non-invasive ventilation. pO2=partial pressure of oxygen. SpO2=oxygen saturation. *If hospitalised for isolation only, record status as for ambulatory patient.

IV. STUDY DESIGN

IV-1. Overall design

COVIC-19 is a multicentre international, randomised, open-label adaptive superiority phase III trial to evaluate the efficacy and safety of COVID-19 convalescent plasma in the treatment of COVID-19. It is conducted in a harmonized approach in different countries in Europe.

The study is randomizing adult COVID-19 patients to one of two arms (1:1 ratio): standard of care or standard of care and very high neutralizing Ab titre convalescent plasma. Randomization will be stratified by centre and by patient cohort. The control group will receive 'standard care' therapy. Neither blinding nor placebo will be used to avoid unnecessary intravenous access.

Standard of care therapy may include anti-SARS-CoV-2 specific medication listed as authorized in a dedicated appendix. Centres should ensure that medications used as standard of care are used similarly for patients in both treatment arms.

Participating patients will be included in 2 cohorts of vulnerable patients (cohort 1: unvaccinated elderly (≥ 70 years) and younger with comorbidities (cohort 1: < 70 with comorbidities), cohort 2: immunosuppressed patients).

All subjects will undergo a series of efficacy and safety assessments, including laboratory assays. Subjects will be assessed at baseline, and at Days 3, 14, 28, 90 and 180.

Nasopharyngeal swabs (NP) or lower respiratory tract samples will be obtained at D1 (pre-treatment), and at D3, D14, D28 and D180 (and monthly in case of positivity until of clearance) for cohort 2 and on the day of hospitalization (if applicable).

Blood samples will be obtained at D1, D14 and D28 and on the day of hospitalization (if applicable).

IV-2. Rationale for study design

The COVIC-19 study will include two distinct cohorts: firstly, unvaccinated elderly and younger patients with co-morbidities, and secondly, immunocompromised patients. This distinction is justified in particular by the differences between these two groups with regard to the characteristics of the COVID-19 disease, differences accentuated by the effects of the vaccination, significant in the 1st cohort, much less so in the 2nd cohort. The two cohorts will be analyzed separately.

The COVIC-19 study will be carried out in 2 stages:

• A 1st step with the objective of confirming or not the data of Libster et al[1] in each of the cohorts (50% reduction of a risk of a 30% aggravation rate) with a power of 90%, an alpha risk of 5% (and + 5% of additional patients). The number of patients required is 340 in each cohort, for a total of 680 patients.

• For each cohort, an unblinded interim analysis will be performed when 30% of the patients of the first stage reach the primary endpoint [102 patients]. The aim of this analysis is to reestimate the sample size of the trial. If the conditional power for detecting the difference in primary outcome between the two arms in the final analysis is between 50% and 90%, the sample size will be increased in order to achieve 90% power to detect the effect observed at the interim analysis, with a maximum sample size of 1020 per cohort. Otherwise, the trial will continue using the planned sample size.

It was decided to conduct an open trial because transfusion of plasma without neutralizing antibodies generates a risk without any benefit for the placebo arm.

IV-3. Study duration and dates

The anticipated duration of recruitment is 12 months for step 1 and 12 to 24 months for step 2. The duration of participation of each patient is 6 months.

The total duration of the study is 18 to 42 months.

V. STUDY POPULATION

V-1. Inclusion criteria

Two patient populations will be included in the study: (1) unvaccinated elderly and high COVID-age population and (2) high-risk immunocompromised population.

V-1-1. Unvaccinated elderly and high COVID-age population:

1. SARS-CoV-2 RNA detected in a specimen, or positive antigenic test, ≤ 7 days after onset of symptoms
2. Symptoms of COVID-19 (so including but not limited to: fever; cough; breathlessness; chest pain; wheeze; sore throat; haemoptysis; runny nose; fatigue; muscle or joint pain; confusion; headache; seizures; nausea; vomiting; diarrhoea; abdominal pain; poor appetite; skin ulcers or rash; ear pain; conjunctivitis; anosmia; bleeding; lymphadenopathy).[60] The attending clinician will determine if symptoms are consistent with COVID-19.
3. Clinical status not requiring admission to hospital for COVID-19 disease and oxygen support
4. Ability to transfuse (per randomisation) within 7 days after onset of symptoms
5. Men or women, 70 years or older
   OR
   under 70 years with significant comorbidities (arterial hypertension, diabetes, obesity, asthma or other chronic pulmonary disease, cardiovascular disease, cerebrovascular disease, chronic kidney disease / dialysis, liver disease, chronic neurological disease, rheumatoid arthritis, lupus or psoriasis) resulting in a 'COVID-age' of 70 years or more according to the ALAMA risk calculator https://alama.org.uk/covid-19-medical-risk-assessment/
6. Signed written informed consent

V-1-2. High-risk immunocompromised population

1. SARS-CoV-2 RNA detected in a specimen, or positive antigenic test, ≤ 7 days after onset of symptoms
2. Symptoms of COVID-19 (so including but not limited to: fever; cough; breathlessness; chest pain; wheeze; sore throat; haemoptysis; runny nose; fatigue; muscle or joint pain; confusion; headache; seizures; nausea; vomiting; diarrhoea; abdominal pain; poor appetite; skin ulcers or rash; ear pain; conjunctivitis; anosmia; bleeding; lymphadenopathy.[60] The attending clinician will determine if symptoms are consistent with COVID-19.
3. Clinical status not requiring admission to hospital for COVID-19 disease and oxygen support
4. Ability to transfuse (per randomisation) within 7 days after onset of symptoms
5. Male or female with extremely high risk including:
   a. Patients with at least one of the following acquired immune deficiencies:
      i. Lymphoid malignancies treated within the last 12 months
      ii. Lymphoid malignancies with persistent hypogammaglobulinaemia (IgG < 5g/L)
      iii. Myeloid malignancies treated by chemotherapy within the last 12 months
      iv. Myeloid malignancies treated by anti-BCL-2 drugs within the last 12 months
      v. Myeloid malignancies associated with prolonged neutropenia (≥ 6 weeks)
      vi. Solid tumour undergoing treatment with chemotherapy (until 3 months after completion of the last chemotherapy cycle).
      vii. Allogenic hematopoietic stem cell transplantation within the last 12 months or anytime if on-going treatment for chronic GVHD
      viii. Organ transplantation

ix. Anti - B (CD20/CD19) MoAb and/or mycophenolate mofetil treatment within the last 12 months
x. Anti-CD19/CD20 CAR-T cell treatment
xi. ATG or alemtuzumab treatment within the last 6 months
xii. AIDS

or

b. Patients with primary lymphoid immune deficiencies:
   i. B cell deficiencies (such as Bruton agammaglobulinemia)
   ii. T cell deficiencies (such as Wiskott Aldrich disease)
   iii. Combined deficiencies (such as Common variable immunodeficiency)

Or

c. Patients without detectable seroconversion ≥ 3 weeks after complete vaccination schedule with an approved vaccine.

6. Signed written informed consent

V-2. Exclusion criteria

V-2-1. Unvaccintaed elderly and high COVID-age population:

1. Age < 18 years
2. Prior or concurrent treatment for COVID-19 (unless listed as authorized specific treatment)
3. History of documented SARS-CoV-2 infection in the last 90 days prior to enrolment
4. Prior anti-SARS-CoV-2 immunization
5. Contraindication to receiving CCP including previous history of transfusion-related acute lung injury (TRALI) or moderate or severe allergic reaction to blood components
6. Known participant objection to receiving plasma products
7. Primary or acquired immune deficiency listed below (see cohort 2)
8. Refusal to participate expressed by patient or legally authorised representative
9. Pregnancy

V-2-2. High-risk immunocompromised population

1. Age < 18 years (except for UK)
2. Prior or concurrent treatment for COVID-19 (dexamethasone, anti-IL-6/IL6R, remdesivir) except for prophylactic administration of anti-SARS-Cov2 monoclonal antibodies (pre or post exposure) in cohort 2 and authorized specific treatment
3. History of documented SARS-CoV-2 infection in the last 90 days prior to enrollment
4. Contraindication to receiving CCP including previous history of transfusion-related acute lung injury (TRALI) or moderate or severe allergic reaction to blood components
5. Known participant objection to receiving plasma products
6. Refusal to participate expressed by patient or legally authorised representative
7. Pregnancy

Any patient eligible for both cohorts will be included in cohort 2.
Enrolment in other trials after reaching the primary end-point is authorised.

VI. STUDY ASSESSMENTS AND PROCEDURES

VI-1. Screening Assessment

Patients will be approached via their direct care team in hospitals, nursing homes, or General Practice in the following scenarios where symptoms compatible with COVID-19 are present: (1) Those attending routine appointment, (2) Those receiving unscheduled care in emergency or acute assessment units, but not requiring admission for COVID-19 (3) Those who contact their hospital, community team, or

family practitioner reporting symptoms (4) High-risk asymptomatic patients who are screened for SARS-CoV-2 as part of routine care, in line with current clinical guidelines. Those with a positive virological result will become eligible on reporting symptoms.

VI-2. Screen Failures

After the screening evaluations have been completed, the investigator or his/her designee is to review the inclusion/exclusion criteria and determine the subject's eligibility for the study. Only the reason for ineligibility will be collected on screen failures. Subjects who are found to be ineligible will be told the reason for ineligibility.

VI-3. Baseline/ Follow-up

Baseline and follow-up assessments will be organised on Days 1 pre-treatment, 3, 14, 28, 90 and 180 (Table 3). Patients will complete a patient questionnaire and will be contacted by phone. Participants randomised to standard care, or whose care allows it, will have blood samples taken in a health clinic or at home and any follow-up blood, respiratory or saliva samples will be self-sampled or performed by a care nurse.

***Table 3**: Table of assessments*

Day<br>+/- window (days)<br><br>*Name of visit*	Screening<br>Up to 7 days prior to D1	Baseline<br><br>*D1*	D3<br>+ 2<br><br>*FU 1*	D 14<br>± 2<br><br>*FU2*	D 28<br>± 2<br><br>*FU 3*	D X[5]<br>Hospitalization/O_2 + 2<br><br>*Hospitalization*	D90<br>± 7<br><br>*FU 4*	D 180<br>± 14<br><br>*FU 5*
Eligibility criteria	X							
Informed consent	X							
Review SARS-CoV-2 results	X							
Review ABO group results	X							
**Study intervention**								
Randomization		X[7]						
CCP administration (only in treatment arm)		X						
**Study procedures**								
Demographics and Medical History	X							
Examination / Vital signs		X				X		
Symptom recording	X	X	X	X	X	X		
Vital status		X			X	X	X	X
Concomitant medications	X	X	X	X	X	X		
Hospitalisation/ITU		X	X	X	X	X		
Ventilation mode				X	X	X		
Oxygenation				X	X	X		
Clinical Progression Scale		X	X	X	X	X		
QoL and long COVID Questionnaires		X			X			X
Adverse event recording		X	X	X	X	X	X	X
**Laboratory analyses**								
SARS CoV 2 RT-PCR in nose and throat swabs/saliva	X[1, 8]	X	X[2]	X[2]	X[2]	X		X[2]

SARS CoV 2 sequencing (whole genome sequencing)	X[8]	X			X[6]	X[6]		X[6]
SARS CoV 2 isolation from nose and throat swab/saliva (cohort 2 only, optional)		X[6,8]			X[6,8]	X[6,8]		X[6,8]
SARS CoV 2 antibody in serum	X	X	X[8]	X[2]	X[2]	X		
Biochemistry bloods[3]		X		X[8]		X		
Haematology bloods[4]		X		X[8]		X		
Pregnancy test (in females of childbearing potential only)	X							
Serum for biobank (optional)		X[8]		X[8]	X[8]	X[8]		
Plasma for biobank (optional)		X[8]		X[8]	X[8]	X[8]		
EDTA sample (for isolation of immune cells) for biobank (optional)		X[8]		X[8]	X[8]	X[8]		

[1] For individuals with no RT PCR or Antigenic test result in the preceding 7 days
[2] Cohort 2; in cohort 1 only if the patient´s have not been tested negative in the SARS-CoV-2 PCR before.
[3] Urea, Electrolytes, Liver Function Tests (ALT, AST), Creatinine, calcium, phosphate, glucose, C-reactive protein (CRP). LDH, ferritin, IL-6 (optional)
[4] Full Blood Count (neutrophils/total lymphocyte counts, platelets), Prothrombin, Activated partial thromboplastin time (aPTT), fibrinogen, D dimer, G&S
[5] Visit "hospitalization" on the day of hospitalization and/or start of supplementary O2 (+ 2 days) (only if this event occurs up to day +28).
[6] if viral load still sufficient to perform sequencing or virus isolation
[7] between screening and administration of CCP (D1)
[8] not mandatory

VI-4. Data recorded

VI-4-1. Baseline information

The following information will be recorded on the web-based form by the attending clinician or delegate:

Data recorded	Source of the data
Medical history including High risk categories/major comorbidity	Patient and medical records
Patient details (age, sex…), place of residence, weight, height, BMI, Vital signs	Medical records
SARS-CoV-2 vaccination (dates, type of vaccine)(cohort 2 patients)	Patient and medical records
COVID-19: COVID-19 symptom onset date, Date of SARS-CoV-2 RNA detection, symptoms details, infection history, COVID-19 severity as assessed by WHO score	Patient and medical records
Concomitant medication (including use of any other medication to treat COVID-19 as standard of care)	Patient and medical records
ALAMA risk score	Medical records
Biological data	Medical records

VI-4-2. Follow up information

The following information will be ascertained at each visit, as well as at time of death or at 28 days after first randomisation (whichever is sooner):

- Vital status
- Hospitalisation status
- 10-point WHO progression scale
- Oxygen supplementation and/or ventilation
- Use of renal dialysis or haemofiltration
- Documented new major cardiac arrhythmia (including atrial and ventricular arrhythmias)
- Use of any other medications to treat COVID-19
- AE / SAE / SAR / SUSAR

This information will be obtained and entered into the web-based IT system by a member of the hospital clinical or research staff.

Follow-up information is to be collected on all study participants, irrespective of whether or not they complete the scheduled course of allocated study treatment. Study staff will seek follow-up information through various means including medical staff, reviewing information from medical notes, routine healthcare systems, and registries.

VII. STUDY PRODUCT

Details are available in investigator brochure and product circuit.

VII-1. Investigational therapeutic

The investigational medicinal product is composed of two plasma units provided by two COVID-19 convalescent patients, fully compliant with national regulations as detailed in the investigator's brochure.

The first unit of ABO compatible convalescent plasma (200 to 350 ml per unit) will be infused intravenously on study day 1 (as soon as possible after randomisation) and the second on day 1 or day 2. Plasma has been obtained by apheresis from donors who have recovered from COVID-19 infection (at least 14 days after recovery) and have been vaccinated (at least 3 weeks after first dose of vaccine). A combination of both a SARS-CoV-infection and a SARS-CoV-2 vaccination of the donor is required – irrespective of the sequence of infection and vaccination. As far as the availability of CCP units allows, the two plasma units should have been donated by two different convalescents.

VII-2. Convalescent plasma collection

Plasma units are obtained, prepared and delivered in strict observance of the existing rules, as part of the usual activity of the structures.

Briefly, convalescent donors at least 14 days after the symptoms resolution will undergo plasma apheresis. The convalescent donors will undergo standard pre-donation assessment to ensure compliance with current regulations regarding plasma donation including standard microbiological assessment, as well as anti-HLA Ab detection in women with children.
An anti-SARS-Cov-2 spike IgG Ab titre should be performed. Only CCP with a minimum antibody titre as defined in II-6 will be used.

The following information about immunization events will be collected from donors: date of first SARS-CoV-2 infection, SARS-CoV-2 variant (if variant has been identified), date of SARS-CoV-2 vaccinations, and name of vaccine and data of SARS-CoV-2 breakthrough infection (if such an event happened).

VII-3. Storage, Handling, Distribution and Stability

The component should be stored at a core temperature of –25°C or below (storage period in agreement with national regulations). Although a storage temperature below –25°C improves the preservation of labile coagulation factors, lower temperatures increase the fragility of plastic. Particular care must be taken when handling such packs.

The component should be thawed in equipment designed for the purpose, within a vacuum-sealed overwrap bag according to a validated procedure. The optimal temperature at which the component should be thawed is 37°C; temperatures between 35°C and 39°C are acceptable.

Protocols must be in place to ensure that the equipment is cleaned daily and maintained to minimise the risk of bacterial contamination. After thawing, and at the time of administration, the content should be inspected to ensure that no insoluble cryoprecipitate is visible and that the container is intact.
Once thawed, the component must not be refrozen and should be transfused as soon as possible. If a delay is unavoidable, the component may be stored and should be used within 6 hours.

Process for issuing each unit of convalescent plasma is as follows:

- Unit of ABO compatible convalescent plasma should be thawed as per normal blood bank procedures (ABO matched). Preferentially ABO identical units should be used.
- Convalescent plasma should be transferred to the patient following local procedures
- The two units of plasma should be collected from two different donors.

VII-4. Administration

Two convalescent plasma units of 200 to 350 ml will be transfused i.v. in patients from the CCP arm, on one or two days.

Process for administration of convalescent plasma is as follows:

- All administration bedside transfusion safety checks must be undertaken.
- The donation number, volume transfused, and start and finish date and time of transfusion should be documented.
- Any suspected serious adverse reaction to transfusion must be reported to the transfusion laboratory as well as being reported on trial documentation.

Convalescent plasma will be administered by staff trained to give transfusions of blood components and have been approved to do so per applicable regulation. All participants must be under surveillance during the infusion and should be monitored closely for 2 hours after the infusion. Plasma transfusion per- and post-transfusion surveillance as well as traceability and haemovigilance will be fully compliant with current regulations.

In hospital, CCP will be administered in day units. The exact logistics will vary depending on each hospital’s layout. Home transfusion, as well as transfusion in long-term care facilities or in health centers with dedicated personal, will be considered as well.

VII-5. Appearance

Clear to slightly cloudy, homogeneous liquid, without visible signs of haemolysis, contained in a bag.

VII-6. Justification for dose

A anti-SARS-CoV-2 IgG dose dependent effect has been observed in several studies.[1,39,40] The selection of donors based on their immunization history (infection and vaccination) and the pre-defined minimum concentrations of anti-SARS-CoV-2 antibodies in the CCP units for this trial will provide a standardized high-dose of anti-SARS-CoV-2 antibodies administered in this trial.

VII-7. Authorised medications

The list of authorised medications is detailed in a dedicated appendix. The trial scientific committee may update this list whenever necessary to ensure that trial participants receive state of the art standard of care at the time of inclusion. Occasions for a review of this list of authorized COVID-19 specific treatments include, but are not limited to, approval of new specific COVID therapeutics or changes in National Guidelines for patient populations that are also eligible for this study.

An update of this Appendix of Authorized Treatments will not be classified as a Protocol Amendment as long as the above conditions are met.

VII-8. Discontinuation of study intervention

Definition: Premature discontinuation of treatment = corresponding to the discontinuation of treatment with the investigational drug.

VII-8-1. Procedures for replacing these people, if applicable

Not applicable

VII-8-2. Procedures for monitoring these people

Standard of care

VII-9. Participant discontinuation/withdrawal from the study

If a subject is withdrawn before completing the study, the reason for withdrawal will be entered on the appropriate eCRF. Whenever possible and reasonable, the evaluations that were to be conducted during the final study visit should be performed at the time of premature discontinuation.
It is vital to obtain follow-up data on any subject who terminated because of an AE. In any case, every effort must be made to ensure safety follow-up procedures are completed.

A decision by a participant (or their parent/guardian) that they no longer wish to continue receiving study treatment should not be considered to be a withdrawal of consent for follow-up. However, participants (or their parent/guardian) are free to withdraw consent for some or all aspects of the study at any time if they wish to do so. In accordance with regulatory guidance, de-identified data that have already been collected and incorporated in the study database will continue to be used (and any identifiable data will be destroyed). For participants who lack capacity, if their legal representative withdraws consent for treatment or methods of follow-up then these activities would cease.

VII-10. Study discontinuation

At any time, the investigator as well as the sponsor reserve the right to prematurely interrupt the trial for medical and / or administrative reasons. This will only take place after mutual consultation. The reasons for the stop must be properly documented and the stop of the test must be notified to the competent authorities concerned.

VIII. MEASURES TO MINIMISE BIAS

VIII-1. Randomisation

In addition to receiving usual care, eligible patients will be allocated using a central web-based randomisation service (CleanWeb, Telemedicine Technologies, Boulogne Billancourt, France) available at https://chrub.tentelemed.com.

The randomisation service will allocate the treatment based on a pre-specified randomization list generated by the data manager. Separate randomisation lists will be used for each patient population. Randomisation will be performed at a 1:1 ratio, blocked (with randomly varying block sizes of two and four) and stratified by centre. Once randomised, patients are irrevocably enrolled in the study, whether or not they are subsequently found to be eligible or actually receive the allocated treatment. As a consequence, patients should be followed until their last visit, until stopping rules apply or death.

VIII-2. Maintenance of group comparability

The standard of care for COVID-19 is evolving rapidly. We will take specific measures to ensure that groups the standard of care used in participating patients remain comparable in both arms over the inclusion period. Whenever necessary, the trial steering committee will provide updated guidelines regarding the standard of care and will strongly recommend that participating centres observe them.

These recommendations may consider the local availability of COVID-19 specific medications such as MoAbs.

The primary endpoint is COVID-19 related hospitalisation or death by day 28. Hospitalisation or the decision to extend a hospitalisation may depend on local practice and availability of health service at the time of hospitalisation. In order to minimise bias on this endpoint, we will set up a three member panel to adjudicate whether hospitalisation or the decision to extend hospitalisation was COVID-related or not. Each member will independently come to a decision on whether the hospitalization was or was not related to COVID-19 using as much information that could be provided such as hospital discharge forms. Classification of whether the hospitalization was due to COVID-19 will be by majority decision of the panel.

Reasonable efforts will be made to keep the panel blinded for the randomisation group while also providing accurate information about the patient. As a general rule, information about the intervention will be removed from the discharge forms when possible, but clinical data about potential transfusion reactions and adverse events will not.

IX. Safety reporting

IX-1. Criteria for the evaluation of safety

IX-1-1. Identified safety data

- Serious adverse transfusion reactions (SAR) expected:

- Acute allergic / anaphylactic transfusion reaction: rash, angioedema, bronchospasm, hypotension;
- Transfusion associated acute lung insufficiency (TRALI);
- Transfusion associated respiratory overload, sudden worsening in respiratory status, acute lung injury, transfusion related dyspnea
- Acute clinical haemolysis, defined as fall in haemoglobin plus one or more of the following: rise in lactate dehydrogenase (LDH), rise in bilirubin, positive direct antiglobulin test (DAT), or positive crossmatch;
- Transfusion transmitted infection: bacterial, viral or fungal;
- Temperature >39°C or ≥2°C rise above baseline;
- Post-transfusion purpura
- Sudden hypotension : leading to shock, sudden drop in systolic blood pressure of ≥30 mmHg with systolic blood pressure ≤80 mmHg or requiring urgent medical attention

- Serious adverse events (SAE) expected:

<u>As the convalescent plasma will be used as a therapy, the patients could experience the following COVID complications:</u>

- Hospitalisation for progressive COVID-19
- Death for progressive COVID-19

IX-1-2. Regulatory definitions.

- **Definition of an Adverse Event**

Any harmful manifestation occurring in a person who lends itself to research involving the human person, whether or not this manifestation is related to research or to the investigational drug (s) on the subject (s) of this research.
If an adverse event consists of several signs or symptoms that may be represented by a single syndrome or a diagnosis, the syndrome or diagnosis will be recorded in the observation book as an adverse event, replacing the individual signs and symptoms.
Any event occurring after the signing of the consent and until the end of the subject's participation in the study will be considered as an adverse event.
Signs, symptoms, syndromes or diagnoses present at the time of signing the consent will be considered adverse events if they deteriorate after signing the consent.

- **Definition of a Serious Adverse Event (SAE)**

SAE is an AE that, at any time and any dose, fulfills one or more of the following criteria:

- **Results in death,**
- **Is life threatening**, i.e. the patient was at immediate risk of death at the time of the event; it does not refer to an event which might have caused death if it was more severe,
- **Requires in-patient hospitalization or prolongation of existing hospitalization**, i.e. hospitalization signifies that the patient has been detained, usually involving at least an overnight stay,
- **Results in persistent or significant disability/incapacity,** i.e. substantial disruption of a person’s ability to carry out normal life functions,
- **Is a congenital anomaly/birth defect,**
- **Is any important medical event** that may not be immediately life threatening or result in death or hospitalization but, based upon appropriate medical judgment, may endanger the patient or may require intervention to prevent one of the other outcomes listed in the definition above.
- **Suspected transmission of an infectious agent** (e.g., pathogenic or nonpathogenic) via the study drug is considered a SAE.

Any component of a study endpoint that is considered related to study therapy should be reported as an SAE (e.g., death is an endpoint, if death occurred due to anaphylaxis, anaphylaxis must be reported).

a) <u>Death</u>

SAE/SAR resulting in death should be fully documented and reported, even if death occurs after discontinuation of treatment, regardless of the causal relationship between the death and the product under study. If the cause cannot be determined, the death will be considered unexplained.

b) <u>Life-threatening event</u>

This does not apply to adverse reactions which, in a more severe form, could have resulted in death.

c) <u>Disability</u>

A substantial disruption in a person's ability to lead a normal life.

d) <u>Hospitalisation</u>

Certain circumstances requiring hospitalisation do not fall under the seriousness criterion: "hospitalisation/prolongation of hospitalisation" such as:
- Admission for social or administrative reasons;
- Hospitalization predefined by the protocol;
- Hospitalization for medical or surgical treatment scheduled before the research;
- The passage in the day hospital;
- A visit to the emergency room or other hospital department, that does not result in admission (unless considered an important medical or life-threatening event);
- Routine health assessment requiring admission for baseline/trending of health status (eg, routine colonoscopy)

e) Special cases:

Any pregnancy that begins or becomes apparent during the study must be notified to the Sponsor without delay via the pregnancy declaration form, even if the mother is the spouse of the exposed patient (paternal exposure).
The investigator should follow the patient until the pregnancy is terminated or terminated and notify the Sponsor of the outcome.
If the outcome of the pregnancy falls within the scope of the definition of serious adverse events (spontaneous abortion with hospitalization, foetal death, congenital anomaly, etc.) the investigator must follow the procedure for reporting SAEs.
If this is a paternal exposure, the investigator must obtain the consent of the parturient to collect information about the pregnancy.

- **Definition of a serious adverse transfusion reaction (SAR) of an investigational medicinal product**

Any harmful and unwanted reaction in a donor or recipient associated with the administration of blood or blood component.

- **Definition of a serious incident**

Any incident or error susceptible to affect the safety or the quality of blood or blood component and to cause adverse effects. It can be related to any step of the blood transfusion chain : blood collection, biological qualification of the blood donation, preparation, conservation, transport, distribution, dispensation, realization of pre-transfusion analyses, use of labile blood product, delay or lack of their transfusion.
Dysfunctions related to information systems and to patients identification are also incidents if they are susceptible to affect the products' safety or quality and to cause adverse reactions.
An incident is qualified as serious when susceptible to cause serious adverse effects.

- **Definition of an Unexpected Adverse Event**

Unexpected side effect is any side effect of the product, nature, severity, frequency or course of which does not agree with the reference safety information given in the summary of product characteristics or in the investigator brochure for the product when the product is not authorized (see chapter IX Undesirable effects).

- **Definition of a new fact**

Any new data that may lead to a reassessment of the ratio of benefits and risks of the research or of the product being researched, to modifications in the use of this product, in the conduct of research, or of documents relating to the research, or to suspend or interrupt or modify the research protocol or similar research.

IX-2. Methods and schedule for measuring, collecting and analysing these parameters (details in appendix)

IX-2-1. Intensity of an AE

It will be evaluated as follows:

- Mild : minimal inconvenience without affecting everyday activities;
- Moderate: Sufficient inconvenience to affect daily activities;
- Severe: Inability to perform daily activities;
- Death

IX-2-2. Causal relationship between AEs and the study procedures

IX-3. Procedures for Adverse Events

IX-4. Data Safety and Monitoring Board

The Data Safety and Monitoring Board (DSMB) is an advisory committee responsible for advising the Sponsor on the benefit / risk ratio and the conduct of a clinical trial.
The role, composition and functioning of the DSMB are detailed in the DSMB operating charter.

IX-5. Methods and duration of follow-up following the occurrence of an adverse event (details in appendix)

X. STATISTICAL METHODS

X-1. Analysis populations

X-1-1. Flow diagram

At the final analysis of trial, a flow chart will be constructed according to the CONSORT 2010 reporting guidelines. It will describe:

- The number of eligible patients, randomized patients and the number of patients who have actually followed the study;
- The intervention arm allocated per randomisation;
- Early cessation of the intervention and their causes and drop-outs;
- The number of patients excluded from the analysis.
- The number of randomized but ineligible patients, if any, will also be reported, as well as the reason for ineligibility.

X-1-2. Intention to treat population

The analyses will be performed according to the intention-to-treat (ITT) principle. The ITT population (by patient cohort) will be used for all efficacy analyses. This will consist of all randomised patients according to their allocated arm irrespective of whether the patient actually received study drug or the patient's compliance with the study protocol, in the treatment group assigned by the randomization.

The list of protocol deviations will be established and the reasons for non-evaluable cases will be indicated as following:

- Inclusion criteria not satisfied.
- Deviations related to the study drug administration

All randomized patients will be included in the primary ITT efficacy analysis.

A modified intention to treat population (mITT) will be considered. The modified ITT population will exclude ineligible patients who were randomized in error.

X-1-3. Safety analysis population

All patients who received convalescent plasma will be included in the Safety analyses.

X-2. Sample size determination

The Sample size of the study is based on the risk of severe COVID-19 and the effect previously observed by Libster et al.[1] in an elderly high-risk population and a similar intervention of early convalescent plasma. In this study, 31% of the control group patients experienced severe COVID-19 disease, the relative risk of severe COVID-19 in treated patients was 0.52 (95% Confidence Interval 0.29–0.94). We estimate that the risk of severe COVID-19 in the elderly population will be 30% and the relative risk reduction in the treated group will be 0,5.

For a cohort, group sample sizes of 158 in group 1 and 158 in group 2 achieve 90% power to detect a difference between the group proportions of -0.15. The proportion in group 1 (the treatment group) is assumed to be 0,30 under the null hypothesis and 0.15 under the alternative hypothesis. The proportion in group 2 (the control group) is 0.30. The test statistic used is the two-sided Z-Test. The significance level of the test is 0.05. The sample size is increased to 170 to account for missing data/loss to follow up.

Interim analysis and sample size reestimation

The SARS-CoV-2 pandemic is a constantly evolving situation and with the appearance of new variants, there is considerable uncertainty as to the real risk of severe COVID-19 in the population. There is little data regarding the risk of severe COVID-19 in the immunocompromised population. Moreover, the effect observed by Libster et al. may be overly optimistic by chance and may be different in our trial because of local variations in donor selection and plasma production methods. If the effect is overestimated, the trial may not achieve the desired power. Thus, we will include a sample size reestimation during the course of the trial as a form of adaptive design.

For each cohort, sample size re-estimation will be planned using the method proposed by Mehta and Pocock.[61] Specifically, for each cohort, an interim analysis will be conducted when 30% of the patients, have reached the primary endpoint assessment. The conditional power for detecting the difference in primary outcome between the two arms in the final analysis will then be estimated. If the conditional power is between 50% and 90%, the sample size will be increased in order to achieve 90% power to detect the effect observed at the interim analysis with a maximum sample size of 1020 per cohort. Otherwise, the trial will continue using the planned sample size.

The table below presents the conditional power and estimated effect at interim analysis for values of Z1 between which sample size will be increased.

Z at interim analysis	Conditional power	Estimated effect at interim analysis (risk difference)	Estimated effect at interim analysis (relative risk)
1.07	0.50	-0.09	0.66
1.66	0.90	-0.14	0.52

The interim analysis will be performed by an independent statistician and presented to the DSMB. Decision boundaries are non-binding, and the DSMB can recommend continuing the trial at the pre-planned sample size.

Sample size reestimation boundaries and the size of the sample size increase were produced using the gsDesign package v. 3.2 (K. Anderson, Merck research laboratories, Boston, MA) with R version 4.0.5 (The R Foundation for Statistical Computing, Vienna, Austria).

X-3. Statistical analysis

General principles

All analyses for reports, presentations and publications will be prepared by the coordinating centre at the Research Methods Unit (uMETh) of the Clinical Investigation Centre, University Hospital of Besançon. A more detailed statistical analysis plan will be developed by the investigators whilst still blind to any analyses of aggregated data on study outcomes by treatment allocation.

Although conducted as a single trial with randomisation stratified on the groups of patients (cohort 1 and 2) for operational and logistical reasons, the findings in each cohort are considered as separate trials and are analysed separately.

The final results will be reported according to the recommendations of CONSORT 2010.

All outcomes will be analysed in superiority (two-sided) analyses using simple hypothesis tests. As sensitivity analyses, generalized linear models will be used to account for stratification on the centre. No correction for multiplicity and no hierarchical testing procedures are planned in analysing secondary outcomes. These analyses will therefore be considered as exploratory in nature.

Handling of missing or incoherent data

In the case outcomes would be missing, binary missing outcomes will be treated as treatment failures in interim and primary final analyses, with an imputation by last value carried forward as a sensitivity analysis. For time-to-event outcomes, they will be naturally handled using methods for censored data. No imputation will be used for secondary efficacy and safety outcomes.

Statistical software

The analyses will be carried out using the R software version 4.0.5 or later (The R Foundation for Statistical Computing, Vienna, Austria) and SAS version 9.4 or later (SAS Institute Cary, NC).

Characteristics at inclusion

The characteristics of patients collected at inclusion will be described globally and by randomization group, using means, standard deviations, medians, interquartile intervals, minimum and maximum for quantitative variables and by their numbers and percentages by modality for qualitative variables. The number of missing data for each variable will also be reported. No statistical tests for comparison between groups will be carried out.

Primary endpoint analysis

Primary endpoint definition: the proportion of patients with (1) at least one overnight stay in hospital for progressive COVID-19 symptoms, or (2) who died by day 28 after randomisation.

The primary outcome will be analysed using a two-sided Z-test to compare proportions of events in the randomisation groups. The risk difference will be provided with its 95% confidence interval. As a sensitivity analysis accounting for stratification on the centre, the endpoint will be analysed using a generalized linear mixed models (with logit link) with a random centre effect. Adjustment for major prognostic factors will be considered depending on the evolution of medical knowledge on the prognosis of patients infected by SARS-Cov-2.

Chen et al. have shown that if one increases the sample size only when the interim result is promising, the type-1 error is not inflated by use of the conventional Wald statistic. The significance level of the primary analysis will therefore remain at 0.05.

Subgroup analyses

At the end of the study, subgroup analyses for the primary endpoint will be performed according to SARS-CoV-2 vaccinal status at inclusion and the cumulative dose of antibody received. The interactions

between experimental treatment and vaccinal status as well as the dose of antibody received will be explored and tested

Secondary endpoints analysis

Secondary endpoints include the following:

- Proportion of hospitalisation for progressive COVID-19 symptoms, or death by day 14 after randomisation
- Proportion of hospitalisation for progressive COVID-19 symptoms requiring O2 support, or death by day 14 and 28 after randomisation
- All-cause mortality by day 28, 90 and 180 after randomisation
- Proportion of patients with supplemental oxygen by day 14 and 28 after randomisation
- Proportion of patients with non-invasive ventilation by day 14 and 28 after randomisation
- Proportion of patients with intubation and mechanical ventilation by day 14 and 28 after randomisation
- Change in 10-point WHO Clinical Progression Scale score at 14 and 28 days after randomisation
- Duration of hospital admission censored at 28 days after randomisation (for participants reaching primary endpoint)
- Proportion of patients with admission to ITU by day 14 and 28 after randomisation
- Duration of ITU admission censored at 28 days after randomisation
- Proportion of patients with long COVID-19 symptoms and time to recovery assessed by questionnaire at days 28 and 180 post randomisation
- Health-related quality of life assessed using the EQ-5D-5L at 28 and 180 days after randomisation

Binary outcomes will be analysed using Z-tests or Fisher's exact tests. When appropriate Risk differences will also be provided with their 95% confidence intervals. As a sensitivity analysis, generalized linear mixed models (with logit link) with a random centre effect will be performed. Time-to-event outcomes will be analysed using log-rank tests with sensitivity analyses based on Cox regression models with a random centre effect (results will be expressed as hazard ratios with 95% confidence interval). Finally, quantitative outcomes will be analysed using Student's t-tests or Mann-Whitney tests, as appropriate. Sensitivity analyses will consist in mixed linear regression with a random centre effect (results will be expressed as mean differences with 95% confidence interval).

Health-related quality of life

Health-related quality of life will be assessed using the EQ-5D-5L questionnaire. For health-related quality of life questionnaires, descriptive statistics (n, mean, standard deviation, median, minimum and maximum) will be summarised by time point and treatment group. Actual values and changes over time in scores will also be summarised.

Safety analysis of intervention

Safety endpoints include the following:

- Number of serious Adverse Events at 72 hours after randomisation (Grade 3/4 adverse events and AE unexpected for their nature, onset, evolution, severity or frequency)
- Arterial and venous thromboembolic events at 28, 90 and 180 days after randomization

Adverse events and their characteristics will be described using numbers and percentages per treatment arm. The proportion of participants with each of the reported events, as well as the

proportions of participants with at least one SAE will be compared using Fisher's exact tests. The total number of AE/SAEs and SAEs will also be described for each arm, and compared using Poisson models (with a robust error variance if necessary).

Exploratory outcomes

Exploratory outcomes include the following:

- Change in SARS-CoV-2 RNA level (Polymerase chain reaction, Cycle Threshold value) in oral or nose/throat swab samples at days 3, 14, 28 and 180 after randomisation (cohort 2 only)
- Change in anti-SARS-CoV-2 spike antibody levels in blood at days 14 and 28 after randomisation
- SARS-CoV-2 whole-genome sequence analysis in oral or nose/throat swab samples at day 1 and 28 after randomisation
- Proportion and clinical characteristics of patients with cultivable virus at day 28, hospitalisation and day 180
- Virus sequence variation and cultivability over time, overall and in individuals receiving vs not receiving CP

Summaries for biomarkers (i.e., exploratory endpoints) will be provided by treatment group and collection time. Additional exploratory analyses may be performed and will be specified in the SAP, as appropriate.

Subgroup analyses

Exploratory analyses of safety and efficacy will be conducted in subgroups of subjects including but not limited to SARS-CoV-2 variant identified at inclusion, SARS-CoV-2 vaccinal status at inclusion, anti-SARS-CoV-2 MoAbs received, and the cumulative dose of antibody received. The interactions between experimental treatment and vaccinal status as well as the dose of antibody received will be explored and tested.

X-4. Degree of significance

All tests will be two-sided, with p values of 0.05 or less denoting statistical significance.

X-5. Stopping rules

- Newly emerging effective treatment for COVID-19 which would substantially change the risk benefit assessment of the investigational approach in this trial compared to alternative options
- Determination of unexpected, significant or unacceptable risk to patients
- Failure to recruit patients at an acceptable rate
- Strong recommendation by the DSMB to the sponsor to stop the trial

X-6. Handling of missing or incoherent

In the case outcomes would be missing, binary missing outcomes will be treated as treatment failures in interim and primary final analyses, with an imputation by last value carried forward as a sensitivity analysis. For time-to-event outcomes, they will be naturally handled using methods for censored data. No imputation will be used for secondary efficacy and safety outcomes.

X-7. Modifications to the statistical analysis plan

All modifications to the statistical analysis plan will be discussed between the principal investigator and the methodologist of the trial.

XI. DATA HANDLING AND RECORD KEEPING

XI-1. Data collection and entry

The data is collected on a specific medium in order to differentiate the data used for the study and the source data.

- Data collection

The data is collected in an eCRF. Data collection will be common for all participating countries.

- Computerization of data

For all participating countries, the data is collected via the CleanWEB business application (Telemedicine Technologies, Boulogne Billancourt, France). This solution meets the requirements of the various regulations GCP, ICH, 21 CFR part 11 (FDA) in terms of identification, authentication, traceability, encryption of data flows and data hosting.

XI-2. Data transfer

The data collected in an e-CRF is transmitted in a secure manner (encryption of the data file or encryption of the data flow).

The data to support research results may be shared with the journal / open access for meta-analysis

XI-3. Modification, validation and verification of data

Changes are tracked automatically via an audit trail tracing the identity of the user, logging of the connection, and the nature of the change.

The database lock can only take place after a reconciliation between the monitoring data, the safety data (safety base) and those entered in the CRF (clinical base). This reconciliation allows:

- identify deviations from the protocol;
- identify the patients wrongly included in the regulations;
- measure the CRF completion rate;
- analyse the queries in progress.

Data management is carried out before statistical analysis in a succinct and automatic manner for consistency checks and missing data.

XI-4. Record keeping and archiving

(details in appendix)

XII. REGULATORY, ETHICAL CONSIDERATIONS

XII-1. Information and consent of the subject

The investigator orally informs and gives an information note to each subject, before their inclusion in the trial.

If the subject wishes to participate in the trial, he signs the consent before any intervention or procedure specified in the protocol. The investigator countersigns the consent. The signed and dated declaration of informed consent will remain at the investigator's site and must be safely archived by the investigator so that the forms can be retrieved at any time for monitoring, auditing and inspection

purposes. A copy of the signed and dated information and consent should be provided to the subject prior to participation.

XIII. QUALITY ASSURANCE AND QUALITY CONTROL

A Clinical Research Associate, appointed by the sponsor will ensure the follow-up and the proper conduct of the study, and in particular the review of the written data collected, their documentation, records and reports, in accordance with GCPs as well as the legislative and regulatory provisions in force.

(details in appendix)

XIV. PUBLICATION

The results of the study will be published in general publications.
The authors of the communication or publication will be defined in accordance with the international recommendations: International Committee of Medical Journal Editors "Uniform Requirements for Manuscripts Submitted to Biomedical Journals" Updated October 2007, in the absence of agreement between the investigators.

XV. REFERENCES

1. Libster R, Pérez Marc G, Wappner D, Coviello S, Bianchi A, Braem V, Esteban I, Caballero MT, Wood C, Berrueta M, Rondan A, Lescano G, et al. Early High-Titer Plasma Therapy to Prevent Severe Covid-19 in Older Adults. *New England Journal of Medicine* 2021;384:610–8.

2. Sullivan DJ, Gebo KA, Shoham S, Bloch EM, Lau B, Shenoy AG, Mosnaim GS, Gniadek TJ, Fukuta Y, Patel B, Heath SL, Levine AC, et al. Randomized Controlled Trial of Early Outpatient COVID-19 Treatment with High-Titer Convalescent Plasma. *medRxiv* 2021;2021.12.10.21267485.

3. Millat-Martinez P, Gharbharan A, Alemany A, Rokx C, Geurtsvankessel C, Papageourgiou G, van Geloven N, Jordans C, Groeneveld G, Swaneveld F, van der Schoot E, Corbacho-Monné M, et al. Convalescent plasma for outpatients with early COVID-19. *medRxiv* 2021;2021.11.30.21266810.

4. Korley FK, Durkalski-Mauldin V, Yeatts SD, Schulman K, Davenport RD, Dumont LJ, El Kassar N, Foster LD, Hah JM, Jaiswal S, Kaplan A, Lowell E, et al. Early Convalescent Plasma for High-Risk Outpatients with Covid-19. *N Engl J Med* 2021;NEJMoa2103784.

5. Hueso T, Pouderoux C, Péré H, Beaumont AL, Raillon LA, Ader F, Chatenoud L, Eshagh D, Szwebel TA, Martinot M, Camou F, Crickx E, et al. Convalescent plasma therapy for B-cell-depleted patients with protracted COVID-19. *Blood* 2020;12;136(20):2290-2295.

6. Thompson MA, Henderson JP, Shah PK, Rubinstein SM, Joyner MJ, Choueiri TK, Flora DB, Griffiths EA, Gulati AP, Hwang C, Koshkin VS, Papadopoulos EB, et al. Association of Convalescent Plasma Therapy With Survival in Patients With Hematologic Cancers and COVID-19. *JAMA Oncology [Internet]* 2021 [cited 2021 Aug 7];Available from: https://doi.org/10.1001/jamaoncol.2021.1799

7. Stamatatos L, Czartoski J, Wan Y-H, Homad LJ, Rubin V, Glantz H, Neradilek M, Seydoux E, Jennewein MF, MacCamy AJ, Feng J, Mize G, et al. mRNA vaccination boosts cross-variant neutralizing antibodies elicited by SARS-CoV-2 infection. *Science* 2021;372:1413–8.

8. Lustig Y, Nemet I, Kliker L, Zuckerman N, Yishai R, Alroy-Preis S, Mendelson E, Mandelboim M. Neutralizing Response against Variants after SARS-CoV-2 Infection and One Dose of BNT162b2. *New England Journal of Medicine* 2021;384:2453–4.

9. Shah V, Ko Ko T, Zuckerman M, Vidler J, Sharif S, Mehra V, Gandhi S, Kuhnl A, Yallop D, Avenoso D, Rice C, Sanderson R, et al. Poor outcome and prolonged persistence of SARS-CoV-2 RNA in COVID-19 patients with haematological malignancies; King's College Hospital experience. *Br J Haematol* 2020;10.1111/bjh.16935.

10. Aydillo T, Gonzalez-Reiche AS, Aslam S, van de Guchte A, Khan Z, Obla A, Dutta J, van Bakel H, Aberg J, García-Sastre A, Shah G, Hohl T, et al. Shedding of Viable SARS-CoV-2 after Immunosuppressive Therapy for Cancer. *New England Journal of Medicine* 2020;383:2586–8.

11. Reñosa MDC, Landicho J, Wachinger J, Dalglish SL, Bärnighausen K, Bärnighausen T, McMahon SA. Nudging toward vaccination: a systematic review. *BMJ Glob Health* 2021;6:e006237.

12. Bar-On YM, Goldberg Y, Mandel M, Bodenheimer O, Freedman L, Kalkstein N, Mizrahi B, Alroy-Preis S, Ash N, Milo R, Huppert A. Protection of BNT162b2 Vaccine Booster against Covid-19 in Israel. *N Engl J Med* 2021;NEJMoa2114255.

13. Ciotti M, Ciccozzi M, Pieri M, Bernardini S. The COVID-19 pandemic: viral variants and vaccine efficacy. *Critical Reviews in Clinical Laboratory Sciences* 2021;0:1–10.

14. Schrezenmeier E, Bergfeld L, Hillus D, Lippert J-D, Weber U, Tober-Lau P, Landgraf I, Schwarz T, Kappert K, Stefanski A-L, Sattler A, Kotsch K, et al. Immunogenicity of COVID-19 Tozinameran Vaccination in Patients on Chronic Dialysis. *Front Immunol* 2021;12:690698.

15. Sattler A, Schrezenmeier E, Weber UA, Potekhin A, Bachmann F, Straub-Hohenbleicher H, Budde K, Storz E, Proß V, Bergmann Y, Thole LML, Tizian C, et al. Impaired humoral and cellular immunity after SARS-CoV-2 BNT162b2 (tozinameran) prime-boost vaccination in kidney transplant recipients. *J Clin Invest* 131:e150175.

16. Rincon-Arevalo H, Choi M, Stefanski A-L, Halleck F, Weber U, Szelinski F, Jahrsdörfer B, Schrezenmeier H, Ludwig C, Sattler A, Kotsch K, Potekhin A, et al. Impaired humoral immunity to SARS-CoV-2 BNT162b2 vaccine in kidney transplant recipients and dialysis patients. *Science Immunology* 2021;6:eabj1031.

17. Herishanu Y, Avivi I, Aharon A, Shefer G, Levi S, Bronstein Y, Morales M, Ziv T, Shorer Arbel Y, Scarfò L, Joffe E, Perry C, et al. Efficacy of the BNT162b2 mRNA COVID-19 vaccine in patients with chronic lymphocytic leukemia. *Blood* 2021;137:3165–73.

18. Williamson EJ, Walker AJ, Bhaskaran K, Bacon S, Bates C, Morton CE, Curtis HJ, Mehrkar A, Evans D, Inglesby P, Cockburn J, McDonald HI, et al. OpenSAFELY: factors associated with COVID-19 death in 17 million patients. *Nature* 2020;584:430–6.

19. Rosenbaum L. The Untold Toll — The Pandemic's Effects on Patients without Covid-19. *New England Journal of Medicine* 2020;382:2368–71.

20. Raja MA, Mendoza MA, Villavicencio A, Anjan S, Reynolds JM, Kittipibul V, Fernandez A, Guerra G, Camargo JF, Simkins J, Morris MI, Abbo LA, et al. COVID-19 in solid organ transplant recipients: A systematic review and meta-analysis of current literature. *Transplantation Reviews* 2021;35:100588.

21. Zhang J, Wang X, Jia X, Li J, Hu K, Chen G, Wei J, Gong Z, Zhou C, Yu H, Yu M, Lei H, et al. Risk factors for disease severity, unimprovement, and mortality in COVID-19 patients in Wuhan, China. *Clin Microbiol Infect* 2020;26:767–72.

22. Guidance on shielding and protecting people who are clinically extremely vulnerable from COVID-19 [Internet]. *GOV.UK* [cited 2021 Jul 5];Available from: https://www.gov.uk/government/publications/guidance-on-shielding-and-protecting-extremely-vulnerable-persons-from-covid-19/guidance-on-shielding-and-protecting-extremely-vulnerable-persons-from-covid-19

23. Avetisyan G, Aschan J, Hassan M, Ljungman P. Evaluation of immune responses to seasonal influenza vaccination in healthy volunteers and in patients after stem cell transplantation. *Transplantation* 2008;86:257–63.

24. Ripoll JG, van Helmond N, Senefeld JW, Wiggins CC, Klassen SA, Baker SE, Larson KF, Murphy BM, Andersen KJ, Ford SK, Casadevall A, Joyner MJ. Convalescent Plasma for Infectious Diseases: Historical Framework and Use in COVID-19. *Clin Microbiol Newsl* 2021;43:23–32.

25. Mair-Jenkins J, Saavedra-Campos M, Baillie JK, Cleary P, Khaw F-M, Lim WS, Makki S, Rooney KD, Nguyen-Van-Tam JS, Beck CR, Mateus ALP, Reuter S, et al. The Effectiveness of Convalescent Plasma and Hyperimmune Immunoglobulin for the Treatment of Severe Acute Respiratory Infections of Viral Etiology: A Systematic Review and Exploratory Meta-analysis. *J Infect Dis* 2015;211:80–90.

26. Janiaud P, Axfors C, Schmitt AM, Gloy V, Ebrahimi F, Hepprich M, Smith ER, Haber NA, Khanna N, Moher D, Goodman SN, Ioannidis JPA, et al. Association of Convalescent Plasma Treatment With Clinical Outcomes in Patients With COVID-19: A Systematic Review and Meta-analysis. *JAMA* 2021;325:1185–95.

27. Convalescent plasma in patients admitted to hospital with COVID-19 (RECOVERY): a randomised controlled, open-label, platform trial. *Lancet* 2021;397:2049–59.

28. Li L, Zhang W, Hu Y, Tong X, Zheng S, Yang J, Kong Y, Ren L, Wei Q, Mei H, Hu C, Tao C, et al. Effect of Convalescent Plasma Therapy on Time to Clinical Improvement in Patients With Severe and Life-threatening COVID-19: A Randomized Clinical Trial. *JAMA* 2020;324:460–70.

29. Hamilton F, Lee T, Arnold D, Lilford R, Hemming K. Is convalescent plasma futile in COVID-19? A Bayesian re-analysis of the RECOVERY randomised controlled trial. *Int J Infect Dis* 2021;S1201-9712(21)00523-3.

30. Investigators TR-C, Estcourt LJ. Convalescent Plasma in Critically ill Patients with Covid-19. *medRxiv* 2021;2021.06.11.21258760.

31. de Candia P, Prattichizzo F, Garavelli S, La Grotta R, De Rosa A, Pontarelli A, Parrella R, Ceriello A, Matarese G. Effect of time and titer in convalescent plasma therapy for COVID-19. *iScience* 2021;24:102898.

32. Salazar E, Christensen PA, Graviss EA, Nguyen DT, Castillo B, Chen J, Lopez BV, Eagar TN, Yi X, Zhao P, Rogers J, Shehabeldin A, et al. Significantly Decreased Mortality in a Large Cohort of Coronavirus Disease 2019 (COVID-19) Patients Transfused Early with Convalescent Plasma Containing High-Titer Anti–Severe Acute Respiratory Syndrome Coronavirus 2 (SARS-CoV-2) Spike Protein IgG. *Am J Pathol* 2021;191:90–107.

33. Sullivan DJ, Gebo KA, Shoham S, Bloch EM, Lau B, Shenoy AG, Mosnaim GS, Gniadek TJ, Fukuta Y, Patel B, Heath SL, Levine AC, et al. Early Outpatient Treatment for Covid-19 with Convalescent Plasma. *N Engl J Med* 2022;NEJMoa2119657.

34. Senefeld JW, Klassen SA, Ford SK, Senese KA, Wiggins CC, Bostrom BC, Thompson MA, Baker SE, Nicholson WT, Johnson PW, Carter RE, Henderson JP, et al. Use of convalescent plasma in COVID-19 patients with immunosuppression. *Transfusion* 2021;10.1111/trf.16525.

35. Piechotta V, Iannizzi C, Chai KL, Valk SJ, Kimber C, Dorando E, Monsef I, Wood EM, Lamikanra AA, Roberts DJ, McQuilten Z, So-Osman C, et al. Convalescent plasma or hyperimmune immunoglobulin for people with COVID-19: a living systematic review. *Cochrane Database Syst Rev* 2021;5:CD013600.

36. Larsen MD, de Graaf EL, Sonneveld ME, Plomp HR, Nouta J, Hoepel W, Chen H-J, Linty F, Visser R, Brinkhaus M, Šuštić T, de Taeye SW, et al. Afucosylated IgG characterizes enveloped viral responses and correlates with COVID-19 severity. *Science* 2021;371:eabc8378.

37. Chen P, Nirula A, Heller B, Gottlieb RL, Boscia J, Morris J, Huhn G, Cardona J, Mocherla B, Stosor V, Shawa I, Adams AC, et al. SARS-CoV-2 Neutralizing Antibody LY-CoV555 in Outpatients with Covid-19. *New England Journal of Medicine* 2021;384:229–37.

38. Weinreich DM, Sivapalasingam S, Norton T, Ali S, Gao H, Bhore R, Musser BJ, Soo Y, Rofail D, Im J, Perry C, Pan C, et al. REGN-COV2, a Neutralizing Antibody Cocktail, in Outpatients with Covid-19. *New England Journal of Medicine* 2021;384:238–51.

39. Joyner MJ, Carter RE, Senefeld JW, Klassen SA, Mills JR, Johnson PW, Theel ES, Wiggins CC, Bruno KA, Klompas AM, Lesser ER, Kunze KL, et al. Convalescent Plasma Antibody Levels and the Risk of Death from Covid-19. *New England Journal of Medicine* 2021;384:1015–27.

40. Körper S, Weiss M, Zickler D, Wiesmann T, Zacharowski K, M.Corman V, Grüner B, Ernst L, Spieth P, Lepper PM, Bentz M, Zinn S, et al. High Dose Convalescent Plasma in COVID-19: Results from the Randomized Trial CAPSID. *medRxiv* 2021;2021.05.10.21256192.

41. Wang P, Nair MS, Liu L, Iketani S, Luo Y, Guo Y, Wang M, Yu J, Zhang B, Kwong PD, Graham BS, Mascola JR, et al. Antibody resistance of SARS-CoV-2 variants B.1.351 and B.1.1.7. *Nature* 2021;593:130–5.

42. ANSM. 17ème Rapport national d'hémovigilance [Internet]. 2019;Available from: https://ansm.sante.fr/actualites/lansm-publie-le-rapport-dactivite-hemovigilance-2019

43. Kemp SA, Collier DA, Datir RP, Ferreira IA, Gayed S, Jahun A, Hosmillo M, Rees-Spear C, Mlcochova P, Lumb IU, Roberts DJ, Chandra A, et al. SARS-CoV-2 evolution during treatment of chronic infection. *Nature* 2021;592:277–82.

44. Martinot M, Jary A, Fafi-Kremer S, Leducq V, Delagreverie H, Garnier M, Pacanowski J, Mékinian A, Pirenne F, Tiberghien P, Calvez V, Humbrecht C, et al. Remdesivir failure with SARS-CoV-2 RNA-dependent RNA-polymerase mutation in a B-cell immunodeficient patient with protracted Covid-19. *Clin Infect Dis* 2020;ciaa1474.

45. Chen RE, Winkler ES, Case JB, Aziati ID, Bricker TL, Joshi A, Darling TL, Ying B, Errico JM, Shrihari S, VanBlargan LA, Xie X, et al. In vivo monoclonal antibody efficacy against SARS-CoV-2 variant strains. *Nature* 2021;596:103–8.

46. Chen RE, Zhang X, Case JB, Winkler ES, Liu Y, VanBlargan LA, Liu J, Errico JM, Xie X, Suryadevara N, Gilchuk P, Zost SJ, et al. Resistance of SARS-CoV-2 variants to neutralization by monoclonal and serum-derived polyclonal antibodies. *Nat Med* 2021;27:717–26.

47. Kutzler HL, Kuzaro HA, Serrano OK, Feingold A, Morgan G, Cheema F. Initial experience of bamlanivimab monotherapy use in solid organ transplant recipients. *Transplant Infectious Disease* n/a:e13662.

48. Corti D, Purcell LA, Snell G, Veesler D. Tackling COVID-19 with neutralizing monoclonal antibodies. *Cell* 2021;184:3086–108.

49. Hoffmann M, Arora P, Groß R, Seidel A, Hörnich BF, Hahn AS, Krüger N, Graichen L, Hofmann-Winkler H, Kempf A, Winkler MS, Schulz S, et al. SARS-CoV-2 variants B.1.351 and P.1 escape from neutralizing antibodies. *Cell* 2021;184:2384-2393.e12.

50. Hoffmann M, Hofmann-Winkler H, Krüger N, Kempf A, Nehlmeier I, Graichen L, Arora P, Sidarovich A, Moldenhauer A-S, Winkler MS, Schulz S, Jäck H-M, et al. SARS-CoV-2 variant B.1.617 is resistant to bamlanivimab and evades antibodies induced by infection and vaccination. *Cell Rep* 2021;36:109415.

51. Choudhary MC, Chew KW, Deo R, Flynn JP, Regan J, Crain CR, Moser C, Hughes M, Ritz J, Ribeiro RM, Ke R, Dragavon JA, et al. Emergence of SARS-CoV-2 Resistance with Monoclonal Antibody Therapy. *medRxiv* 2021;2021.09.03.21263105.

52. Peiffer-Smadja N, Bridier-Nahmias A, Ferré VM, Charpentier C, Garé M, Rioux C, Allemand A, Lavallée P, Ghosn J, Kramer L, Descamps D, Yazdanpanah Y, et al. Emergence of E484K Mutation Following Bamlanivimab Monotherapy among High-Risk Patients Infected with the Alpha Variant of SARS-CoV-2. *Viruses* 2021;13:1642.

53. Planas D, Veyer D, Baidaliuk A, Staropoli I, Guivel-Benhassine F, Rajah MM, Planchais C, Porrot F, Robillard N, Puech J, Prot M, Gallais F, et al. Reduced sensitivity of SARS-CoV-2 variant Delta to antibody neutralization. *Nature* 2021;596:276–80.

54. Wilhelm A, Widera M, Grikscheit K, Toptan T, Schenk B, Pallas C, Metzler M, Kohmer N, Hoehl S, Helfritz FA, Wolf T, Goetsch U, et al. Reduced Neutralization of SARS-CoV-2 Omicron Variant by Vaccine Sera and monoclonal antibodies. *medRxiv* 2021;2021.12.07.21267432.

55. Lee WS, Wheatley AK, Kent SJ, DeKosky BJ. Antibody-dependent enhancement and SARS-CoV-2 vaccines and therapies. *Nat Microbiol* 2020;5:1185–91.

56. Cele S, Jackson L, Khoury DS, Khan K, Moyo-Gwete T, Tegally H, San JE, Cromer D, Scheepers C, Amoako D, Karim F, Bernstein M, et al. SARS-CoV-2 Omicron has extensive but incomplete escape of Pfizer BNT162b2 elicited neutralization and requires ACE2 for infection. *medRxiv* 2021;2021.12.08.21267417.

57. Rössler A, Riepler L, Bante D, Laer D von, Kimpel J. SARS-CoV-2 B.1.1.529 variant (Omicron) evades neutralization by sera from vaccinated and convalescent individuals. *medRxiv* 2021;2021.12.08.21267491.

58. Bates TA, McBride SK, Winders B, Schoen D, Trautmann L, Curlin ME, Tafesse FG. Antibody Response and Variant Cross-Neutralization After SARS-CoV-2 Breakthrough Infection. *JAMA* 2022;327:179–81.

59. WHO Working Group on the Clinical Characterisation and Management of COVID-19 infection. A minimal common outcome measure set for COVID-19 clinical research. *Lancet Infect Dis* 2020;20:e192–7.

60. Docherty AB, Harrison EM, Green CA, Hardwick HE, Pius R, Norman L. Features of 20 133 UK patients in hospital with covid-19 using the ISARIC WHO Clinical Characterisation Protocol: prospective observational cohort study. *BMJ* 2020;369:m1985.

61. Mehta CR, Pocock SJ. Adaptive increase in sample size when interim results are promising: a practical guide with examples. *Stat Med* 2011;30:3267–84.

A Randomised Open-Label Trial of Early, Very High-Titre Convalescent Plasma Therapy in Clinically Vulnerable Individuals with Mild COVID-19

Authorised and unauthorised medications

(Appendix to Clinical Trial Protocol VII-7)

COVIC-19 study

COVIC-19

Appendix Version 2, August 9th 2022

Authorised medications

The following COVID-19 medications are authorised for use as standard of care in patients enrolled in the study as pre-exposure prophylaxis, post-exposure prophylaxis, as well as early treatment.

Monoclonal antibodies

- Casirivimab
- Casirivimab/imdevimab (REGN-COV2 or Ronapreve)
- Bamlanivimab
- Bamlanivimab/etesevimab
- Etesevimab
- Imdevimab
- Regdanvimab (Regkirona)
- Sotrovimab (Xevudy)
- Tixagevimab/cilgavimab (Evusheld)

Antiviral drugs

- Molnupiravir (MK-4482)
- Nirmatrelvir/ritonavir (Paxlovid)
- Remdesivir

These medications must be used according to their approval or according to current treatment guidelines (e.g. for Germany refer to www.rki.de/covid-19-therapie).

The study intervention is performed in addition to usual care. If a patient receives both monoclonal antibodies and CCP according to the assignment to the CCP group, the infusion of CCP and monoclonal antibodies may be administered in either order (CCP first or monoclonal antibodies first). However, the infusion of monoclonal antibodies and the transfusion of plasma should not be given simultaneously. To identify and assign acute reactions, a minimum interval of 1 hour must be observed between the end of the administration of one therapy and the start of the next. If an oral antiviral is given in addition to the CCP, the CCP can be transfused any time. Start and end times of administration are documented in the eCRF.

Centres should ensure that medications used as standard of care are used similarly for patients in both treatment arms. A clinical trial centre's standard of care is determined at the start of the study. Any changes are to be reported to the sponsor.

Information on standard of care treatment (type of treatment, drug name, start date / end date, dose) will be recorded in the electronic case report form. It will be monitored, and an explanation must be provided in case of deviation from the established standard of care in a clinical trial centre.

Unauthorised medications

All other COVID-19 medications are not authorised from the moment of inclusion and up to the study primary endpoint (except if warranted for a reason other than COVID-19). They may be used once the primary endpoint is reached. There are no unauthorised treatment or medication after achievement of the primary endpoint.

We provide the following list of unauthorised medications as a reference. Unauthorised medications are but not limited to:

- Anti-IL-6R monoclonal antibodies such as tocilizumab
- Baricitinib (JAK 1 and JAK 2 inhibitor)
- Corticosteroids – if given for treatment of COVID-19

List updates

The trial scientific committee may update this list whenever necessary to ensure that trial participants receive state of the art standard of care at the time of inclusion. Occasions for a review of this list of authorized COVID-19 specific treatments include, but are not limited to, approval of new specific COVID therapeutics or changes in National Guidelines for patient populations that are also eligible for this study.

The members of the scientific committee are:

- Thomas Appl
- Daniel Bradshaw
- Maxime Desmarets
- Lise Estcourt
- Sixten Körper
- Bart Rijnders
- David Roberts
- Erhard Seifried
- Hubert Schrezenmeier
- Pierre Tiberghien
- Eric Toussirot

Upon update, incremental versions of the document will be created with mention of the date from which the document applies. **Study centres will be informed immediately.**

Updates to this Appendix will not be considered a substantial amendment of the protocol and will not be submitted for regulatory / ethical approval as long as the above conditions are met (except for France).
